# Stereocontrolled Synthesis and Conformational Analysis of a Series of Disaccharides α,β-d-GlcA-(1→3)-α-L-Fuc

**DOI:** 10.3390/molecules28227571

**Published:** 2023-11-13

**Authors:** Alexey G. Gerbst, Dmitry Z. Vinnitsky, Alexandra I. Tokatly, Andrey S. Dmitrenok, Vadim B. Krylov, Nadezhda E. Ustuzhanina, Nikolay E. Nifantiev

**Affiliations:** 1 Laboratory of Glycoconjugate Chemistry, N.D. Zelinsky Institute of Organic Chemistry, Russian Academy of Sciences, Leninsky pr. 47, Moscow 119991, Russia; phalereus@mail.ru (D.Z.V.); dmt@ioc.ac.ru (A.S.D.); nen@ioc.ac.ru (N.E.N.); 2 Laboratory of Synthetic Glycovaccines, N.D. Zelinsky Institute of Organic Chemistry, Russian Academy of Sciences, Leninsky pr. 47, Moscow 119991, Russia

**Keywords:** glucuronic acid, conformational studies, DFT, quantum chemistry, stereocontrol, glycosylation, hydrogen bond-mediated aglycone delivery

## Abstract

D-Glucuronic acid is a fundamental building block of many biologically important polysaccharides, either in its non-substituted form or bearing a variety of substituents, among them sulfates. We have previously performed a study of the effects of exhaustive sulfation on the conformational behavior of β-gluronopyranosides. Herein, we report an investigation comparing α- and β-derivatives of this monosaccharide within the title disaccharides using NMR and quantum chemistry approaches. It was found that for α-linked disaccharides, the introduction of sulfates did not greatly affect their conformational behavior. However, for β-derivatives, considerable conformational changes were observed. In general, they resemble those that took place for the monosaccharides, except that NOESY experiments and calculations of intra-ring spin–spin coupling constants suggest the presence of a ^1^*S*_5_ conformer along with ^3^*S*_1_ in the fully sulfated disaccharide. During the synthesis of model compounds, hydrogen bond-mediated aglycone delivery was used as an α-directing stereocontrol approach in the glucuronidation reaction.

## 1. Introduction

**Figure 1:**
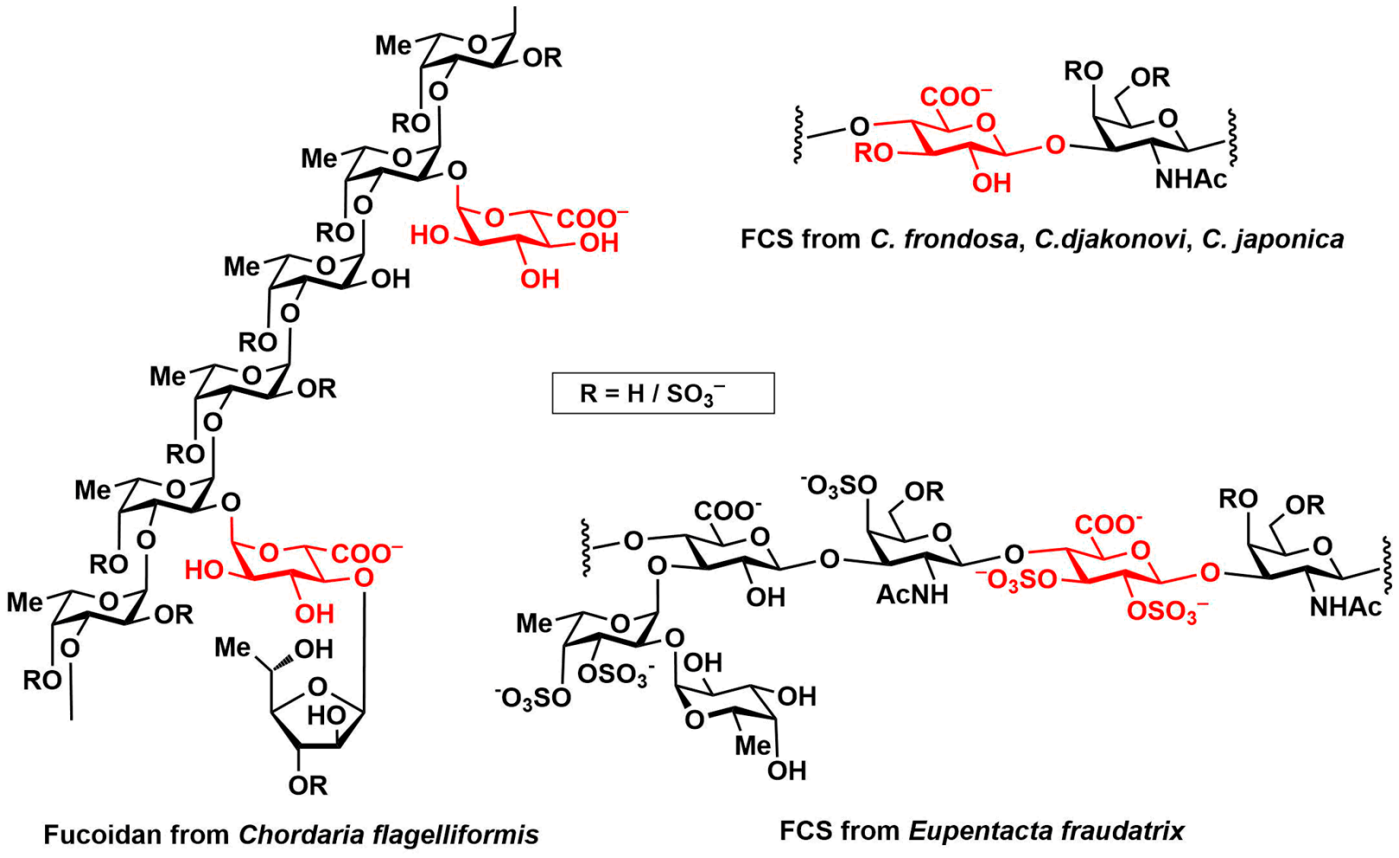
Fragments of polysaccharides bearing GlcA units.

d-Glucuronic acid is an important building block which can be found in a great number of natural compounds [1,2,3], some of them with promising biological activity profiles [4,5]. In these compounds, GlcA can be found in both α- and β-form. Glycosaminoglycans (chondroitin sulfates [6], heparan sulfates [7], hyaluronic acid [8], fucosylated chondroitin sulfates [9,10]) are one of the most promising and intensively investigated classes bearing β-d-GlcA residues. Previously, we encountered an interesting unit in fucosylated chondroitin sulfate, isolated from the sea cucumber *Eupentacta fraudatrix* [11]. Its structure was determined as →4)-β-d-GlcpA2S3S-(1→3)-β-d-GalpNAc6S-(1→ (Figure 1), and its GlcA residue’s chemical shifts and coupling constants were quite unusual. They differed from the spectral data of similar polysaccharides derived from *Cucumaria frondosa* [12], *C. djakonovi* [13], and *C. japonica* [14], which contained the same fragment but were either without sulfates or monosulfated at O-3. In order to gain insight into the biological importance of these unusual structures? we performed a thorough investigation of the conformation of a fully sulfated β-d-GlcA residue employing a series of synthetic monosaccharides [15]. The obtained data clearly indicated that two skew-boat conformers, ^O^*S*_2_ and ^3^*S*_1_, provided a significant contribution to the conformational equilibrium and could, in turn, affect intramolecular interactions.

Although the α-d-GlcA unit is more rarely encountered in natural compounds than the β-isomer, some of its compounds have prospects for practical application. In particular, fucoidans (polysaccharides with a backbone built of α-L-fucose residues) are already being actively investigated as potential medical agents [16]. There are a few known examples of fucoidans bearing α-d-GlcA as a side chain [17,18,19,20]. The structure of one of them, fucoidan isolated from the seaweed *Chordaria flagelliformis*, is shown in Figure 1. In natural fucoidans, the GlcA units, unlike fucose ones, do not bear any sulfate groups. It is noteworthy that chemical sulfation significantly changes the effect of modified fucoidans on blood coagulation and platelet aggregation [21]. This is probably related to the increased charge density, but conformational changes should not be discarded as an explanation at once.

**Figure 2:**
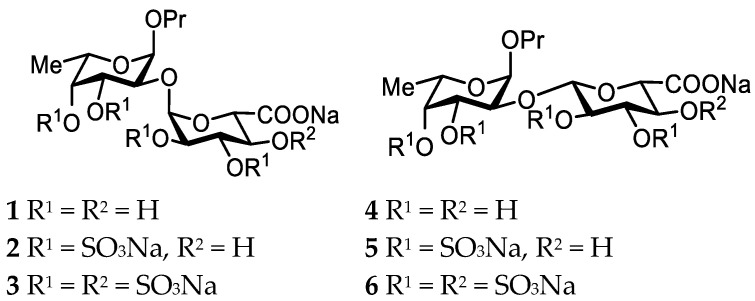
Model compounds studied in this work.

In order to investigate this possibility, in the present study the scope of objects has been expanded to include disaccharides with glucuronic acid as the glycosylating residue. Both α- and β-configurations are investigated. The model compounds **1**–**6** used in this work are shown in Figure 2. During the synthesis, we also tried to address the problem of α-directing stereocontrol in the glucuronidation reaction. We performed a series of model glycosylations varying the substituent at *O*-4 in the glucuronic donors to examine the application of the hydrogen bond-mediated aglycone delivery (HAD) stereocontrol approach using the picoloyl protecting group suggested by Yasomanee and Demchenko in 2012 [22].

## 2. Results and Discussion

**Scheme 1:**
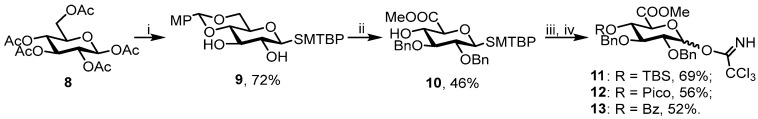
Synthesis of glucuronic acid donors **11**–**13**. Reagents and conditions: i: 1. MTBTP, BF_3_·Et_2_O, DCM, 2. MeONa, MeOH, 3. 4-anisaldehyde, HC(OMe)_3_, CSA, CH_3_CN; ii: 1. BnBr, NaH, DMF, 2. TFA, 3. BAIB, TEMPO, DCM/H_2_O 5:1, 0 °C, 4. MeI, K_2_CO_3_, DMF; iii: a. TBSOTf, NEt_3_, DCM; b. PicoA, DCC, DMAP, DCM; c. BzCl, NEt_3_, DCM iv: 1. NBS, H_2_O/acetone, 2. TCAN, Cs_2_CO_3_, DCM.

In order to carry out NMR and conformational studies, we synthesized six model compounds—α-linked disaccharides **1**–**3** and β-linked disaccharides **4**–**6**. For both types of linkages, we obtained non-sulfated, fully sulfated, and partially sulfated disaccharides. As a model acceptor, allyl α-L-fucopyranoside **7** bearing 3,4-*O*-isopropylidene protection was chosen. For the synthesis of glucuronic acid donors, we used commercially available β-d-glucose pentaacetate **8**. In the first step, 5-*tert*-butyl-2-methylbenzenethiol (MTBTP) was introduced in the anomeric position and the product was treated with MeONa in methanol followed by the installation of 4,6-*para*-methoxybenzylidene protection to give diol **9** with good yield (72%). The latter was benzylated with NaH and BnBr in DMF, the diol protection was cleaved in acidic conditions, and the primary hydroxyl group was oxidized with TEMPO and BAIB in a DCM/water mixture. The uronic acid intermediate product was treated with MeI and K_2_CO_3_ in DMF to give methyl ester **10** with an overall yield of 46%. The remaining hydroxyl group was substituted with TBS, picoloyl, or benzoyl protection. Then, the anomeric thiophenol was cleaved employing NBS in acetone/water, followed by treatment with TCAN and cesium carbonate in DCM to give trichloroacetimidate donors **11**–**13** with moderate yields of 52–69% (Scheme 1).

**Scheme 2:**
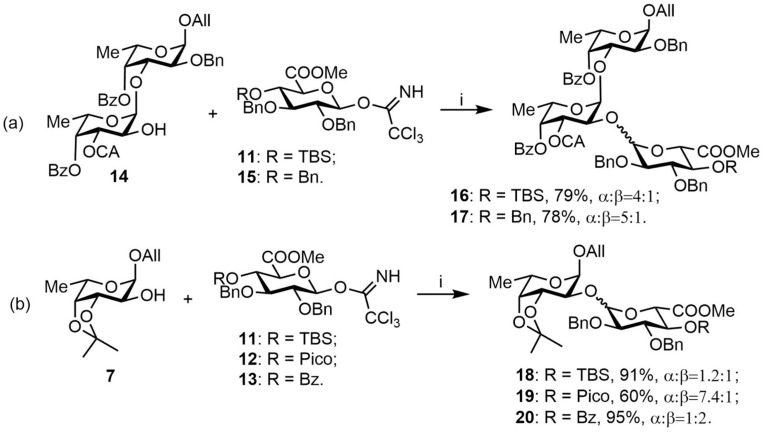
Model glycosylations with glycosyl acceptors **14** (**a**) and **7** (**b**). Reagents and conditions: i: TMOSTf, MS-4Å, DCM.

Efficient α-directing stereocontrol in glucuronidation reactions is still a challenging problem [23,24]. Early on in the synthesis of fucoidan-related oligosaccharides bearing α-d-GlcA units [25], we used 2,3-di-*O*-benzyl-4-*O*-*tert*-butyldimethylsilyl **11** and 2,3,4-tri-*O*-benzyl **15** trichloroacetimidate donors and achieved α:β ratios of 4:1 and 5:1, respectively (Scheme 2a). In this model glycosylation, we decided to employ the HAD concept, based on a preliminary coordination between donor and acceptor due to hydrogen bond formation [22]. This is the second attempt to elaborate this approach on uronic donors. Previously, Demchenko employed it to obtain a β-(1→3)-linked mannuronic tetrasaccharide [26].

Out of our three trichloroacetimidate donors bearing 4-*O*-TBS-, 4-*O*-Pico-, and 4-*O*-Bz-protecting groups (**11**, **12**, **13**, respectively), the first one was chosen to connect our new dataset with the results obtained earlier, while the third one was used as a non-coordinating analogue of the picoloyl group. All new glycosylation reactions were performed at the same conditions with 2 h preliminary stirring, which is required for coordination between donor and acceptor. The obtained results clearly indicate that the HAD approach is a prospective method for α-directing glycosylation stereocontrol for our GlcA-donors—the α:β ratio in the case of 4-*O*-picoloyl donor **12** was determined to be 7.4:1 compared to 1.2:1 for 4-*O*-TBS donor **11** and 1:2 for 4-*O*-Bz donor **13** (Scheme 2b). However, the yield in case of the HAD approach was significantly lower (60%). We anticipate that in the case of oligosaccharides and more hindered acceptors, the ratio would be even more favorable towards α-glycosylation, based on the comparison of acceptors **14** and **7** as glycosylated by donor **11**.

**Scheme 3:**
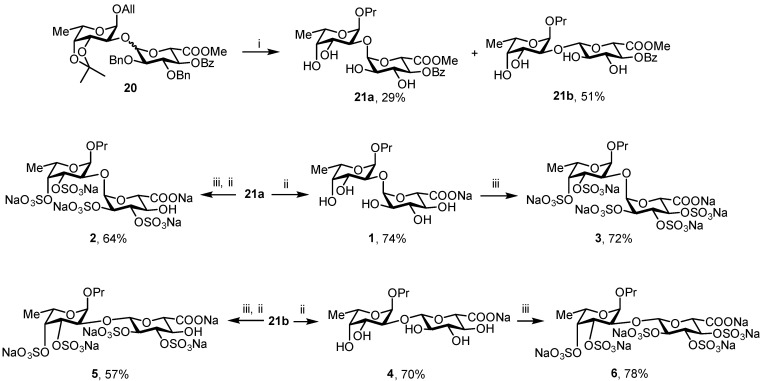
Synthesis of model compounds **1**–**6**. Reagents and conditions: i: 1. TFA, DCM, 2. H_2_, Pd/C, EtOAc/MeOH. ii: LiOH 1 M, H_2_O; iii: 1. Py·SO_3_, DMF, 2. NaHCO_3_, MeOH.

For the subsequent synthesis of model compounds **1**–**6,** only disaccharide **20** was used (Scheme 3). After cleavage of 3,4-*O*-isopropylidene protection in acidic conditions followed by hydrogenolysis, α- and β-isomers were separated to give tetraols **21a** and **21b** with an overall yield of 80% for both isomers. Final saponification of **21a** and **21b** with LiOH 1 M gave disaccharides **1** (74%) and **4** (70%), respectively. Treatment of **1** and **4** with Py·SO_3_ in DMF gave fully sulfated compounds **3** (72%) and **6** (78%). To obtain the last couple of disaccharides, **21a** and **21b** were on the first step treated with Py·SO_3_ in DMF, followed by saponification with LiOH to give partially sulfated disaccharides **2** (64%) and **4** (57%).

The measured ^1^H NMR chemical shifts for the studied compounds **1**–**6** are given in Table 1. Inter-proton coupling constants for glucuronic acid residues (GlcA) are shown in Table 2 (coupling constants for fucose residues can be found in Section 3).

Preliminary analysis of chemical shifts for GlcA residues does not demonstrate any unexpected changes upon the introduction of sulfate groups in the α-series (structures **1**–**3**). However, some decrease in coupling constants can be observed for compound **3** (Table 2). For the β-series, the situation is different, resembling that which was observed in the case of GlcA monosaccharides. When comparing structures **4** and **6**, it can be seen that drastic changes both in chemical shifts and coupling constants occur to protons H-3, H-4, and H-5. Besides the sulfation effect caused by the introduction of the electronegative sulfate group, they may arise from conformational changes. To explain the observed effects and in continuation of our ongoing computer studies of carbohydrate conformations [27,28], a DFT analysis of the studied glucuronides was performed.

First, relaxed potential energy scans of ψ torsions of the glycosidic linkages were performed for both disaccharides at B3LYP/6-31G(d,p) level of theory. The scanned range was (−60°…+60°) with a 20° step. The φ angles were allowed to change during the constrained optimizations. This was in order to save computation time. This approach is justified since the behavior of the φ angles is mostly governed by the *exo*-anomeric effect and they have a more restricted range to change within. Since in our previous investigations the non-sulfated glucuronic acid residue was shown to exist exclusively in the ^4^*C*_1_ conformation, it was the only one considered for compounds **1** and **4**.

**Figure 3:**
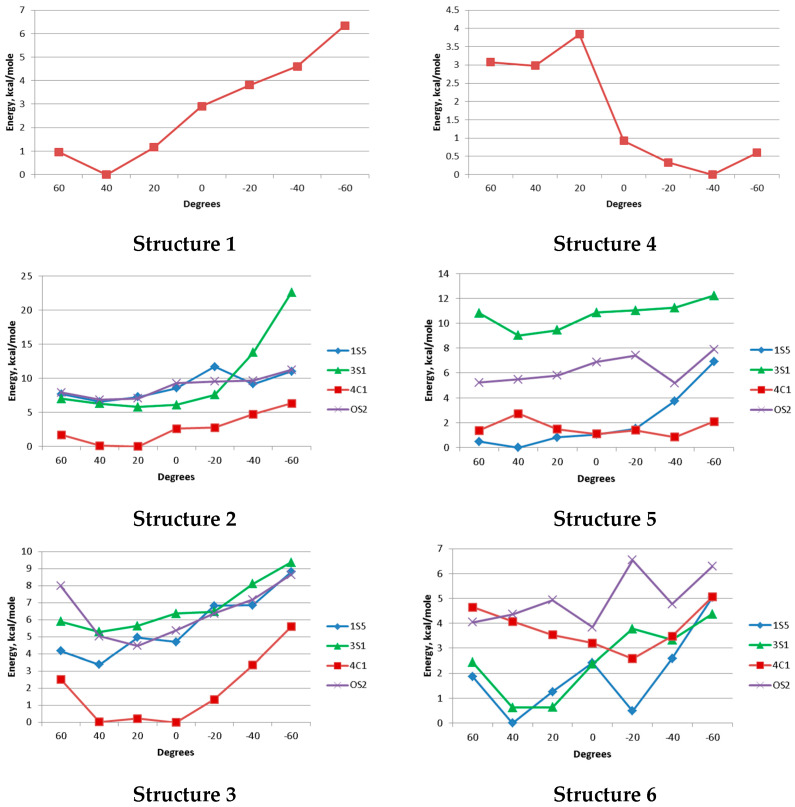
Scan graphs of the glycosidic linkages in structures **1**–**6** obtained at B3LYP/6-31G(d,p) level.

The resulting graphs are presented in Figure 3. They demonstrate that in the α-series, the ^4^*C*_1_ conformer always retains the lowest energy (structures **1**–**3**). However, in structure **5,** the ^1^*S*_5_ conformer has energy comparable to that of ^4^*C*_1_, and in structure **6** it seems to dominate along with ^3^*S*_1_. The minima found during these scans for the sulfated structures were reoptimized using M062X/def2-tzvp approximation. The resulting relative energies are shown in Table 3.

**Figure 4:**
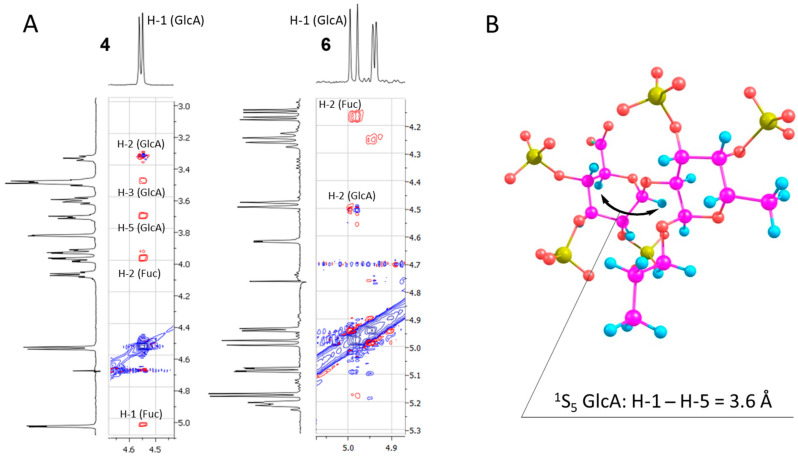
Fragments of 2D NOESY spectra of model disaccharides **4** and **6** (**A**). ^1^*S*_5_ conformer of compound **6**, atoms representation: hydrogen—cyan, carbon—magenta, oxygen—red, and sulfur—yellow (**B**).

The values shown in Table 3 confirm the tendency described above. While for disaccharides from the α-series the ^4^*C*_1_ conformer always has much lower energy than any skew-boat, for compound **5** and especially **6**, ^3^S_1_ and ^1^*S*_5_ become more preferred. This observation is additionally supported by 2D NOESY spectroscopy (Figure 4A, see full range NOESY spectra in Supplementary Materials). While in the case of non-sulfated β-disaccharide **4** cross-peaks corresponding to H-1/H-5 and H-1/H-3 intra-ring interactions in GlcA residue can be observed, they disappear in the spectrum of the fully sulfated compound **6**. Interestingly, an H-1/H-5 interaction was present in the case of monosaccharides. Here, its absence might be explained by the contribution from the ^1^*S*_5_ conformer where the H-1 and H-5 protons have a distance of about 3.6 Å between them due to the equatorial orientation of H-1 (Figure 4B).

Chemical shieldings were calculated for all conformers of structure **6** (Table 4). We used this work as an opportunity to test a recent DLPNO-MP2 method developed by the ORCA team [29] in its application to calculate NMR shielding constants for such highly sulfated systems. It was found that in the ^3^*S*_1_ conformer deshielding of H-3, H-4 and H-5 is present, while in ^1^*S*_5_ deshielding of H-4 occurs. This can serve as an explanation to the fact that lower field chemical shifts are observed in the NMR spectra of these compounds.

Additionally, intra-ring ^1^H-^1^H spin–spin coupling constants (*J*) were calculated for the revealed conformers of molecule **6**. The calculation was performed at the B3LYP/pcJ-2 level of theory. The results are presented in Table 5.

In the experimental spectra (Table 2), those *J*-values that could be definitely determined demonstrated a tendency towards a decrease in H-1/H-2, H-2/H-3, and H-4/H-5 interactions. From Table 5, it can be seen that in all the skew-boat conformers some constants also become lower as compared to the ^4^*C*_1_ conformer. Obviously, the experimentally observed *J*-values pattern does not correspond to a single conformer, as was also the case in the glucuronic acid monosaccharide. Thus, in the studied β-disaccharides, glucuronic acid retains conformational flexibility upon the introduction of sulfates and exhibits a prevalence of skew-boat conformers.

## 3. Materials and Methods

### 3.1. General Information

In this study, commercially purchased chemicals were used without purification unless noted. Standard procedures were used for the distilling and drying of solvents when necessary (DCM, MeOH, acetone). Some solvents were purchased as dry (DMF, THF, CH_3_CN, Sigma-Aldrich, Darmstadt, Germany) and used without additional manipulations. Dry solvents under argon atmosphere were used for chemical transformations involving air- or moisture-sensitive reagents. Aluminum sheets coated with silica gel 60 F_254_ (Merck) were used for thin-layer chromatography (TLC). The TLC plates were analyzed by UV light (λ = 254 nm) and developed with a mixture of 15% H_3_PO_4_ and orcinol (1.8 g/L) in EtOH/H_2_O (95:5, *v*/*v*) followed by heating (200 °C). Column chromatography was performed using Silica Gel 60 (40–63 μm, E. Merck). Solvents for column and thin layer chromatography are listed in volume-to-volume ratios. Sephadex G-15 column (400 × 16 mm) by elution with water at a flow rate of 0.5 mL/min was used for size-exclusion chromatography.

NMR spectra were measured on Bruker AM300 (300 MHz) or AV600 (600 MHz) spectrometers equipped with 5 mm pulsed-field-gradient (PFG) probes at temperatures denoted in the spectra in supplementary. Microtubes (Shigemi, Inc., Tokyo, Japan) were applied for sensitivity enhancement of small concentration probes. The resonance assignment in ^1^H and ^13^C NMR spectra was performed using various 2D experiments (e.g., COSY, NOESY, HSQC). Chemical shifts are reported in ppm referenced either to the solvent residual peaks as standard for chloroform (δ 7.26 ^1^H, δ 77.16 ^13^C) or methanol as internal standard for D_2_O (δ 3.34 ^1^H, δ 49.50 ^13^C). The ratio between isomers in glycosylation reactions was measured based on the ratio of integral intensities between GlcA residues’ anomeric correlations in HSQC spectrum.

Optical rotations were measured using a JASCO P-2000 polarimeter at ambient temperature (22–25 °C).

Bruker micrOTOF II instrument was used to record high-resolution mass spectra (HR MS) with electrospray ionization (ESI). The positive ion mode (interface capillary voltage −4500 V) or the negative ion mode (3200 V) were used; mass range was measured from *m*/*z* 50 to *m*/*z* 3000 Da; calibration was performed with Electrospray Calibrant Solution (Fluka). A syringe sample loading was apply for solutions in a mixture of acetonitrile and water (50:50, *v*/*v*, flow rate 3 μL/min). Nitrogen was used as a dry gas; interface temperature was set at 180 °C.

Geometry optimizations were performed using ORCA 5.0.2 program. RKS approximation with 6-31G(d,p) [30] basis set and D3 dispersion correction was employed for initial scans. Sulfates in the studied structures were treated as anions alone. CPCM model was applied with the built-in parameters for water. Geometry optimizations were performed until the RMS gradient reached a value less than 10^−4^. Further optimizations were conducted using M062X functional with def2-tzvp [31,32,33] basis set. Chemical shifts were calculated using DLPNO-MP2 approximation and gauge-independent atomic orbital approximation with PCSSEG-2 basis set, as described in work [34]. Spin–spin couplings were calculated using DFT approach with B3LYP functional and pcJ-2 [35] basis set. Only Fermi contact terms were considered.

### 3.2. Standard Procedures

#### 3.2.1. Substitution Thiophenyl with Trichloroacetimidate (Standard Procedure A)

To a solution of thioglycoside [0.2 mmol] in a mixture of acetone [1.8 mL] and H_2_O [0.2 mL] at 0 °C, NBS [0.6 mmol, 3 eq.] was added. Reaction mixture was stirred at 0 °C for 2 h. After, TLC showed full consumption of the 5 eq.

Starting material of the reaction mixture was diluted with EtOAc [50 mL] and washed with Na_2_S_2_O_3_ 1 M [50 mL] and H_2_O [50 mL]. The organic layer was dried (Na_2_SO_4_) and concentrated in vacuo. The residue was purified by chromatography (silica gel, eluent: hexane/EtOAc) to give hemiacetal. The latter was dissolved in DCM [2 mL], and Cs_2_CO_3_ [0.04 mmol, 0.2 eq.] and CCl_3_CN [0.24 mmol, 1.2 eq.] were added. Reaction mixture was stirred for 20 min. After TLC showed full consumption of the starting material, the mixture was purified by chromatography (silica gel neutralized with NEt_3_, eluent: hexane/EtOAc) to give a trichloroacetimidate product.

#### 3.2.2. Glycosylation (Standard Procedure B)

A mixture of a glycosyl acceptor [0.16 mmol] and a glycosyl donor [0.16 mmol, 1 eq.] was co-evaporated with toluene twice and dried for 1 h in vacuo, and then the flask was filled with Ar. Freshly activated molecular sieves 4 Å [100 mg] and anhydrous DCM [2 mL] were added and the mixture was stirred for 2 h at rt. Then, the solution of TMSOTf in DCM [10% *v*/*v*, 60 μL, 0.2 eq.] was added at −50 °C under argon protection. The mixture was stirred under argon protection until TLC showed full consumption of the starting materials, and then neutralized with Et_3_N and the mixture was purified by chromatography (silica gel, eluent: hexane/EtOAc) to give a glycosylation product as a mixture of α- and β-isomers.

#### 3.2.3. Saponification (Standard Procedure C)

To a solution of disaccharide [0.02 mmol] in H_2_O [0.5 mL], 1 M solution of LiOH in H_2_O [0.3 mL] was added at 0 °C. The reaction mixture was kept at 0 °C until TLC showed formation of the final product and then quenched with 0.5 M AcOH and evaporated to dryness. Deprotected disaccharide was isolated from the reaction mixture by gel chromatography on a Sephadex G-15 column with water elution followed by lyophilization to give the desired product.

#### 3.2.4. Sulfation (Standard Procedure D)

To a solution of a deprotected disaccharide [0.02 mmol] in DMF [1 mL], complex Py·SO_3_ [16–20 eq., 4 eq.×-OH] was added. The reaction mixture was kept at rt overnight, and then quenched with excess of NaHCO_3_ [5 eq.×-OH] and MeOH [0.4 mL]. The mixture was stirred for 3 h and evaporated to dryness. The product was purified either by chromatography (silica gel: DCM/MeOH) or by gel chromatography on a Sephadex G-15 column to give the desired product.

### 3.3. Synthesis of Compounds ***9***, ***10***, ***11***, ***12***, ***13***, ***18***, ***19***, ***20***, ***21a***, ***21b***, ***1***, ***2***, ***3***, ***4***, ***5***, and ***6***

#### 3.3.1. (2-Methyl-5-*tert*-butylphenyl) 4,6-*O*-(4-methoxybenzylidene)-1-thio-β-d-glucopyranoside (**9**)

To a solution of glucose β-d-pentaacetate **8** [2 g, 5.1 mmol] in DCM [20 mL], MTBTP [1.32 mL, 1.4 eq.] was added. The mixture was cooled to 0 °C and BF_3_·Et_2_O [1.26 mL, 2 eq.] was added dropwise. The reaction mixture was allowed to warm to rt and stirred for 2 h. After 2 h, TLC [hexane/EtOAc = 1:1] showed full consumption of the starting material, and the reaction mixture was neutralized with Et_3_N, diluted with EtOAc [150 mL], and washed with H_2_O [2 × 100 mL]. The organic layer was dried (Na_2_SO_4_) and concentrated in vacuo. The crude thioglycoside was dissolved in MeOH [10 mL] and 1 M solution of MeONa in MeOH was added [1 mL]. After 20 min, TLC [EtOAc] showed formation of the final product and the reaction mixture was neutralized with ion exchange resin IR-120 in H^+^ form. The resin was filtered off and the filtrate was concentrated in vacuo. The crude residue was dissolved in CH_3_CN [15 mL] and CSA was added [150 mg, 10 mg/mL] followed by HC(OMe)_3_ [0.84 mL, 1.5 eq.] and 4-anisaldehyde [0.81 mL, 1.3 eq.]. After 30 min, TLC showed full consumption of the staring material, and the reaction mixture was diluted with EtOAc [150 mL] and washed with NaHCO_3_ [100 mL] and H_2_O [100 mL]. The organic layer was dried (Na_2_SO_4_) and concentrated in vacuo. The residue was purified by chromatography (silica gel, eluent: toluene/EtOA *c* = 10:1→3:1) to give **9** (1.68 g, 72%) as white crystals. R_f_ = 0.4 (toluene/EtOAc = 2:1). [α]_D_ = −41.9° (*c* = 1, EtOAc). ^1^H NMR (300 MHz, CDCl_3_): δ 7.61 (d, 1H, *J* = 2.0 Hz, SMTBP), 7.41 (d, 2H, *J* = 8.7 Hz, *o*-MPh), 7.28–7.20 (m, 1H, SMTBP), 7.15 (d, 1H, *J* = 8.0 Hz, SMTBP), 6.88 (d, 2H, *J* = 8.8 Hz, *m*-MPh), 5.48 (s, 1H, CHMPh), 4.62 (d, 1H, *J*_1–2_ = 9.8 Hz, H-1), 4.38–4.27 (m, 1H, H-6), 3.87–3.69 (m, 5H, H-3. H-6′, OCH_3_), 3.58–3.43 (m, 3H, H-2, H-4, H-5), 3.08 (br s, 1H, 3-OH), 2.84 (s, 1H, 2-OH), 2.42 (s, 3H, Me (SMTBP)), 1.31 (s, 9H, *tert-*Bu (SMTBP)). ^13^C NMR (75 MHz, CDCl_3_): δ 160.39 (SMTBP), 149.75 (*i*-MBn), 137.15 (SMTBP), 130.90 (SMTBP), 130.38 (SMTBP), 130.23 (SMTBP), 129.46 (*p*-MPh), 127.72 (*o*-MPh), 125.56 (SMTBP), 113.84 (*m*-MPh), 101.99 (MPhCH), 88.83 (C-1), 80.31 (C-4), 74.79 (C-3), 73.02 (C-2), 70.52 (C-5), 68.71 (C-6), 55.42 (OMe), 31.42 (*tert*-Bu(SMTBP)), 20.59 (Me(SMTBP)). HRMS (ESI): Calcd. *m*/*z* for [M + H]^+^ C_25_H_32_O_6_S 461.1992 found 461.1991.

#### 3.3.2. Methyl [(2-methyl-5-*tert*-butylphenyl) 2,3-di-*O*-benzyl-1-thio-β-d-glucopyranosyl] Uronate (**10**)

A solution of **9** [700 mg, 1.58 mmol] in DMF [7 mL] was cooled to 0 °C and NaH [250 mg, 4 eq.] was added portionwise. The reaction mixture was allowed to warm to rt and stirred for 30 min. Then, the mixture was cooled to 0 °C again and BnBr [0.57 mL, 3 eq.] was added dropwise. After 2 h, TLC [hexane/EtOAc = 2:1] showed formation of the final product and the reaction mixture was cooled to 0 °C and TFA [0.5 mL, 90% aq] was added. Reaction mixture was stirred at rt until TLC [hexane/EtOAc = 2:1] showed full consumption of the starting material. Then, the mixture was diluted with EtOAc [100 mL] and washed with NaHCO_3_ [100 mL] and H_2_O [100 mL]. The organic layer was dried (Na_2_SO_4_) and concentrated in vacuo. The crude residue was purified by chromatography (silica gel, eluent: hexane/EtOAc = 10:1→3:1) to give diol. The latter was dissolved in DCM [5 mL] and H_2_O [1 mL] and TEMPO [50 mg, 0.2 eq.] were added. The mixture was cooled to 0 °C and BAIB [1g, 2.5 eq.] was added portionwise. The reaction mixture was vigorously stirred for 3 h until TLC [hexane/EtOAc = 2:1] showed full consumption of the starting material. Then, the mixture was diluted with EtOAc [100 mL] and washed with Na_2_S_2_O_3_ [100 mL] and H_2_O [100 mL]. The organic layer was dried (Na_2_SO_4_) and concentrated in vacuo. The crude residue was dissolved in DMF [5 mL] and K_2_CO_3_ [200 mg, 1.5 eq.] and MeI [175 μL, 3 eq.] were added. The mixture was vigorously stirred for 3 h until TLC [hexane/EtOAc = 2:1] showed full consumption of the starting material. Then, the mixture was diluted with EtOAc [100 mL] and washed with H_2_O [2 × 100 mL]. The organic layer was dried (Na_2_SO_4_) and concentrated in vacuo. The residue was purified by chromatography (silica gel, eluent: hexane/EtOAc = 15:1→6:1) to give **10** (400 mg, 46%) as colorless oil. R_f_ = 0.55 (hexane/EtOAc = 2:1). [α]_D_ = 7.2° (*c* = 1, EtOAc). ^1^H NMR (300 MHz, CDCl_3_): δ 7.63 (d, 1H, *J* = 1.9 Hz, SMTBP), 7.43–7.13 (m, 11H, SMTBP, 2xBn), 7.08 (d, 1H, *J* = 8.0 Hz, SMTBP), 4.90 (d, 1H, *J* = 10.3 Hz, CHH’Ph), 4.84 (s, 2H, CH_2_Ph), 4.75 (d, 1H, *J* = 10.3 Hz, CHH’Ph), 4.61 (d, 1H, *J*_1–2_ = 9.4 Hz, H-1), 3.97–3.86 (m, 1H, H-4), 3.79–3.75 (m, 4H, H-5, OMe), 3.61–3.44 (m, 2H, H-2, H-3), 2.97 (br s, 1H, 4-OH), 2.35 (s, 3H, Me(SMTBP)), 1.25 (s, 9H, *tert-*Bu(SMTBP)). ^13^C NMR (75 MHz, CDCl_3_): δ 169.67 (C-6), 149.76 (Bn), 138.01 (SMTBP), 136.43 (Bn), 132.90 (SMTBP), 129.99 (SMTBP), 129.61 (SMTBP), 128.64 (Bn), 128.51 (Bn), 128.35 (Bn), 128.11 (Bn), 127.99 (Bn), 124.97 (SMTBP), 89.03 (C-1), 85.38 (C-3), 80.20 (C-2), 77.45 (C-5), 75.83 (CH_2_Ph), 75.77 (CH_2_Ph), 71.99 (C-4), 52.78 (OMe), 31.35 (*tert-*Bu(SMTBP)), 20.45 (Me(SMTBP). HRMS (ESI): Calcd. *m*/*z* for [M + Na]^+^ C_32_H_38_O_6_S 573.2281 found 573.2287.

#### 3.3.3. Methyl (2,3-di-*O*-benzyl-4-*O*-*tert*-butyldimethylsilyl-α,β-d-glucopyranosyl) Uronate Trichloroacetimidate (**11**)

NEt_3_ [65 μL, 2 eq.] and TBSOTf [80 μL, 1.5 eq.] were added under Ar protection to a solution of **10** [125 mg, 0.23 mmol] in DCM [2 mL] and the resulting mixture was stirred at rt for 2 h. After TLC showed full consumption of the starting material, the mixture was diluted with EtOAc [50 mL] and washed with H_2_O [2 × 50 mL]. The organic layer was dried (Na_2_SO_4_) and concentrated in vacuo. The crude product was treated according to the standard procedure A and after purification by chromatography (silica gel neutralized with NEt_3_, eluent: hexane/EtOAc = 30:1→10:1) gave **11** (102 mg, 69%) as a mixture of α/β-isomers = 1:1.4. R_f_ = 0.9 (hexane/EtOAc = 2:1). Analytical data for **11** were in agreement with those previously reported [25].

#### 3.3.4. Methyl (2,3-di-*O*-benzyl-4-*O*-picoloyl-α,β-d-glucopyranosyl) Uronate Trichloroacetimidate (**12**)

PicoA [47 mg, 1.5 eq.], DCC [70 mg, 1.5 eq.], and DMAP [6 mg, 0.2 eq.] were added under Ar protection to a solution of **10** [124 mg, 0.23 mmol] in DCM [2 mL] and the resulting mixture was stirred at rt for 1 h. After TLC showed full consumption of the starting material, the mixture was diluted with EtOAc [50 mL] and washed with H_2_O [2 × 50 mL]. The organic layer was dried (Na_2_SO_4_) and concentrated in vacuo. The residue was purified by chromatography (silica gel, eluent: hexane/EtOAc = 8:1→2:1) to give fully protected thioglycoside. The latter was treated according to the standard procedure A and after purification by chromatography (silica gel neutralized with NEt_3_, eluent: hexane/EtOAc = 6:1→1:1) gave **12** (80 mg, 56%) as a mixture of α/β-isomers 1:7. R_f_ = 0.3 and 0.15 (hexane/EtOAc = 2:1). ^1^H NMR (400 MHz, CDCl_3_): δ 8.75 (d, 1H, *J* = 3.8 Hz, Pico), 8.66 (s, 1H, NH), 7.99 (d, *J* = 7.8 Hz, Pico), 7.79 (td, 1H, *J* = 7.7, *J* = 1.8 Hz, Pico), 7.46 (ddd, 1, *J* = 7.6 Hz, *J* = 4.8 Hz, *J* = 1.2 Hz, Pico), 7.34–6.96 (m, 10H, 2xBn), 6.58 (d, 1H, *J*_1–2_ = 3.5 Hz, H-1 (β)), 6.55 (d, 1H, *J*_1–2_ = 2.5 Hz, H-4 (α)) 6.19 (d, 1H, *J*_4–5_ = 2.8 Hz, H-4 (α)), 5.49–5.33 (m, 1H, H-4 (β)), 4.81 (d, 1H, *J* = 11.3 Hz, CHH’Ph), 4.76–4.64 (m, 3H, CHH’Ph, CH_2_Ph), 4.59 (d, 1H, *J*_4–5_ = 10.3 Hz, H-5 (β)), 4.43 (dd, 1H, *J*_2–3_ = 7.8 Hz, *J*_3-4_ = 2.8 Hz, H-3 (α)), 4.25 (t, 1H, *J*_2–3_, *J*_3–4_ = 9.5 Hz, H-3 (β)), 3.93 (dd, *J*_2–3_ = 7.7 Hz, *J*_1–2_ = 2.5 Hz, H-2 (α)), 3.85 (dd, 1H, *J*_2–3_ = 9.5 Hz, *J*_1–2_ = 3.6 Hz, H-2 (β)), 3.59 (s, 3H, OMe). ^13^C NMR (100 MHz, CDCl_3_): δ 167.91 (C-6), 164.21 (CO(Pico)), 161.01 (CCl_3_), 147.37 (Pico), 137.99 (*i*-Bn), 137.65 (*i*-Bn), 137.14 (Pico), 128.56-127.28 (Ar), 125.74 (Pico), 111.19 (C-1 (α)), 93.90 (C-1 (β)), 78.44 (C-2 (β)), 77.65 (C-3 (β)), 75.57 (CH_2_Ph), 73.42 (CH_2_Ph), 72.04 (C-4 (β)), 70.89 (C-5 (β)), 52.98 (OMe). HRMS (ESI): Calcd. *m*/*z* for [M + Na]^+^ C_29_H_27_Cl_3_N_2_O_8_ 659.0725, found 659.0715.

#### 3.3.5. Methyl (2,3-di-*O*-benzyl-4-*O*-benzoyl-α,β-d-glucopyranosyl) Uronate Trichloroacetimidate (**13**)

NEt_3_ [100 μL, 3 eq.] and BzCl [40 μL, 1.5 eq.] were added to a solution of **10** [129 mg, 0.23 mmol] in DCM [2 mL] and the resulting mixture was stirred overnight. After TLC showed full consumption of the starting material, an excess of MeOH was added and the mixture was kept for 30 min and concentrated in vacuo. The residue was purified by chromatography (silica gel, eluent: hexane/EtOAc = 30:1→12:1) to give fully protected thioglycoside. The latter was treated according to the standard procedure A and after purification by chromatography (silica gel neutralized with NEt_3_, eluent: hexane/EtOAc = 30:1→4:1) gave **13** (77 mg, 52%) as a mixture of α/β-isomers 1:2.1. R_f_ = 0.8 (hexane/EtOAc = 2:1). ^1^H NMR (400 MHz, CDCl_3_): δ 8.73 (s, 1H, NH(β)), 8.66 (s, 1H, NH(α)), 7.97–7.87 (m, 4H, *o*-Bz(α), *o*-Bz(β)), 7.56–7.52 (m, 2H, *p*-Bz(α), *p*-Bz(β)), 7.43–7.35 (m, 4H, *m*-Bz(α), *m*-Bz(β)), 7.29–7.06 (m, 20H, 2xBn(α), 2xBn(β)), 6.55 (d, 1H, *J*_1–2_ = 3.4 Hz, H-1 (α)), 5.91 (d, 1H, *J*_1–2_ = 7.3 Hz, H-1 (β)), 5.48 (t, 1H, *J*_3–4_, *J*_4–5_ = 9.2 Hz, H-4 (β)), 5.37 (t, 1H, *J*_3–4_, *J*_4–5_ = 9.8 Hz, H-4 (α)), 4.90 (d, 1H, *J* = 10.9 Hz, CHH’Ph(β)), 4.77–4.63 (m, 7H, CHH’Ph(β), CH_2_Ph(β), 2xCH_2_Ph(α)), 4.46 (d, 1H, *J*_4–5_ = 10.3 Hz, H-5 (α)), 4.22 (d, 1H, *J*_4–5_ = 9.6 Hz, H-5 (β)), 4.14 (t, 1H, *J*_2–3_, *J*_3–4_ = 9.4 Hz, H-3 (α)), 3.94–3.84 (m, 3H, H-2 (α), H-2 (β), H-3 (β)), 3.58 (s, 3H, OMe (β)), 3.56 (s, 3H, OMe (α)). ^13^C NMR (100 MHz, CDCl_3_): δ 168.04 (C-6 (α)), 167.57 (C-6 (β)), 165.47 (COPh (α)), 165.37 (COPh (β)), 161.15 (CCl_3_ (β)), 160.85 (CCl_3_ (α)), 137.67 (*i*-Bn), 133.51 (*p*-Bz (α), *p*-Bz (β)), 129.95 (*o*-Bz(α)), 129.90 (*o*-Bz(β)), 128.60–127.81 (Ar), 97.72 (C-1 (β)), 93.86 (C-1(α)), 80.73 (C-3 (β)), 80.15 (C-2 (β)), 78.48 (C-2 (α)), 77.49 (C-3 (α)), 75.47 (CH_2_Ph (α)), 75.24 (CH_2_Ph (β)), 75.10 (CH_2_Ph (β)), 73.45 (C-5 (β)), 73.40 (CH_2_Ph (α)), 71.34 (C-4 (β)), 71.14 (C-4 (α), C-5 (α)), 52.96 (OMe (α)), 52.92 (OMe (β)). HRMS(ESI): Calcd. *m*/*z* for [M + Na]^+^ C_30_H_28_Cl_3_NO_8_ 658.0773 found 658.0769.

#### 3.3.6. Allyl 3,4-*O*-isopropylidene-2-*O*-(methyl 2,3-di-*O*-benzyl-4-*O*-*tert*-butyldimethylsilyl-α,β-d-glucopyranosyluronate)-α-L-fucopyranoside (**18**)

The glycosylation was performed according to the standard procedure B with **7** [40 mg, 0.16 mmol] and **11** [102 mg, 0.16 mmol]. Purification by chromatography (silica gel, eluent: hexane/EtOAc = 20:1→8:1) gave **18** (109 mg, 91%) as a mixture of α/β-isomers 1.2:1. R_f_ = 0.8 (hexane/EtOAc = 2:1). ^1^H NMR (600 MHz, CDCl_3_): δ 7.36–7.21 (m, 20H, 2xBn (α), 2xBn (β)), 6.01 (ddd, 1H, *J* = 22.7 Hz, *J* = 10.8 Hz, *J* = 5.6 Hz, CH_2_CH=CH_2_ (α)), 5.80 (ddd, 1H, *J* = 22.5 Hz, *J* = 10.8 Hz, *J* = 5.6 Hz, CH_2_CH=CH_2_ (β)), 5.41 (dd, 1H, *J* = 17.2, *J* = 1.6 Hz, CH_2_CH=CHH’ (α)), 5.28–5.22 (m, 3H, H-1 (GlcA, α) CH_2_CH=CHH’ (α), CH_2_CH=CHH’ (β)), 5.13–5.08 (m, 2H, CH_2_CH=CHH’ (β), CHH’Ph (α)), 5.04–5.02 (m, 2H, 2xCHH’Ph (β)), 4.97 (d, 1H, *J*_1–2_ = 3.3 Hz, H-1 (Fuc, β)), 4.96 (d, 1H, *J*_1–2_ = 3.6 Hz, H-1(Fuc, α)), 4.76 (d, 1H, *J* = 11.3 Hz, CHH’Ph (α)), 4.72–4.68 (m, 2H, CHH’Ph (α), CHH’Ph (β)), 4.63–4.61 (m, 2H, H-1 (GlcA (β), CHH’Ph (β)), 4.58 (d, 1H, *J* = 11.3 Hz, CHH’Ph (α)), 4.45 (dd, 1H, *J*_2–3_ = 8.1 Hz, *J*_3–4_ = 5.5 Hz, H-3 (Fuc, α)), 4.40 (dd, 1H, *J*_2–3_ = 7.8 Hz, *J*_3–4_ = 5.5 Hz, H-3 (Fuc, β)), 4.28 (d, 1H, *J*_4–5_ = 9.5 Hz, H-5 (GlcA, α)), 4.26–4.08 (m, 7H, H-4 (Fuc, α), H-4 (Fuc, β), H-5 (Fuc, α), H-5 (Fuc, β), OCH_2_ (α), OCHH’ (β)), 4.04–3.98 (m, 2H, H-2 (Fuc, β), H-4 (GlcA, β), 3.94 (dd, 1H, *J* = 12.9 Hz, *J* = 6.0 Hz, CHH’ (β)), 3.87 (t, 1H, *J*_4–5_ = 9.1 Hz, H-4 (GlcA, α)), 3.83–3.80 (m, 2H, H-3 (GlcA, α), H-5 (GlcA, β)), 3.77–3.72 (m, 7H, H-2 (Fuc, α), OMe (α), OMe (β)), 3.64–3.59 (m, 2H, H-2 (GlcA, α), H-2 (GlcA, β)), 3.48 (t, 1H, *J*_2–3_, *J*_3–4_ = 8.7 Hz, H-3 (Glc, β)), 1.55 (s, 3H, CMe (α)), 1.50 (s, 3H, CMe (β)), 1.40–1.34 (m, 12H, H-6 (Fuc, α), H-6 (Fuc, β), CMe (α), CMe (β)), 0.85 (s, 9H, *tert-*BuSi (α)), 0.83 (s, 9H, *tert-*BuSi (β)), 0.01 (s, 3H, SiMe (α)), −0.02 (s, 3H, SiMe (β)), −0.02 (s, 3H, SiMe (α)), −0.03 (s, 3H, SiMe (β)). ^13^C NMR (150 MHz, CDCl_3_): δ 170.08 (C-6 (GlcA, β)), 168.63 (C-6 (GlcA, α)), 139.26 (*i*-Bn (α)), 138.89 (*i*-Bn (β)), 138.48 (*i*-Bn (β)), 138.06 (*i*-Bn (α)), 133.94 (CH_2_CH=CH_2_ (α)), 133.87 (CH_2_CH=CH_2_ (β)), 128.50–127.04 (Ar), 117.72 (CH_2_CH=CH_2_ (α)), 117.38 (CH_2_CH=CH_2_ (β)), 109.05 (CMe_3_ (β)), 108.92 (CMe_3_ (α)), 102.13 (C-1 (GlcA, β)), 99.17 (C-1 (GlcA, α)), 97.59 (C-1 (Fuc, α)), 95.45 (C-1 (Fuc, β)), 84.10 (C-3 (Glc, β)), 81.67 (C-2 (GlcA, β)), 80.83 (C-3 (GlcA, α)), 79.51 (C-2 (GlcA, α)), 78.78 (C-2 (Fuc, α)), 76.58 (C-5 (Glc, β)), 76.44 (C-4 (Fuc, α)), 76.34 (C-4 (Fuc, β)), 75.38 (C-2 (Fuc, β)), 75.21 (C-3 (Fuc, α)), 75.00 (CH_2_Ph, α), 74.84 (CH_2_Ph, β), 74.27 (C-3 (Fuc, β), CH_2_Ph, β), 72.61 (C-5 (GlcA, α)), 72.27 (C-4 (GlcA, β)), 72.07 (C-4 (GlcA, β)), 71.98 (OCH_2_Ph, α), 68.77 (OCH_2_, α), 68.62 (OCH_2_, β), 64.07 (C-5 (Fuc, α)), 63.25 (C-5 (Fuc, α)), 52.30 (OMe, α), 52.16 (OMe, β), 28.65 (CMe, α), 27.82 (CMe, β), 26.62 (CMe, α), 26.43 (CMe, β), 25.86 (*tert-*BuSi, β), 25.81 (*tert-*BuSi, α), 16.41 (C-6 (Fuc, α), C-6 (Fuc, β)), −3.83 (SiMe, α), −3.93 (SiMe, β), −5.20 (SiMe, β), −5.23 (SiMe, α). HRMS (ESI): Calcd. *m*/*z* for [M + NH_4_]^+^ C_39_H_56_O_11_Si 746.3930, found 746.3933.

#### 3.3.7. Allyl 3,4-*O*-isopropylidene-2-*O*-(methyl 2,3-di-*O*-benzyl-4-*O*-picoloyl-α,β-d-glucopyranosyluronate)-α-L-fucopyranoside (**19**)

The glycosylation was performed according to the standard procedure B with **7** [30 mg, 0.12 mmol] and **12** [80 mg, 0.12 mmol]. Purification by chromatography (silica gel, eluent: hexane/EtOAc = 4:1→1:1) gave 19 (53 mg, 60%) as a mixture of α/β-isomers 7.4:1. R_f_ = 0.2 (hexane/EtOAc = 2:1). ^1^H NMR (600 MHz, CDCl_3_): δ 8.77 (d, 1H, *J* = 4.2 Hz, 1-Pico, α), 8.74 (d, 1H, *J* = 4.1 Hz, 1-Pico, β), 8.02 (d, 1H, *J* = 7.9 Hz, 4-Pico, β), 8.00 (d, 1H, *J* = 7.8 Hz, 4-Pico, α), 7.82–7.79 (m, 2H, 3-Pico (α), 3-Pico (β)), 7.50–7.44 (m, 2H, 2-Pico (α), 2-Pico (β)), 7.41–7.06 (m, 20H, 2xBn (α), 2xBn (β)), 5.93 (ddd, 1H, *J* = 22.8 Hz, *J* = 10.8 Hz, *J* = 5.7 Hz, CH_2_CH=CH_2_ (α)), 5.82 (ddd, 1H, *J* = 22.4 Hz, *J* = 10.7 Hz, *J* = 5.6 Hz, CH_2_CH=CH_2_ (β)), 5.52 (t, 1H, *J*_3–4_, *J*_4–5_ = 9.7 Hz, H-4 (GlcA, β)), 5.37 (t, 1H, *J*_3–4_, *J*_4–5_ = 9.8 Hz, H-4 (GlcA, α), 5.31 (dd, 1H, *J* = 17.2 Hz, *J* = 1.6 Hz, CH_2_CH=CHH’ (α)), 5.28–5.24 (m, 2H, H-1 (GlcA, α), CH_2_CH=CHH’ (β)), 5.16 (dd, 1H, *J* = 10.4 Hz, *J* = 1.2 Hz, CH_2_CH=CHH’ (α)), 5.12 (dd, 1H, *J* = 10.4 Hz, *J* = 1.3 Hz, CH_2_CH=CHH’ (β)), 5.03 (d, 1H, *J* = 11.4 Hz, CHH’Ph, β), 4.98 (d, 1H, *J*_1–2_ = 3.3 Hz, H-1 (Fuc, β)), 4.91 (d, 1H, *J*_1–2_ = 3.5 Hz, H-1 (Fuc, α)), 4.87 (d, 1H, *J* = 11.3 Hz, CHH’Ph, α), 4.78–4.71 (m, 6H, CHH’Ph (α), CHH’Ph (β), CH_2_Ph (α), CH_2_Ph (β)), 4.66–4.63 (m, 2H, H-1 (GlcA, β), H-5 (GlcA, α)), 4.42–4.40 (m, 2H, H-3 (Fuc, α), H-3 (Fuc, β)), 4.27 (t, 1H, *J*_2–3_, *J*_3–4_ = 9.4 Hz, H-3 (GlcA, α)), 4.18–4.08 (m, 7H, H-5 (GlcA, β), H-5 (Fuc, α), H-5 (Fuc, β), H-4 (Fuc, α), H-4 (Fuc, β), OCHH’ (α), OCHH’ (β)), 4.02 (dd, 1H, *J*_2–3_ = 7.9 Hz, *J*_1–2_ = 3.3 Hz, H-2 (Fuc, β)), 3.98–3.93 (m, 2H,OCHH’ (α), OCHH’ (β)), 3.90 (t, 1H, *J*_2–3_, *J*_3–4_ = 9.2 Hz, H-3 (GlcA, β)), 3.75 (dd, 1H, *J*_2–3_ = 8.0 Hz, *J*_1–2_ = 3.6 Hz, H-2 (Fuc, α)), 3.70–3.67 (m, 2H, H-2 (GlcA, α), H-2 (GlcA, β)), 3.63 (s, 3H, OMe, β), 3.61 (s, 3H, OMe, α), 1.55 (s, 3H, CMe, α), 1.52 (s, 3H, CMe, β), 1.38 (s, 3H, CMe’, α), 1.35–1.33 (m, 9H, H-6 (Fuc, α), H-6 (Fuc, β), CMe’ (β)). ^13^C NMR (150 MHz, CDCl_3_): δ 168.84 (C-6 (GlcA, α), 164.16 (CO (Pico, α), 150.06 (1-Pico, α), 147.70 (1-Pico, β), 138.51 (*i*-Bn), 138.19 (*i*-Bn), 137.00 (3-Pico α, β), 133.97 (CH_2_CH=CHH’, α, β), 128.48–127.06 (2xBn α, 2xBn β, 2 × 2-Pico α, β) 125.66 (4-Pico, β), 125.50 (4-Pico, α), 117.56 (CH_2_CH=CH_2_, α, β), 101.91 (C-1 (GlcA, β)), 98.96 (C-1 (GlcA, α)), 97.37 (C-1 (Fuc, α)), 95.39 (C-1 (Fuc, β)), 81.24 (C-3 (GlcA, β)), 81.10 (C-2 (GlcA, β)), 79.19 (C-2 (Fuc, α)), 78.66 (C-2 (GlcA, α)), 78.43 (C-3 (GlcA, α)), 76.36 (C-4 (Fuc, α)), 76.28 (C-4 (Fuc, β)), 75.95 (C-2 (Fuc, β)), 75.58 (CH_2_Ph, α), 75.39 (CH_2_Ph, β), 74.98 (C-3 (Fuc, α)), 74.69 (C-3 (Fuc, β), 74.28 (CH_2_Ph, β)), 72.71 (C-4 (GlcA, α), CH_2_Ph, α), 72.64 (C-5 (GlcA, β)), 72.26 (C-4 (GlcA, β)), 68.98 (C-5 (GlcA, α)), 68.75 (OCH_2_, α), 68.64 (OCH_2_, β), 64.08 (C-5 (Fuc, β)), 63.37 (C-5 (Fuc, α)), 52.68 (OMe, α, β), 28.60 (CMe, α), 28.00 (CMe, β), 26.52 (CMe’, α), 26.40 (CMe’, β), 16.41 (C-6 (Fuc, β), 16.35 (C-6 (Fuc, α). HRMS (ESI): Calcd. *m*/*z* for [M + Na]^+^ C_39_H_45_NO_12_ 742.2834 found 742.2839.

#### 3.3.8. Allyl 3,4-*O*-isopropylidene-2-*O*-(methyl 2,3-di-*O*-benzyl-4-*O*-benzoyl-α,β-d-glucopyranosyluronate)-α-L-fucopyranoside (**20**)

The glycosylation was performed according to the standard procedure B with **7** [30 mg, 0.12 mmol] and **13** [77 mg, 0.12 mmol]. Purification by chromatography (silica gel, eluent: toluene/EtOAc = 20:1→10:1) gave **20** (84 mg, 95%) as a mixture of α/β-isomers 1:2. R_f_ = 0.7 (hexane/EtOAc = 2:1). ^1^H NMR (600 MHz, CDCl_3_): δ 7.97 (d, 2H, *J* = 7.1 Hz, *o*-Bz, α), 7.94 (d, 2H, *J* = 7.1 Hz, *o*-Bz, β), 7.60–7.54 (m, 2H, *p*-Bz, α, β), 7.46–7.39 (m, 8H, *m*-Bz, α, β, Bn, β), 7.36–7.26 (m, 13H, Bn, α, β), 7.18–7.07 (m, 15H, Bn, α, β), 5.93 (ddd, 1H, *J* = 22.7 Hz, *J* = 10.8 Hz, *J* = 5.6 Hz, CH_2_CH=CH_2_, α), 5.81 (ddd, 1H, *J* = 22.2 Hz, *J* = 10.8, *J* = 5.6 Hz, CH_2_CH=CH_2_, β), 5.44 (t, 1H, *J*_3–4_, *J*_4–5_ = 9.6 Hz, H-4 (GlcA, β)), 5.36–5.25 (m, 3H, H-4 (GlcA, α), CH_2_CH=CHH’, α, CH_2_CH=CHH’, β), 5.22 (d, 1H, *J*_1–2_ = 3.7 Hz, H-1 (GlcA, α)), 5.18 (dd, 1H, *J* = 10.4 Hz, *J* = 1.4 Hz, CH_2_CH=CHH’, α), 5.13 (dd, 1H, *J* = 10.4 Hz, *J* = 1.4 Hz, CH_2_CH=CHH’, β), 5.05 (d, 1H, *J* = 11.3 Hz, CHH’Ph, β), 4.99 (d, 1H, *J*_1–2_ = 3.3 Hz, H-1 (Fuc, β)), 4.92 (d, 1H, *J*_1–2_ = 3.6 Hz, H-1 (Fuc, α)), 4.88 (d, 1H, *J* = 11.2 Hz, CHH’, α), 4.79–4.73 (m, 4H, CH_2_Ph, α, CHH’Ph, β, CHH’Ph, β), 4.69 (d, 1H, *J* = 11.2 Hz, CHH’Ph, α), 4.66–4.63 (m, 2H, H-1 (GlcA, β), CHH’Ph, β), 4.51 (d, 1H, *J*_4–5_ = 10.2 Hz, H-5 (GlcA, α)), 4.45–4.42 (m, 2H, H-3 (Fuc, α), H-3 (Fuc, β)), 4.22–4.12 (m, 5H, H-3 (GlcA, α), H-5 (Fuc, α), H-5 (Fuc, β), OCHH’, α, OCHH’, β), 4.12–4.09 (m, 2H, H-4 (Fuc, α), H-4 (Fuc, β)), 4.03–3.99 (m, 2H, H-2 (Fuc, β), H-5 (GlcA, β)), 3.98–3.91 (m, 2H, OCHH’, α, OCHH’, β), 3.81 (t, 1H, *J*_2–3_, *J*_3–4_ = 9.2 Hz, H-3 (GlcA, β)), 3.74 (dd, 1H, *J*_2–3_ = 8.0 Hz, *J*_1–2_ = 3.6 Hz, H-2 (Fuc, α)), 3.71–3.68 (m, 2H, H-2 (GlcA, α), H-2 (GlcA, β)), 3.62 (s, 3H, OMe, β), 3.58 (s, 3H, OMe, α), 1.57 (s, 3H, CMe, α), 1.52 (s, 3H, CMe, β), 1.41 (s, 3H, CMe’, α), 1.37–1.33 (m, 9H, H-6 (Fuc, α, H-6 (Fuc, β, CMe’, β). ^13^C NMR (150 MHz, CDCl_3_): δ 168.93 (C-6 (GlcA, α)), 167.66 (C-6 (GlcA, β)), 165.51 (CO (Bz, α)), 165.34 (CO, (Bz, β)), 138.35 (*i*-Bn), 137.83 (*i*-Bn), 133.76 (CH_2_CH=CH_2_, β), 133.72 (CH_2_CH=CH_2_, α), 133.37 (*p*-Bz, α, β), 129.84 (*o*-Bz, β), 129.82 (*o*-Bz, α), 128.54–127.65 (*m*-Bz, α, β, Bn, α, β), 117.64 (CH_2_CH=CH_2_, α), 117.51 (CH_2_CH=CH_2_, β), 109.16 (CMe_2_, α, β), 101.79 (C-1 (GlcA, β)), 99.05 (C-1 (GlcA, α)), 97.19 (C-1 (Fuc, α)), 95.30 (C-1 (Fuc, β)), 81.03 (C-2 (GlcA, β)), 80.95 (C-3 (GlcA, β)), 79.17 (C-2 (Fuc, α)), 78.53 (C-2 (GlcA, α)), 78.38 (C-3 (GlcA, α)), 76.30 (C-4 (Fuc, α)), 76.24 (C-4 (Fuc, β)), 75.95 (C-2 (Fuc, β)), 75.22 (CH_2_Ph, β), 74.84 (CH_2_Ph, α), 74.70 (CH_2_Ph, β), 74.18 (C-3 (Fuc, α), C-3 (Fuc, β)), 72.87 (CH_2_Ph, α), 72.82 (C-5 (GlcA, β)), 71.79 (C-4 (GlcA, α)), 71.48 (C-4 (GlcA, β)), 69.03 (C-5 (GlcA, α)), 68.58 (OCH_2_, β), 68.56 (OCH_2_, α), 63.98 (C-5 (Fuc, β)), 63.28 (C-5 (Fuc, α)), 52.73 (OMe, α), 52.65 (OMe, β), 28.63 (CMe, α), 28.01 (CMe, β), 26.54 (CMe’, α), 26.42 (CMe’, β), 16.41 (C-6 (Fuc, β)), 16.36 (C-6 (Fuc, α)). HRMS (ESI): Calcd. *m*/*z* for [M + NH_4_]^+^ C_40_H_46_O_12_ 736.3328 found 736.3326.

#### 3.3.9. Propyl 2-*O*-(methyl 4-*O*-benzoyl-α-d-glucopyranosyluronate)-α-L-fucopyranoside (**21a**) and propyl 2-*O*-(methyl 4-*O*-benzoyl-β-d-glucopyranosyluronate)-α-L-fucopyranoside (**21b**)

TFA [150 μL, 90% aq.] was added to a solution of **20** [84 mg, 0.11 mmol] in DCM [1.5 mL]. The reaction mixture was kept at 40 °C for 30 min. After TLC [hexane/EtOAc = 1:1] showed full consumption of the starting material, the mixture was neutralized with NEt_3_ and concentrated in vacuo. The residue was purified by chromatography (silica gel, eluent: hexane/EtOAc = 3:1→1:2) to give diol. A mixture of the resulting product and the catalyst 10% Pd/C [25 mg] in EtOAc–MeOH (1:1) [2 mL] was stirred under H_2_ at rt for 2 h. After TLC (toluene:EtOAc = 1:1) showed formation of the final product, the reaction mixture was filtered through a nylon membrane syringe filter (0.45 μm). The filtrate was concentrated in vacuo and the residue was purified by chromatography (silica gel, eluent: DCM/MeOH = 20:1→10:1) to give **21a** (17 mg, 29%) and **21b** (30 mg, 51%). R_f_ = 0.25 and 0.1 (toluene/EtOAc = 1:1).

For **21a**: [α]_D_ = −98.5° (*c* = 1, MeOH). ^1^H NMR (600 MHz, CD_3_OD_3_): 8.05 (d, 2H, *J* = 7.1 Hz, *o*-Bz)), 7.66 (t, 1H, *J* = 7.4 Hz, *p*-Bz), 7.52 (t, 2H, *J* = 7.8 Hz, *m*-Bz), 5.13–5.12 (m, 2H, H-1 (GlcA), H-4 (GlcA)), 4.91 (d, 1H, *J*_1–2_ = 3.7 Hz, H-1 (Fuc)), 4.66 (d, 1H, *J*_4–5_ = 10.3 Hz, H-5 (GlcA)), 4.07 (t, 1H, *J*_2–3_, *J*_3–4_= 9.5 Hz, H-3 (GlcA)), 4.02 (dd, 1H, *J*_2–3_ = 10.0 Hz, *J*_3–4_ = 3.3 Hz, H-3 (Fuc)), 3.95 (q, 1H, *J*_5–6_ = 6.5 Hz, H-5 (Fuc)), 3.81 (dd, 1H, *J*_2–3_ = 10.0 Hz, *J*_1–2_ = 3.7 Hz, H-2 (Fuc)), 3.75 (d, 1H, *J*_3–4_ = 3.4 Hz, H-4 (Fuc)), 3.67–3.60 (m, 2H, H-2 (GlcA), OCHH’), 3.22 (dt, 1H, *J* = 9.3 Hz, *J* = 6.6 Hz, OCHH’), 1.66 (dq, 2H, *J* = 14.2 Hz, *J* = 7.1 Hz, OCH_2_CH_2_CH_3_), 1.23 (d, 3H, *J*_5–6_ = 6.6 Hz, H-6 (Fuc)), 0.99 (t, 3H, *J* = 7.4 Hz, OCH_2_CH_2_CH_3_). ^13^C NMR (150 MHz, CD_3_OD): δ 13C NMR (151 MHz, MeOD) δ 170.84 (C-6 (GlcA)), 167.20 (COPh), 134.53 (*p*-Bz), 130.66 (*o*-Bz), 129.59 (*m*-Bz), 103.26 (C-1 (GlcA)), 99.35 (C-1 (Fuc)), 81.39 (C-2 (Fuc)), 74.02 (C-4 (GlcA)), 73.43 (C-2 (GlcA), C-4 (Fuc)), 72.14 (C-3 (GlcA)), 70.39 (C-5 (GlcA)), 70.10 (C-3 (Fuc), OCH_2_), 67.19 (C-5 (Fuc)), 53.04 (OMe), 23.87 (OCH_2_CH_2_CH_3_), 16.50 (C-6 (Fuc)), 11.25 (OCH_2_CH_2_CH_3_). HRMS (ESI): Calcd. *m*/*z* for [M + Na]^+^ C_23_H_32_O_12_ 523.1786 found 523.1786.

For **21b**: [α]_D_ = −17.6° (*c* = 1, MeOH). ^1^H NMR (600 MHz, CD_3_OD_3_): δ 8.07 (d, 2H, *J* = 7.9 Hz, *o*-Bz), 7.65 (t, 1H, *J* = 7.4 Hz, *p*-Bz), 7.51 (t, 2H, *J* = 7.8 Hz, *m*-Bz), 5.12 (t, 1H, *J*_3–4_, *J*_4–5_ = 9.7 Hz, H-4 (GlcA)), 4.98 (d, 1H, *J*_1–2_ = 3.7 Hz, H-1 (Fuc)), 4.66 (d, 1H, *J*_1–2_ = 7.9 Hz, H-1 (GlcA)), 4.30 (d, 1H, *J*_4–5_ = 10.0 Hz, H-5 (GlcA)), 4.04 (dd, 1H, *J*_2–3_ = 10.2 Hz, *J*_1–2_ = 3.7 Hz, H-2 (Fuc)), 4.00 (q, 1H, *J*_5–6_ = 6.6 Hz, H-5 (Fuc)), 3.94 (dd, 1H, *J*_2–3_ = 10.2 Hz, *J*_3–4_ = 3.3 Hz, H-3 (Fuc)), 3.87 (t, 1H, *J*_2–3_, *J*_3–4_ = 9.3 Hz, H-3 (GlcA)), 3.76 (d, 1H, *J*_3–4_ = 3.3 Hz, H-4 (Fuc)), 3.66–3.60 (m, 1H, OCHH’), 3.57 (s, 3H, OMe), 3.54–3.50 (m, 1H, H-2 (GlcA)), 3.49–3.46 (m, 1H, OCHH’), 1.71–1.63 (m,2H, CH_2_CH_2_CH_3_), 1.25 (d, 3H, *J*_5–6_ = 5.6 Hz, H-6 (Fuc)), 0.97 (t, 3H, *J* = 7.4 Hz, CH_2_CH_2_CH_3_). ^13^C NMR (150 MHz, CD_3_OD): δ 170.09 (C-6 (GlcA)), 167.32 (COPh), 134.46 (*p*-Bz), 130.69 (*o*-Bz), 129.52 (*m*-Bz), 102.78 (C-1 (GlcA)), 98.24 (C-1 (Fuc)), 77.11 (C-2 (Fuc)), 74.71 (C-3 (GlcA)), 74.08 (C-2 (GlcA)), 73.64 (C-4 (GlcA)), 73.39 (C-5 (GlcA)), 73.14 (C-4 (Fuc)), 70.85 (OCH_2_), 69.74 (C-3 (Fuc)), 67.22 (C-5 (Fuc)), 53.10 (OMe), 23.70 (CH_2_CH_2_CH_3_), 16.53 (C-6 (Fuc)), 11.03 (CH_2_CH_2_CH_3_). HRMS (ESI): Calcd. *m*/*z* for [M + Na]^+^ C_23_H_32_O_12_ 523.1786 found 523.1786.

#### 3.3.10. Propyl 2-*O*-(α-d-glucopyranosyluronic acid)-α-L-fucopyranoside Sodium Salt (**1**)

Final deprotection of **21a** [11.5 mg, 0.024 eq.] was performed according to the standard procedure C. Purification by gel chromatography followed by lyophilization gave **1** (7 mg, 74%). Analytical data for **1** were in agreement with those previously reported [36]. [α]_D_ = −21° (*c* = 1, H_2_O). ^1^H NMR (600 MHz, D_2_O): δ 5.10 (d, 1H, *J*_1–2_ = 3.9 Hz, H-1 (GlcA)), 5.02 (d, 1H, *J*_1–2_ = 3.8 Hz, H-1 (Fuc)), 4.07 (q, 1H, *J*_5–6_ = 6.5 Hz, H-5 (Fuc)), 4.03–3.98 (m, 2H, H-5 (GlcA), H-3 (Fuc)), 3.84 (d, 1H, *J*_3–4_ = 3.0 Hz, H-4 (Fuc)), 3.78 (dd, 1H, *J*_2–3_ = 10.3 Hz, *J*_1–2_ = 3.8 Hz, H-2 (Fuc)), 3.74 (t, 1H, *J*_2–3_, *J*_3–4_ = 9.5 Hz, H-3 (GlcA)), 3.65–3.60 (m, 1H, OCHH’), 3.58 (dd, 1H, *J*_2–3_ = 10.0 Hz, *J*_1–2_ = 3.9 Hz, H-2 (GlcA)), 3.48 (t, 1H, *J*_3–4_, *J*_4–5_ = 9.6 Hz, H-4 (GlcA)), 3.43–3.39 (m, 1H, OCHH’), 1.66–1.58 (m, 2H, CH_2_CH_2_CH_3_), 1.21 (d, 3H, *J*_5–6_ = 6.6 Hz, H-6 (Fuc)), 0.91 (t, *J* = 7.4 Hz, CH_2_CH_2_CH_3_). ^13^C NMR (150 MHz, D_2_O): δ 101.66 (C-1 (GlcA)), 98.39 (C-1 (Fuc)), 78.64 (C-2 (Fuc)), 73.27 (C-3 (GlcA)), 72.42 (C-4 (Fuc)), 72.37 (C-4 (GlcA)), 72.24 (C-5 (GlcA)), 72.02 (C-2 (GlcA)), 70.76 (OCH_2_), 69.02 (C-3 (Fuc)), 66.80 (C-5 (Fuc)), 22.66 (CH_2_CH_2_CH_3_), 15.68 (C-6 (Fuc)), 10.51 (CH_2_CH_2_CH_3_). HRMS (ESI): Calcd. *m*/*z* for [M − H]^−^ C_15_H_26_O_11_ 381.1402 found 381.1393.

#### 3.3.11. Propyl 2-*O*-(2,3-di-*O*-sulfo-α-d-glucopyranosyluronic acid)-3,4-di-*O*-sulfo-α-L-fucopyranoside Sodium Salt (**2**)

According to the standard procedure D, **21a** [5.5 mg, 0.011 eq.] was treated followed by chromatography (silica gel, eluent: DCM/MeOH = 5:1→2:1). Product of sulfation was treated according to standard procedure C. Purification by gel chromatography followed by lyophilization gave **2** (3.5 mg, 64%). [α]_D_ =−44° (*c* = 1, H_2_O). ^1^H NMR (600 MHz, D_2_O): δ 5.39 (d, 1H, *J*_1–2_ = 3.7 Hz, H-1 (GlcA)), 5.06 (d, 1H, *J*_1–2_ = 3.7 Hz, H-1 (Fuc)), 4.97 (d, 1H, *J*_3–4_ = 2.9 Hz, H-4 (Fuc)), 4.72 (dd, 1H, *J*_2–3_ = 10.6 Hz, *J*_3–4_ = 3.0 Hz, H-3 (Fuc)), 4.62 (t, 1H, *J*_2–3_, *J*_3–4_ = 9.5 Hz, H-3 (GlcA)), 4.29 (dd, 1H, *J*_2–3_ = 10.0 Hz, *J*_1–2_ = 3.8 Hz, H-2 (GlcA)), 4.22 (q, 1H, *J*_5–6_ = 6.5 Hz, H-5 (Fuc)), 4.11 (d, 1H, *J*_4–5_ = 10.2 Hz, H-5 (GlcA)), 4.01 (dd, 1H, *J*_2–3_ = 10.6 Hz, *J*_1–2_ = 3.7 Hz, H-2 (Fuc)), 3.71 (dd, 1H, *J*_4–5_ = 10.0 Hz, *J*_3–4_ = 9.0 Hz, H-4 (GlcA)), 3.66–3.61 (m, 1H, OCHH’), 3.47 (dt, 1H, *J* = 9.5 Hz, *J* = 6.6 Hz, OCHH’), 1.70–1.61 (m, 2H, CH_2_CH_2_CH_3_), 1.27 (d, 3H, *J*_5–6_ = 6.5 Hz, H-6 (Fuc)), 0.93 (t, 3H, *J* = 7.4 Hz, CH_2_CH_2_CH_3_). ^13^C NMR (150 MHz, D_2_O): δ 176.27 (C-6 (GlcA)), 99.35 (C-1 (GlcA)), 98.29 (C-1 (Fuc)), 79.74 (C-4 (Fuc)), 78.87 (C-3 (GlcA)), 76.18 (C-2 (Fuc)), 75.26 (C-2 (GlcA)), 73.94 (C-3 (Fuc)), 72.78 (C-5 (GlcA)), 71.79 (C-4 (GlcA)), 66.41 (C-5 (Fuc)), 22.80 (CH_2_CH_2_CH_3_), 16.36 (C-6 (Fuc)), 10.64 (CH_2_CH_2_CH_3_). HRMS (ESI): Calcd. *m*/*z* for [M − 2Na + H]^−^ C_15_H_21_Na_5_O_23_S_4_ 766.9133 found 766.9118.

#### 3.3.12. Propyl 2-*O*-(2,3,4-tri-*O*-sulfo-α-d-glucopyranosyluronic acid)-3,4-di-*O*-sulfo-α-L-fucopyranoside Sodium Salt (**3**)

Exhaustive sulfation of **1** [3.2 mg, 0.008 eq.] was performed according to the standard procedure D. Purification by gel chromatography followed by lyophilization gave **3** (5 mg, 72%). Analytical data for **3** were in agreement with those previously reported [37]. [α]_D_ = −13° (*c* = 1, H_2_O). ^1^H NMR (600 MHz, D_2_O): δ 5.44 (d, 1H, *J*_1–2_ = 2.6 Hz, H-1 (GlcA)), 5.07 (d, 1H, *J*_1–2_ = 3.8 Hz, H-1 (Fuc)), 4.95–4.93 (m, 2H, H-3 (GlcA), H-4 (Fuc)), 4.76–4.71 (m, 2H, H-3 (Fuc), H-4 (GlcA)), 4.52 (dd, 1H, *J*_2–3_ = 6.4 Hz, *J*_1–2_ = 2.6 Hz, H-2 (GlcA)), 4.39 (d, 1H, *J*_4–5_ = 5.4 Hz, H-5 (GlcA)), 4.24–4.19 (m, 2H, H-2 (Fuc), H-5 (Fuc)), 3.66–3.57 (m, 2H, OCH_2_), 1.73–1.61 (m, 2H, CH_2_CH_2_CH_3_), 1.28 (d, 3H, *J*_5–6_ = 6.6 Hz, H-6 (Fuc)), 0.93 (t, 3H, *J* = 7.4 Hz, CH_2_CH_2_CH_3_). ^13^C NMR (150 MHz, D_2_O): δ 98.53 (C-1 (Fuc)), 96.94 (C-1 (GlcA)), 79.75 (C-4 (Fuc)), 75.61 (C-5 (GlcA)), 74.86 (C-2 (Fuc)), 74.81 (C-4 (GlcA)), 74.55 (C-3 (GlcA)), 74.44 (C-3 (Fuc)), 73.35 (C-2 (GlcA)), 71.13 (OCH_2_), 66.42 (C-5 (Fuc)), 22.67 (CH_2_CH_2_CH_3_), 16.39 (C-6 (Fuc)), 10.56 (CH_2_CH_2_CH_3_). HRMS (ESI): Calcd. *m*/*z* for [M − 2Na + H]^−^ C_15_H_20_Na_6_O_26_S_5_ 868.8515 found 868.8501.

#### 3.3.13. Propyl 2-*O*-(β-d-glucopyranosyluronic acid)-α-L-fucopyranoside Sodium Salt (**4**)

Final deprotection of **21b** [8.5 mg, 0.016 eq.] was performed according to the standard procedure C. Purification by gel chromatography followed by lyophilization gave **4** (4.8 mg, 70%). [α]_D_ = −83° (*c* = 1, H_2_O). ^1^H NMR (600 MHz, D_2_O): δ 5.05 (d, 1H, *J*_1–2_ = 3.4 Hz, H-1 (Fuc)), 4.56 (d, 1H, *J*_1–2_ = 7.9 Hz, H-1 (GlcA)), 4.09 (q, 1H, *J*_5–6_ = 6.6 Hz, H-5 (Fuc)), 4.00 (dd, 1H, *J*_2–3_ = 10.4 Hz, *J*_1–2_ = 3.6 Hz, H-2 (Fuc)), 3.95 (dd, 1H, *J*_2–3_ = 10.3 Hz, *J*_1–2_ = 3.2 Hz, H-3 (Fuc)), 3.84 (d, 1H, *J*_3–4_ = 2.9 Hz, H-4 (Fuc)), 3.76–3.71 (m, 1H, H-5 (GlcA)), 3.63 (dt, 1H, *J* = 14.4 Hz, *J* = 7.2 Hz, OCHH’), 3.54–3.48 (m, 3H, H-3 (GlcA), H-4 (GlcA), OCHH’), 3.36 (t, 1H, *J*_1–2_, *J*_2–3_ = 8.0 Hz, H-2 (GlcA)), 1.62 (dd, 2H, *J* = 14.3 Hz, *J* = 7.1 Hz, CH_2_CH_2_CH_3_), 1.22 (d, 3H, *J*_5–6_ = 6.6 Hz, H-6 (Fuc)), 0.91 (t, 3H, *J* = 7.4 Hz, CH_2_CH_2_CH_3_). ^13^C NMR (150 MHz, D_2_O): δ 101.56 (C-1 (GlcA)), 96.73 (C-1 (Fuc)), 76.38 (C-5 (GlcA)), 76.14 (C-2 (Fuc)), 76.08 (C-3 (GlcA)), 73.39 (C-2 (GlcA)), 72.32 (C-4 (GlcA)), 72.25 (C-4 (Fuc)), 70.72 (OCH_2_), 68.76 (C-3 (Fuc)), 66.96 (C-5 (Fuc)), 22.59 (CH_2_CH_2_CH_3_), 15.78 (C-6 (Fuc)), 10.49 (CH_2_CH_2_CH_3_). HRMS (ESI): Calcd. *m*/*z* for [M − H]^−^ C_15_H_26_O_11_ 381.1402 found 381.1405.

#### 3.3.14. Propyl 2-*O*-(2,3-di-*O*-sulfo-β-d-glucopyranosyluronic acid)-3,4-di-*O*-sulfo- α-L-fucopyranoside Sodium Salt (**5**)

According to the standard procedure D, **21b** [10.5 mg, 0.021 eq.] was treated followed by chromatography (silica gel, eluent: DCM/MeOH = 4:1→1:1). Product of sulfation was treated according to standard procedure C. Purification by gel chromatography followed by lyophilization gave **5** (6 mg, 57%). [α]_D_ = −23° (*c* = 1, H_2_O). ^1^H NMR (600 MHz, D_2_O): δ 5.23 (d, 1H, *J*_1–2_ = 3.7 Hz, H-1 (Fuc)), 4.95 (d, 1H, *J*_3–4_ = 2.6 Hz, H-4 (Fuc)), 4.92 (d, 1H, *J*_1–2_ = 7.1 Hz, H-1 (GlcA)), 4.75 (dd, 1H, *J*_2–3_ = 6.3 Hz, *J*_3–4_ = 2.5 Hz, H-3 (Fuc)), 4.48 (t, 1H, *J*_2–3_, *J*_3–4_ = 7.8 Hz, H-3 (GlcA)), 4.28 (t, 1H, *J*_1–2_, *J*_2–3_ = 7.3 Hz, H-2 (Fuc)), 4.24 (q, 1H, *J*_5–6_ = 6.4 Hz, H-5 (Fuc)), 4.16 (dd, 1H, *J*_2–3_ = 10.5 Hz, *J*_1–2_ = 3.7 Hz, H-2 (Fuc)), 3.94–3.88 (m, 2H, H-4 (GlcA), H-5 (GlcA)), 3.64–3.57 (m, 2H, OCH_2_), 1.64 (dq, 2H, *J* = 14.5 Hz, *J* = 7.3 Hz, CH_2_CH_2_CH_3_), 1.27 (d, 3H, *J*_5–6_ = 6.6 Hz, H-6 (Fuc)), 0.91 (t, 3H, *J* = 7.4 Hz, CH_2_CH_2_CH_3_). ^13^C NMR (150 MHz, D_2_O): δ 101.41 (C-1 (GlcA)), 97.24 (C-1 (Fuc)), 81.63 (C-3 (GlcA)), 79.70 (C-4 (Fuc)), 77.99 (C-2 (GlcA)), 77.12 (C-5 (GlcA)), 75.44 (C-2 (Fuc)), 74.86 (C-3 (Fuc)), 71.33 (C-4 (GlcA)), 71.11 (OCH_2_), 66.52 (C-5 (Fuc)), 22.67 (CH_2_CH_2_CH_3_), 16.33 (C-6 (Fuc)), 10.48 (CH_2_CH_2_CH_3_). HRMS (ESI): Calcd. *m*/*z* for [M − 3Na + 2H]^−^ C_15_H_21_Na_5_O_23_S_4_ 744.9314 found 744.9217.

#### 3.3.15. Propyl 2-*O*-(2,3,4-tri-*O*-sulfo-β-d-glucopyranosyluronic acid)-3,4-di-*O*-sulfo- α-L-fucopyranoside Sodium Salt (**6**)

Exhaustive sulfation of **4** [3.8 mg, 0.09 eq.] was performed according to the standard procedure D. Purification by gel chromatography followed by lyophilization gave **6** (6.8 mg, 78%). [α]_D_ = −47° (*c* = 1, H_2_O). ^1^H NMR (400 MHz, D_2_O): δ 5.21 (dt, 1H, *J*_3–4_ = 4.1 Hz, *J*_4–5_ = 1.3 Hz, H-4 (GlcA)), 5.17 (d, 1H, *J*_1–2_ = 3.7 Hz, H-1 (Fuc)), 5.08 (dd, 1H, *J*_3–4_ = 4.1 Hz, *J*_2–3_ = 1.2 Hz, H-3 (GlcA)), 4.98 (d, 1H, *J*_1–2_ = 6.8 Hz, H-1 (GlcA)), 4.94 (d, 1H, *J*_3–4_ = 3.0 Hz, H-4 (Fuc)), 4.74 (dd, 1H, *J*_2–3_ = 10.6 Hz, *J*_3–4_ = 3.0 Hz, H-3 (Fuc)), 4.62 (d, 1H, *J*_4–5_ = 1.6 Hz, H-5 (GlcA)), 4.48 (d, 1H, *J*_1–2_ = 6.7 Hz, H-2 (GlcA)), 4.25 (q, 1H, *J*_5–6_ = 6.5 Hz, H-5 (Fuc)), 4.16 (dd, 1H, *J*_2–3_ = 10.6 Hz, *J*_2–3_ = 3.7 Hz, H-2 (Fuc)), 3.62 (t, 2H, *J* = 6.9 Hz, OCH_2_), 1.65 (h, 2H, *J* = 7.2 Hz, CH_2_CH_2_CH_3_), 1.27 (d, 3H, *J*_5–6_ = 6.6 Hz, H-6 (Fuc)), 0.91 (t, 3H, *J* = 7.4 Hz, CH_2_CH_2_CH_3_). ^13^C NMR (100 MHz, D_2_O): δ 99.91 (C-1 (GlcA)), 97.02 (C-1 (Fuc)), 79.70 (C-4 (Fuc)), 78.90 (C-5 (GlcA)), 78.60 (C-2 (GlcA)), 75.89 (C-3 (GlcA)), 75.28 (C-4 (GlcA)), 74.83 (C-2 (Fuc)), 74.33 (C-3 (Fuc)), 71.42 (OCH_2_), 66.56 (C-5 (Fuc)), 22.69 (CH_2_CH_2_CH_3_), 16.37 (C-6 (Fuc)), 10.51 (CH_2_CH_2_CH_3_). HRMS (ESI): Calcd. *m*/*z* for [M − 3Na + 2H]^−^ C_15_H_20_Na_6_O_26_S_5_ 846.8701 found 846.8489.

All spectra data obtained are presented in Supplementary Materials.

## 4. Conclusions

Model glycosylations clearly indicate that the picoloyl-assisted HAD method is a prospective approach to α-directing stereocontrol of glycosylation with certain GlcA donors. The α:β ratio when employing HAD was 7.4:1 compared to 1.2:1 and 1:2 for non-HAD-mediated cases. The stereoselectivity for larger oligosaccharides and more hindered acceptors could be even higher.

Six disaccharide models were synthesized and studied by means of NMR and DFT methods of different levels. Analysis of ^1^H chemical shifts along with the intra-ring coupling constants in the case of the β-linked saccharides suggested conformational changes occurring in the glucuronic acid pyranoside ring upon the exhaustive sulfation. Calculations of NMR shielding constants by means of the DLPNO-MP2 approach confirmed that the observed changes in chemical shifts corresponded to the change from ^4^*C*_1_ to ^3^*S*_1_ and ^1^*S*_5_ conformation. Analysis of NOE spectra for fully sulfated and non-sulfated β-disaccharides provided additional support for this result. In substances where glucuronic acid residue had α-configuration, no changes occurred. Absence of the sulfate group at *O*-4 in the β-glucuronic acid residue resulted in a less pronounced but similar effect.

## Figures and Tables

**Table 1:** ^1^H chemical shifts (ppm) observed for compounds **1**–**6**.

Structure	Residue	H-1	H-2	H-3	H-4	H-5
**1**	GlcA	5.10	3.58	3.74	3.48	4.05
	Fuc	5.02	3.78	4.05	3.84	4.07
**2**	GlcA	5.40	4.29	4.62	3.72	4.11
	Fuc	5.06	4.01	4.73	4.97	4.22
**3**	GlcA	5.44	4.52	4.94	4.74	4.39
	Fuc	5.07	4.21	4.74	4.94	4.21
**4**	GlcA	4.55	3.35	3.50	3.50	3.72
	Fuc	5.05	4.00	3.94	3.84	4.09
**5**	GlcA	4.92	4.28	4.48	3.87–3.94	3.87–3.94
	Fuc	5.23	4.16	4.74–4.79	4.95	4.24
**6**	GlcA	4.98	4.48	5.08	5.21	4.62
	Fuc	5.17	4.16	4.73	4.94	4.25

**Table 2:** Intra-ring coupling constants (Hz) and peak multiplicity in the glucuronic acid residues of compounds **1**–**6**.

Structure	H-1	H-2	H-3	H-4	H-5
**1**	3.9, d	3.9, 10.0, dd	9.5, t	9.6, t	m
**2**	3.7, d	3.8, 10.0, dd	9.5, t	9.0, 10.0, dd	10.2, d
**3**	2.6, d	2.6, 6.4, dd	m	m	5.4, d
**4**	7.9, d	m	m	m	9.5, d
**5**	7.1, d	7.3, t	7.8, t	m	m
**6**	6.8, d	6.7, d	1.2, 4.1, dd	1.3, 4.2, dt	1.6, d

**Table 3:** Relative energies for sulfated structures **2**, **3**, **5**, and **6** obtained after M062X/def2-tzvp reoptimization.

Structure	2	3	5	6
Conformer	Energy, Relative to ^4^*C*_1_, kcal/mole			
**^4^ *C*_1_**	0	0	0	0
**^1^ *S*_5_**	6.1	4.0	−1.7	−1.2
**^3^ *S*_1_**	6.7	3.6	2.9	−3.0
**^O^ *S*_2_**	6.9	3.2	0.8	0.4

**Table 4:** Chemical shielding constants (ppm) calculated for different conformers of molecule **6**.

Conformer	H-1	H-2	H-3	H-4	H-5
**^O^ *S*_2_**	26.5	26.6	26.7	27.4	27.7
**^3^ *S*_1_**	27.5	27.5	26.7	26.3	27.3
**^4^ *C*_1_**	27.4	27.4	27.7	27.7	27.5
**^1^ *S*_5_**	26.0	27.0	27.5	26.6	27.9

**Table 5:** Intra-ring ^1^H-^1^H spin–spin coupling constants (*J*, Hz) calculated for different conformers of molecule **6**.

Conformer	H-1/H-2	H-2/H-3	H-3/H-4	H-4/H-5
**^O^ *S*_2_**	0.2	3.6	0.5	9.9
**^3^ *S*_1_**	7.8	0.4	4.4	0.8
**^4^ *C*_1_**	8.0	10.9	10.0	10.2
**^1^ *S*_5_**	2.4	0.5	10.2	12.1

## Data Availability

Data are available from the corresponding author.

## Supplementary Materials

The following are available online at https://www.mdpi.com/article/10.3390/molecules28227571/s1, Copies of the NMR spectra of compounds **9**, **10**, **11**, **12**, **13**, **18**, **19**, **20**, **21a**, **21b**, **1**, **2**, **3**, **4**, **5**, and **6**.

## Abbreviations

TEMPO—(2,2,6,6-Tetramethylpiperidin-1-yl)oxyl; DMF—Dimethylformamide; DCM—Dichloromethane; TBS—tert-butyldimethylsilyl; NBS—N-bromosuccinimide; TCAN—Trichloroacetonitrile; DCC—N,NCCdicyclohexylcarbodiimide; DMAP—4-Dimethylaminopyridine; TBSOTf—tert-Butyldimethylsilyl trifluoromethanesulfonate; MP—4-Methoxyphenyl; Pico—Picoloyl; TFA—Trifluoroacetic acid.
