# Supplementary material for: Stereocontrolled Synthesis and Conformational Analysis of a Series of Disaccharides α,β-d-GlcA-(1→3)-α-L-Fuc
Source: Molecules. 2023 Nov 13;28(22):7571. doi: 10.3390/molecules28227571 (PMC10673560; doi:10.3390/molecules28227571)

Supplementary Information

Stereocontrolled synthesis and conformational analysis of a series of disaccharides α,β-D-GlcA-(1→3)-α-L-Fuc

Alexey G. Gerbst, Dmitry Z. Vinnitsky, Alexandra I. Tokatly, Andrey S. Dmitrenok, Vadim B. Krylov, Nadezhda E. Ustuzhanina, Nikolay E. Nifantiev

Table of contents

Spectral data 3

(2-methyl-5-tert-butylphenyl) 4,6-O-(4-methoxybenzylidene)-1-thio-β-D-glucopyranoside (9). 4
Methyl [(2-methyl-5-tert-butylphenyl) 2,3-di-O-benzyl-1-thio-β-D-glucopyranosyl] uronate (10). 7
Methyl (2,3-di-O-benzyl-4-O-tert-butyldimethylsilyl-α,β-D-glucopyranosyl) uronate trichloroacetimidate (11). 10
Methyl (2,3-di-O-benzyl-4-O-picoloyl-α,β-D-glucopyranosyl) uronate trichloroacetimidate (12). 12
Methyl (2,3-di-O-benzyl-4-O-benzoyl-α,β-D-glucopyranosyl) uronate trichloroacetimidate (13). 15
Allyl 3,4-O-isopropylidene-2-O-(methyl 2,3-di-O-benzyl-4-O-tert-butyldimethylsilyl-α,β-D-glucopyranosyluronate)-α-L-fucopyranoside (18). 18
Allyl 3,4-O-isopropylidene-2-O-(methyl 2,3-di-O-benzyl-4-O-picoloyl-α,β-D-glucopyranosyluronate)-α-L-fucopyranoside (19). 22
Allyl 3,4-O-isopropylidene-2-O-(methyl 2,3-di-O-benzyl-4-O-benzoyl-α,β-D-glucopyranosyluronate)-α-L-fucopyranoside (20). 26
Propyl 2-O-(methyl 4-O-benzoyl-α-D-glucopyranosyluronate)-α-L-fucopyranoside (21a). 30
Propyl 2-O-(methyl 4-O-benzoyl-β-D-glucopyranosyluronate)-α-L-fucopyranoside (21b). 33
Propyl 2-O-(α-D-glucopyranosyluronic acid)-α-L-fucopyranoside sodium salt (1). 36
Propyl 2-O-(2,3-di-O-sulfo-α-D-glucopyranosyluronic acid)-3,4-di-O-sulfo-α-L-fucopyranoside sodium salt (2). 39
Propyl 2-O-(2,3,4-tri-O-sulfo-α-D-glucopyranosyluronic acid)-3,4-di-O-sulfo-α-L-fucopyranoside sodium salt (3). 42
Propyl 2-O-(β-D-glucopyranosyluronic acid)-α-L-fucopyranoside sodium salt (4). 45
Propyl 2-O-(2,3-di-O-sulfo-β-D-glucopyranosyluronic acid)-3,4-di-O-sulfo-α-L-fucopyranoside sodium salt (5). 49
Propyl 2-O-(2,3,4-tri-O-sulfo-β-D-glucopyranosyluronic acid)-3,4-di-O-sulfo-α-L-fucopyranoside sodium salt (6). 52

Spectral data

(2-methyl-5-tert-butylphenyl) 4,6-O-(4-methoxybenzylidene)-1-thio-β-D-glucopyranoside (9).

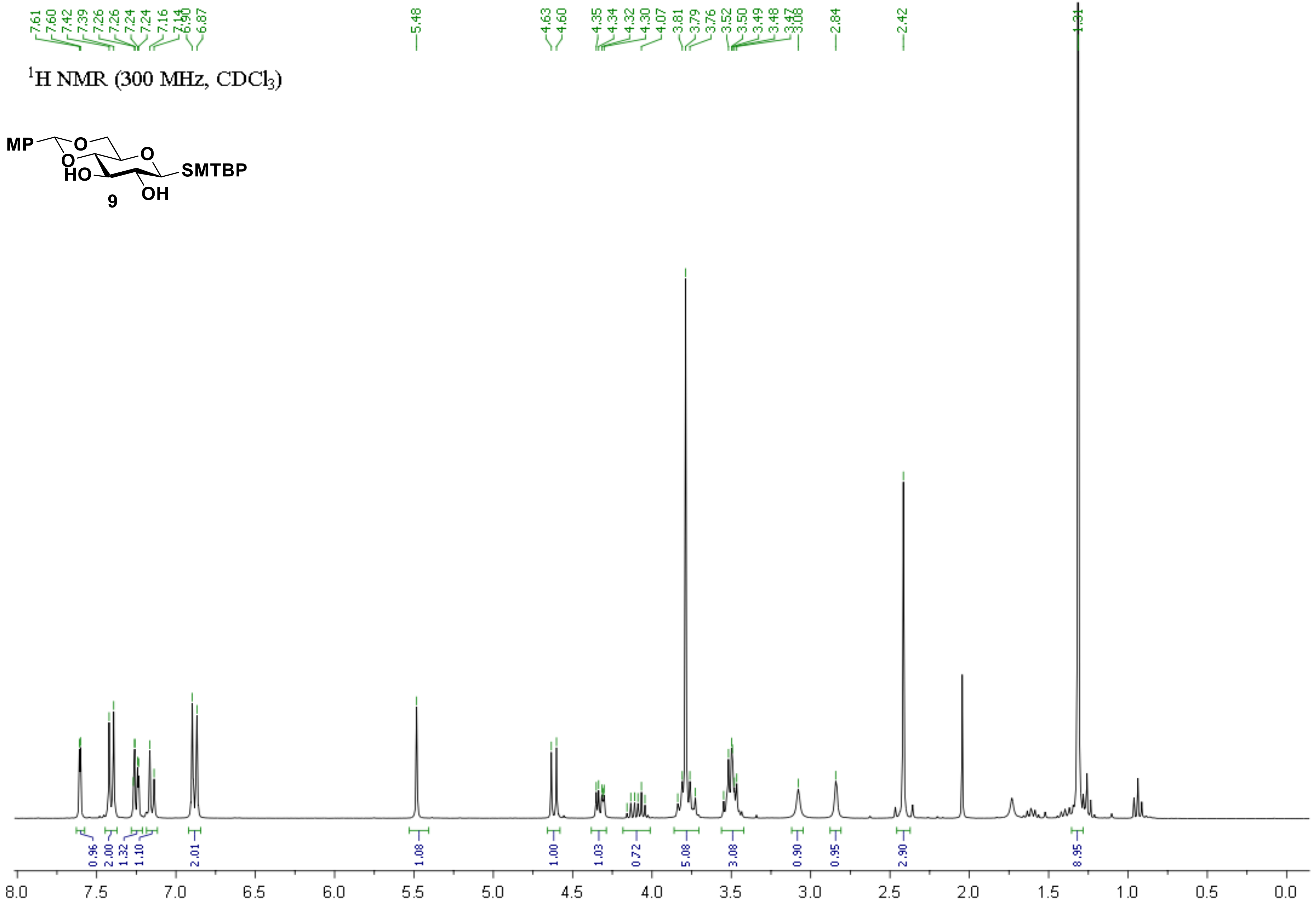

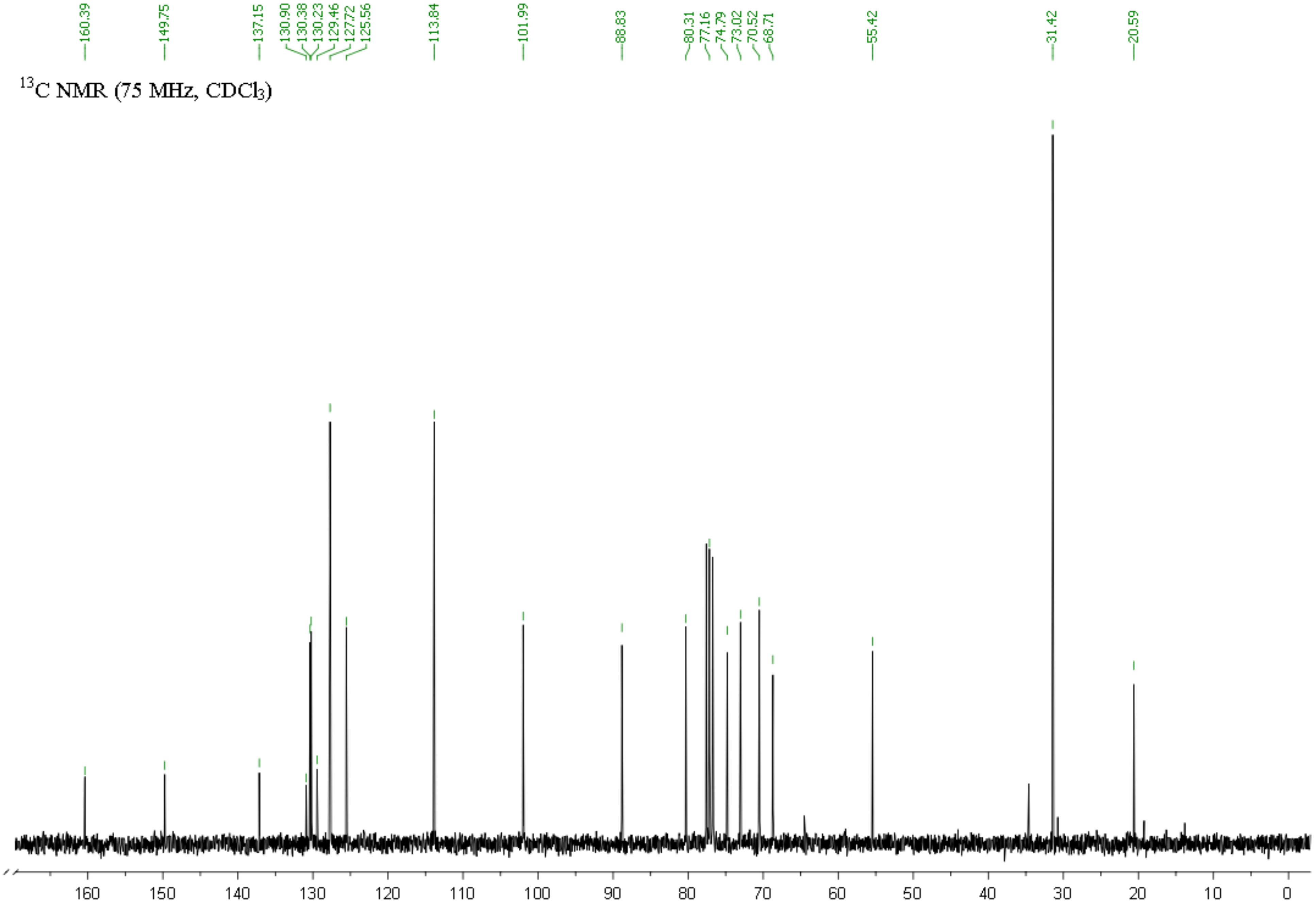

^{13}C NMR (75 MHz, $CDCl_3$)

Acquisition Parameter

Source Type	ESI	Ion Polarity	Positive	Set Nebulizer	0.5 Bar
Focus	Active			Set Dry Heater	180 ℃
Scan Begin	50 m/z	Set Capillary	4500 V	Set Dry Gas	4.0 l/min
Scan End	3000 m/z	Set End Plate Offset	-500 V	Set Divert Valve	Waste

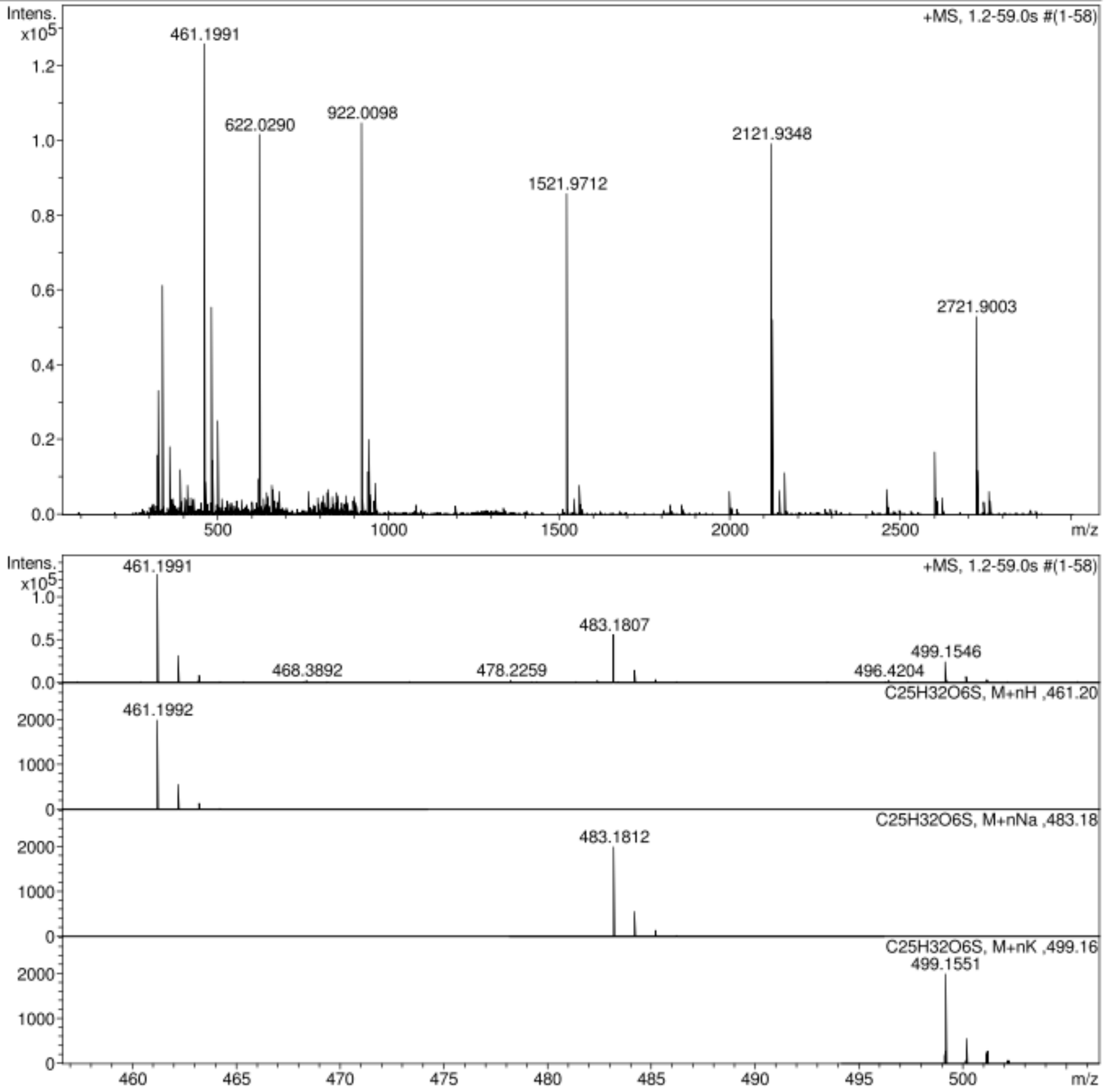

Methyl [(2-methyl-5-tert-butylphenyl) 2,3-di-O-benzyl-1-thio-β-D-glucopyranosyl] uronate (10).

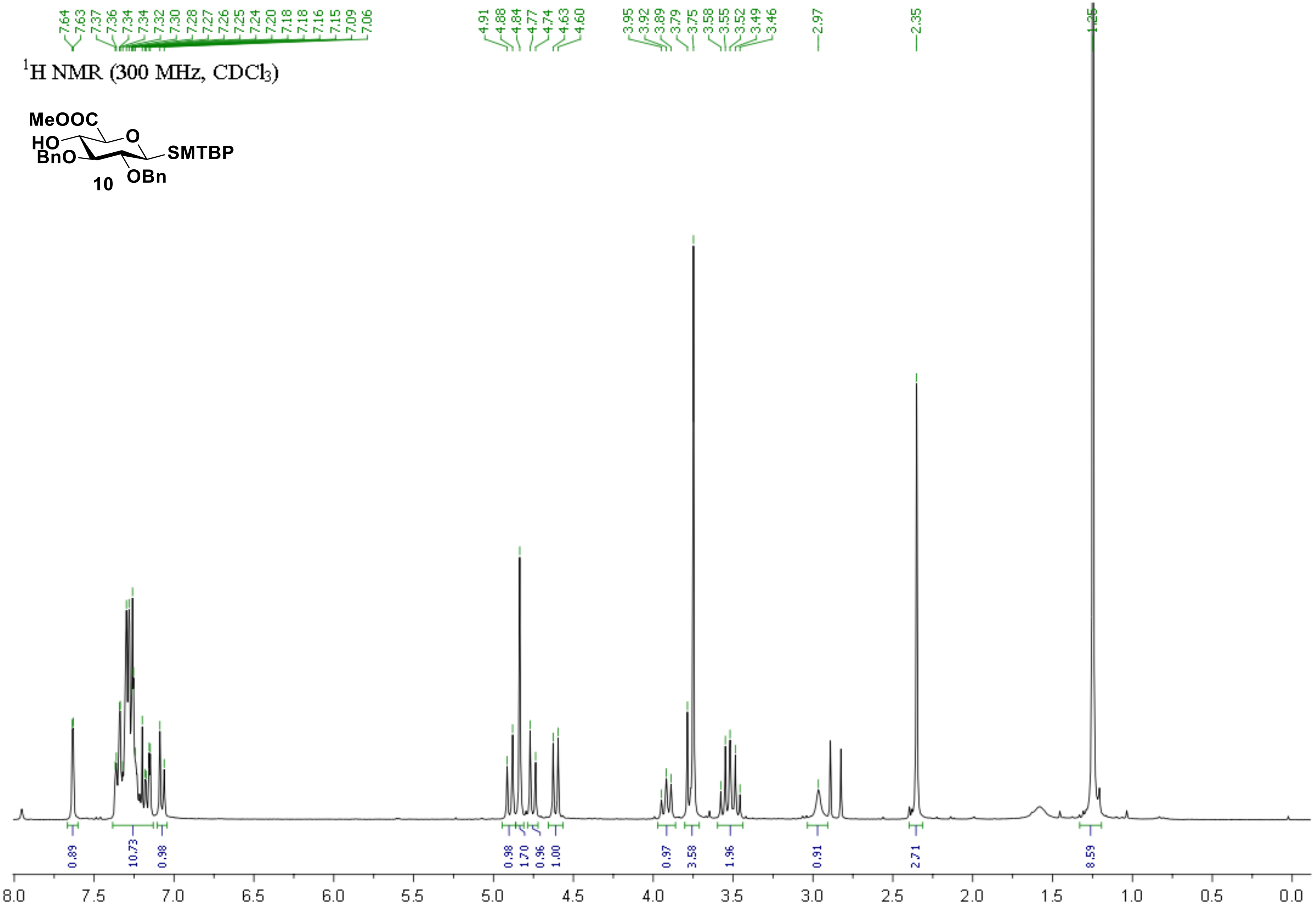

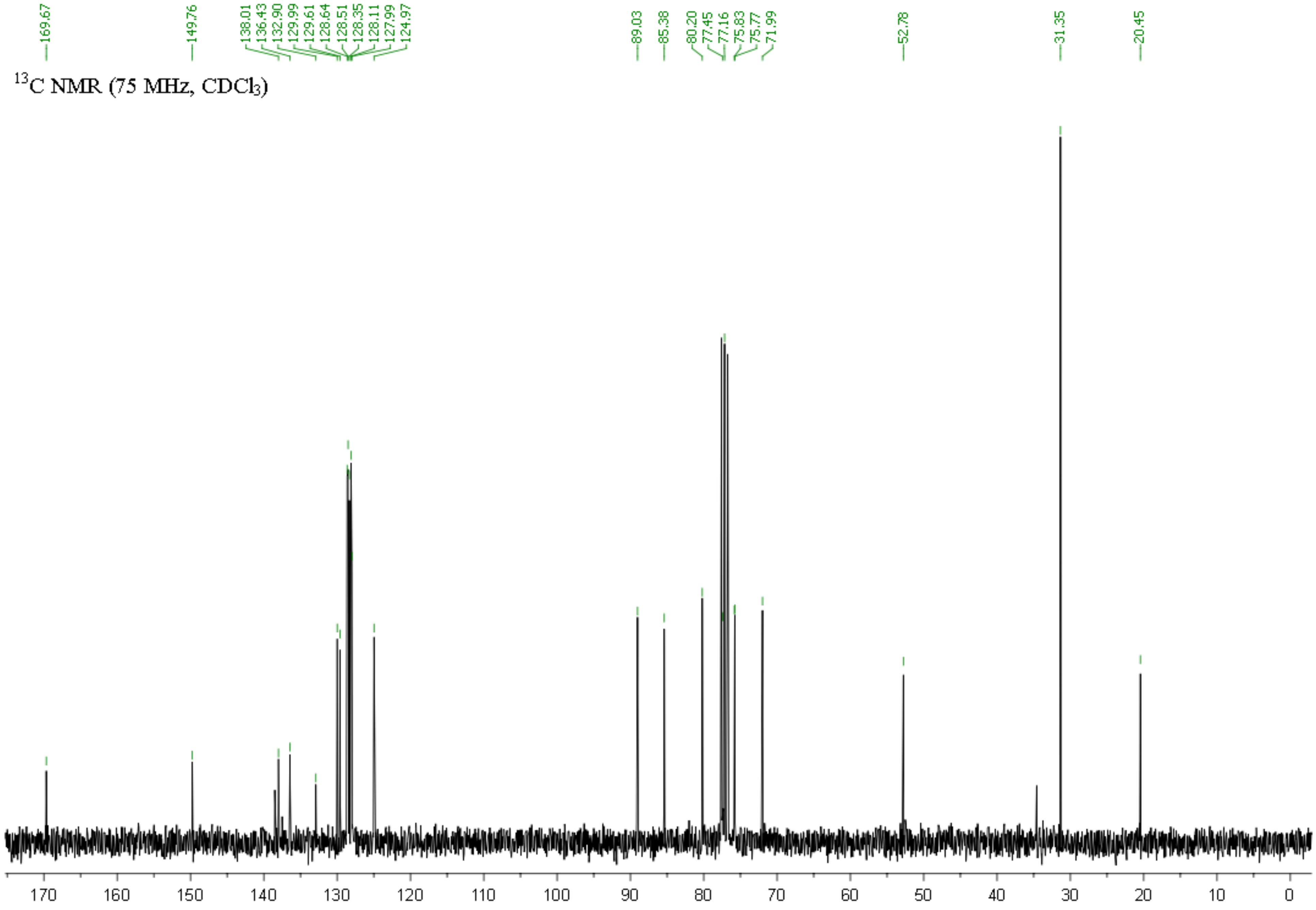
169.67
149.76
138.01
136.43
132.90
129.99
129.61
128.64
128.51
128.35
128.11
127.99
124.97
89.03
85.38
80.20
77.45
77.16
75.83
75.77
71.99
52.78
31.35
20.45
^{13}C NMR (75 MHz, CDCl$_3$)
170 160 150 140 130 120 110 100 90 80 70 60 50 40 30 20 10 0

Acquisition Parameter

Source Type	ESI	Ion Polarity	Positive	Set Nebulizer	0.5 Bar
Focus	Active			Set Dry Heater	180 °C
Scan Begin	50 m/z	Set Capillary	4500 V	Set Dry Gas	4.0 l/min
Scan End	3000 m/z	Set End Plate Offset	-500 V	Set Divert Valve	Waste

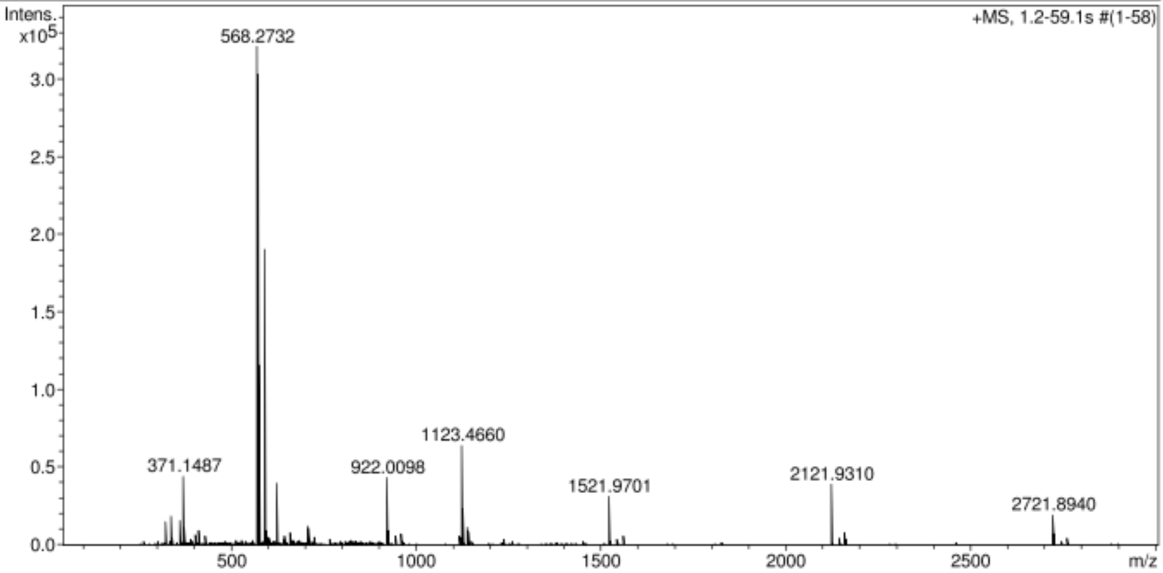

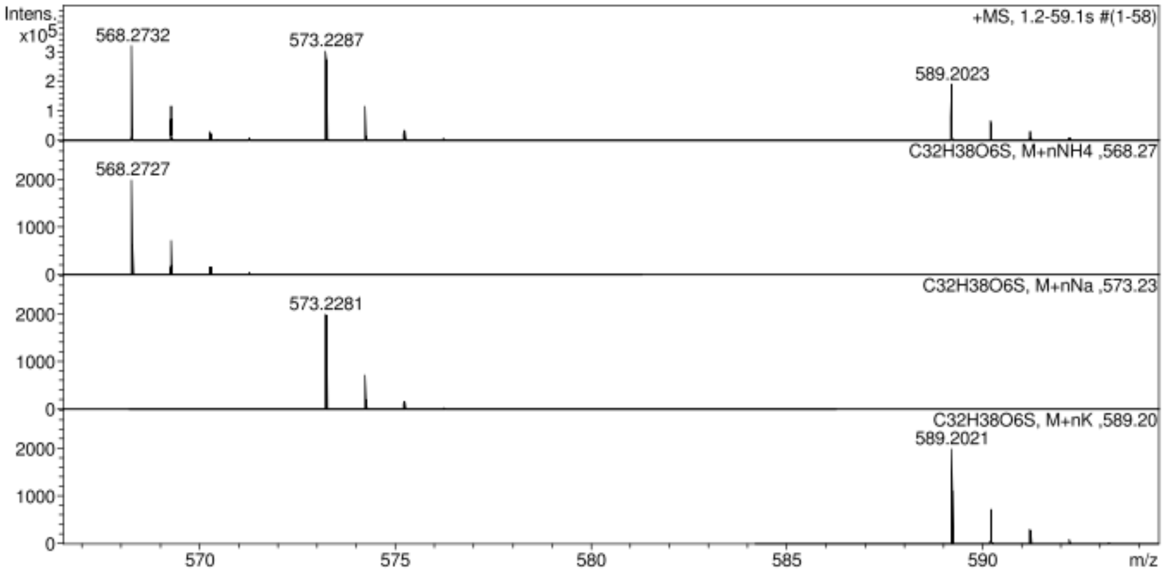

Methyl (2,3-di-O-benzyl-4-O-tert-butyldimethylsilyl-α,β-D-glucopyranosyl) uronate trichloroacetimidate (11).

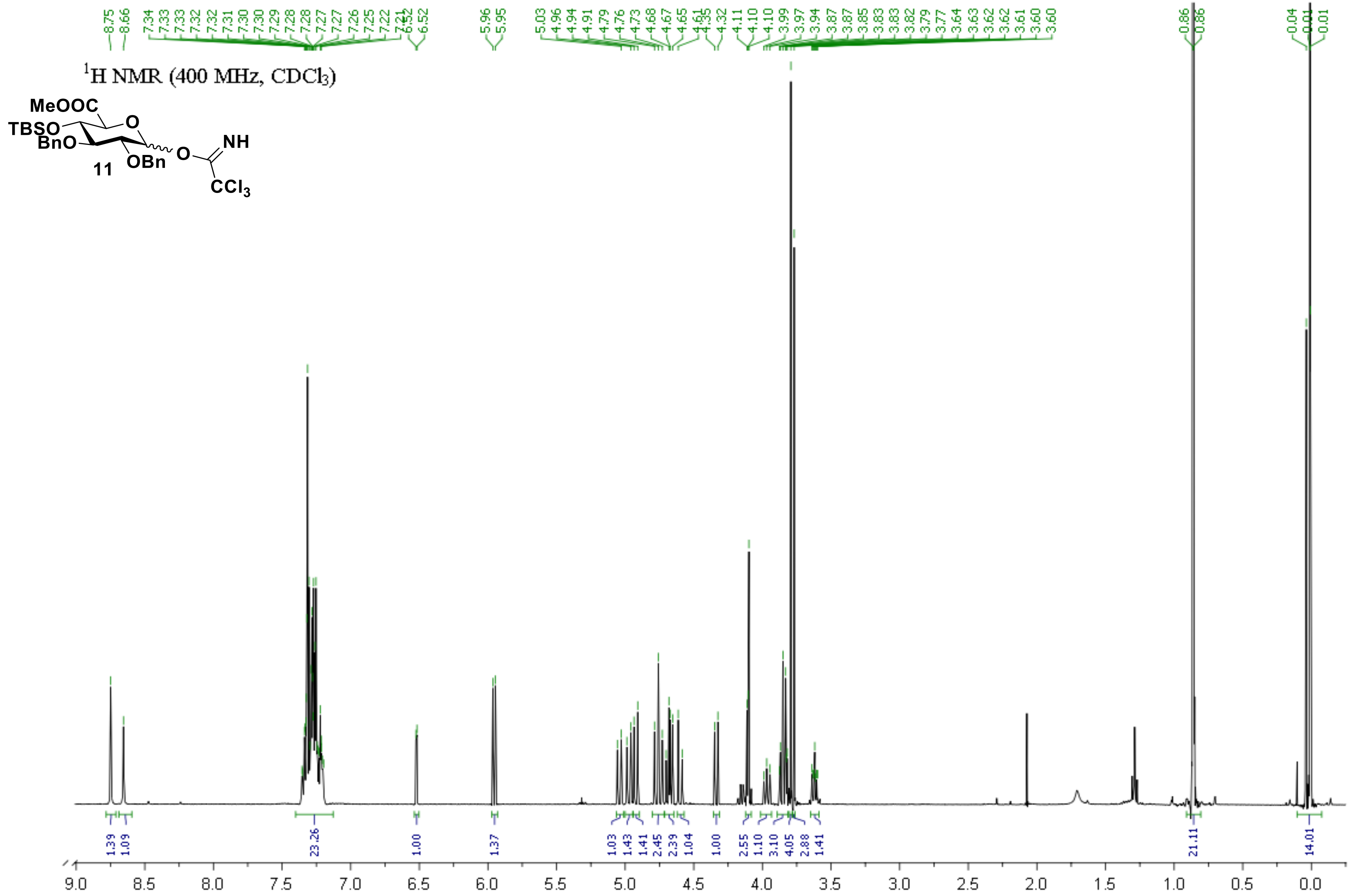

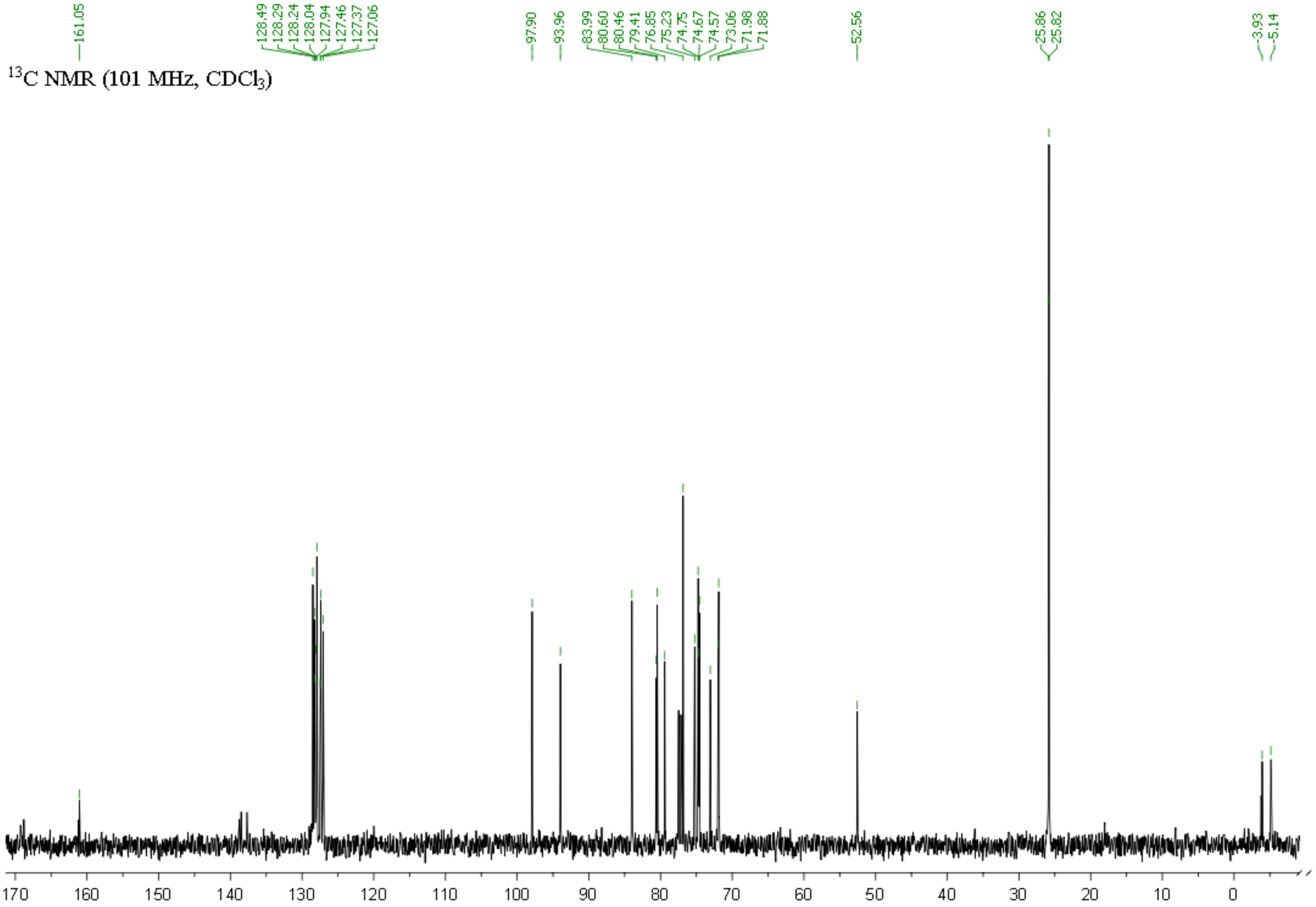
161.05
128.49
128.29
128.24
128.04
127.94
127.46
127.37
127.06
97.90
93.96
83.99
80.60
80.46
79.41
76.85
75.23
74.75
74.67
74.57
73.06
71.98
71.88
52.56
25.86
25.82
-3.93
-5.14
13C NMR (101 MHz, CDCl3)
170 160 150 140 130 120 110 100 90 80 70 60 50 40 30 20 10 0

Methyl (2,3-di-O-benzyl-4-O-picoloyl-α,β-D-glucopyranosyl) uronate trichloroacetimidate (12).

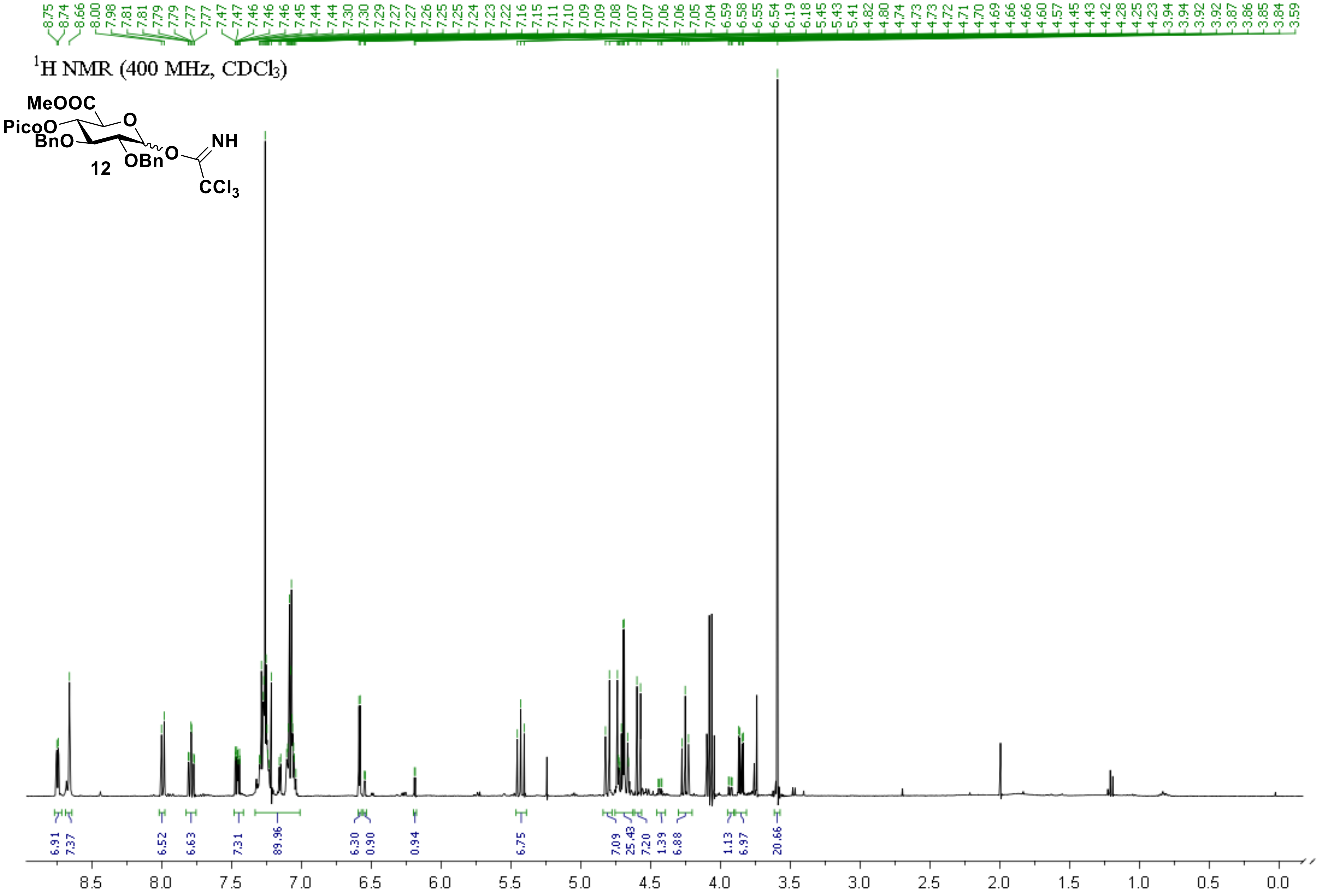

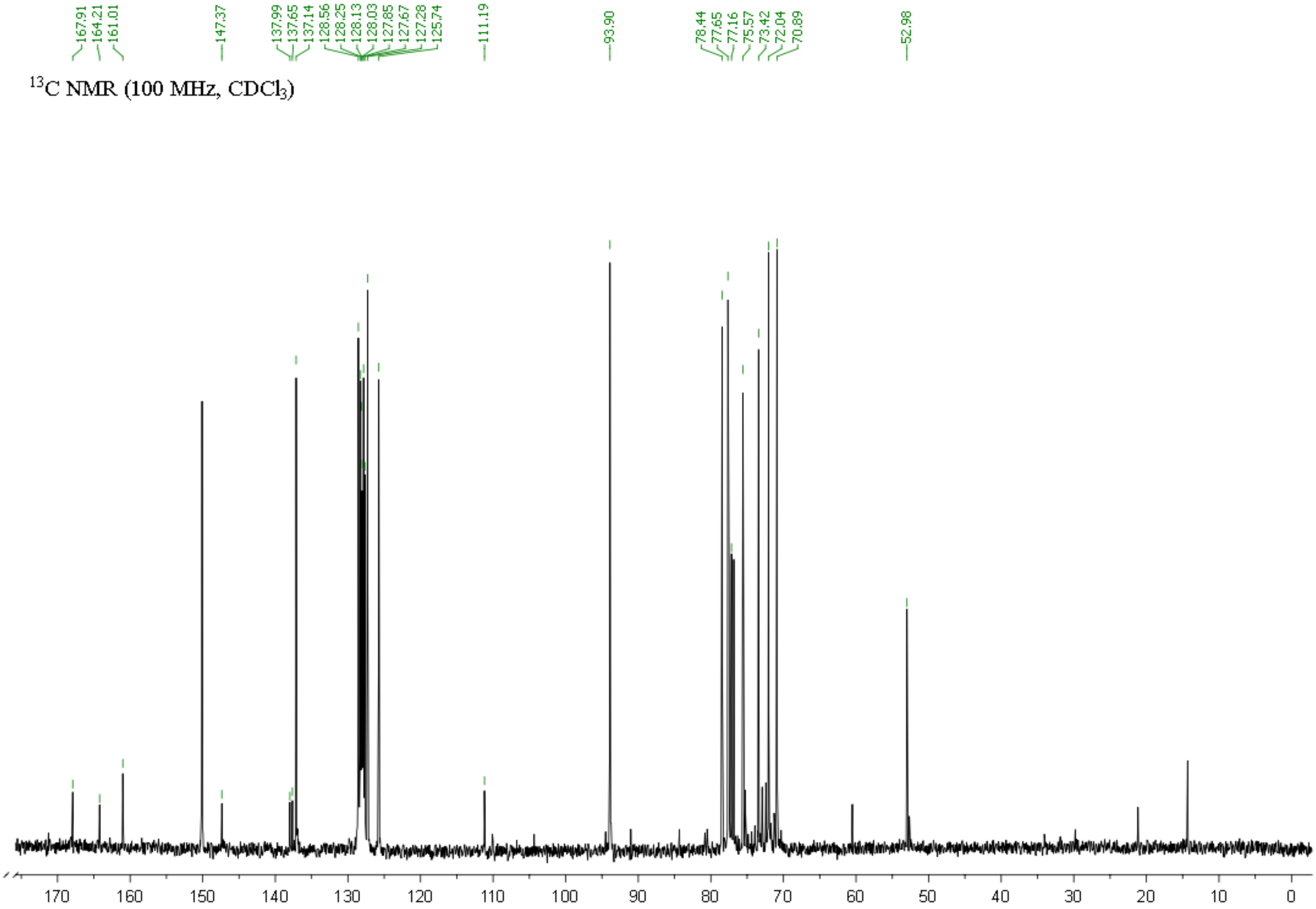

167.91
164.21
161.01
147.37
137.99
137.65
137.14
128.56
128.25
128.13
128.03
127.85
127.67
127.28
125.74
111.19
93.90
78.44
77.65
77.16
75.57
73.42
72.04
70.89
52.98
^{13}C NMR (100 MHz, CDCl$_3$)
170
160
150
140
130
120
110
100
90
80
70
60
50
40
30
20
10
0

Acquisition Parameter

Source Type	ESI	Ion Polarity	Positive	Set Nebulizer	0.5 Bar
Focus	Active			Set Dry Heater	180 °C
Scan Begin	50 m/z	Set Capillary	4500 V	Set Dry Gas	4.0 l/min
Scan End	3000 m/z	Set End Plate Offset	-500 V	Set Divert Valve	Waste

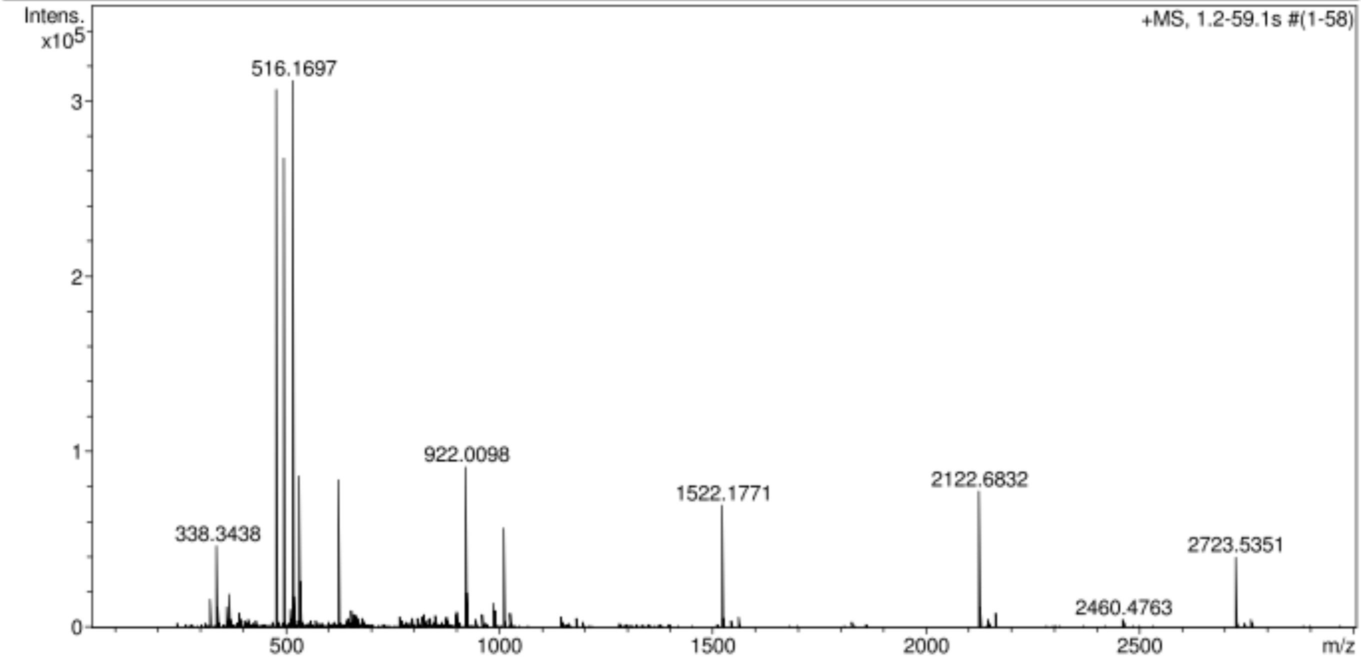

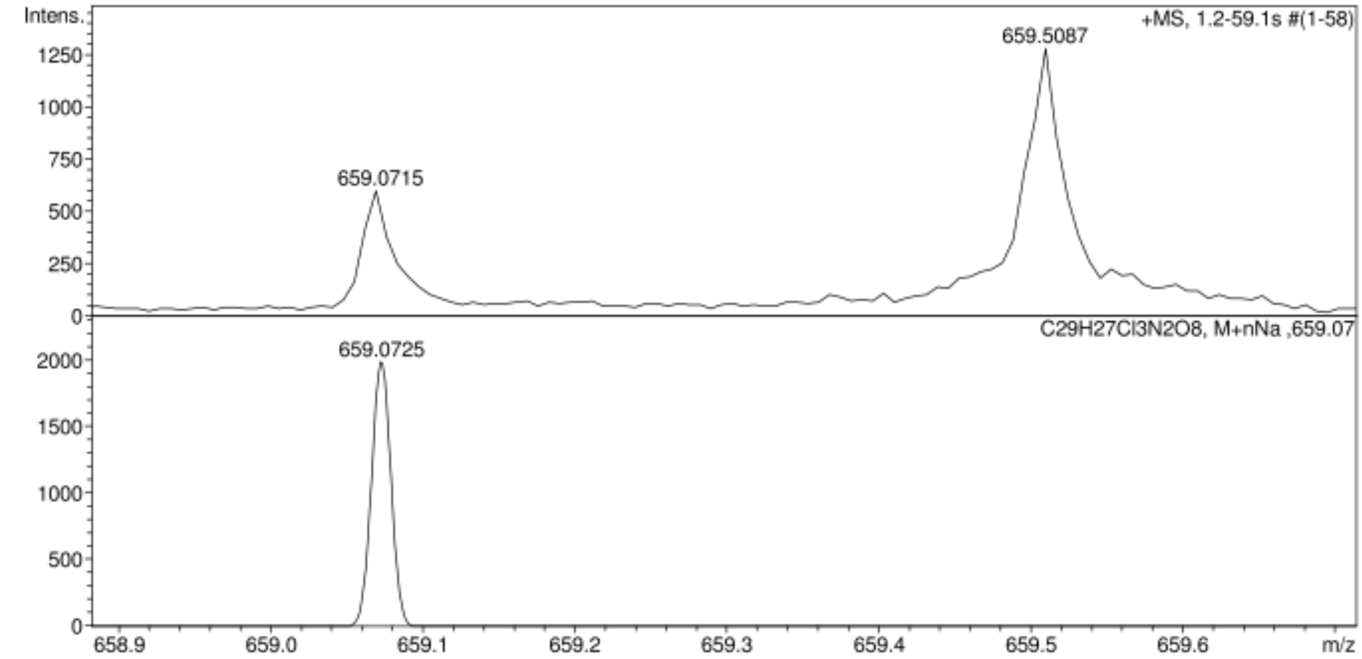

Methyl (2,3-di-O-benzyl-4-O-benzoyl-α,β-D-glucopyranosyl) uronate trichloroacetimidate (13).

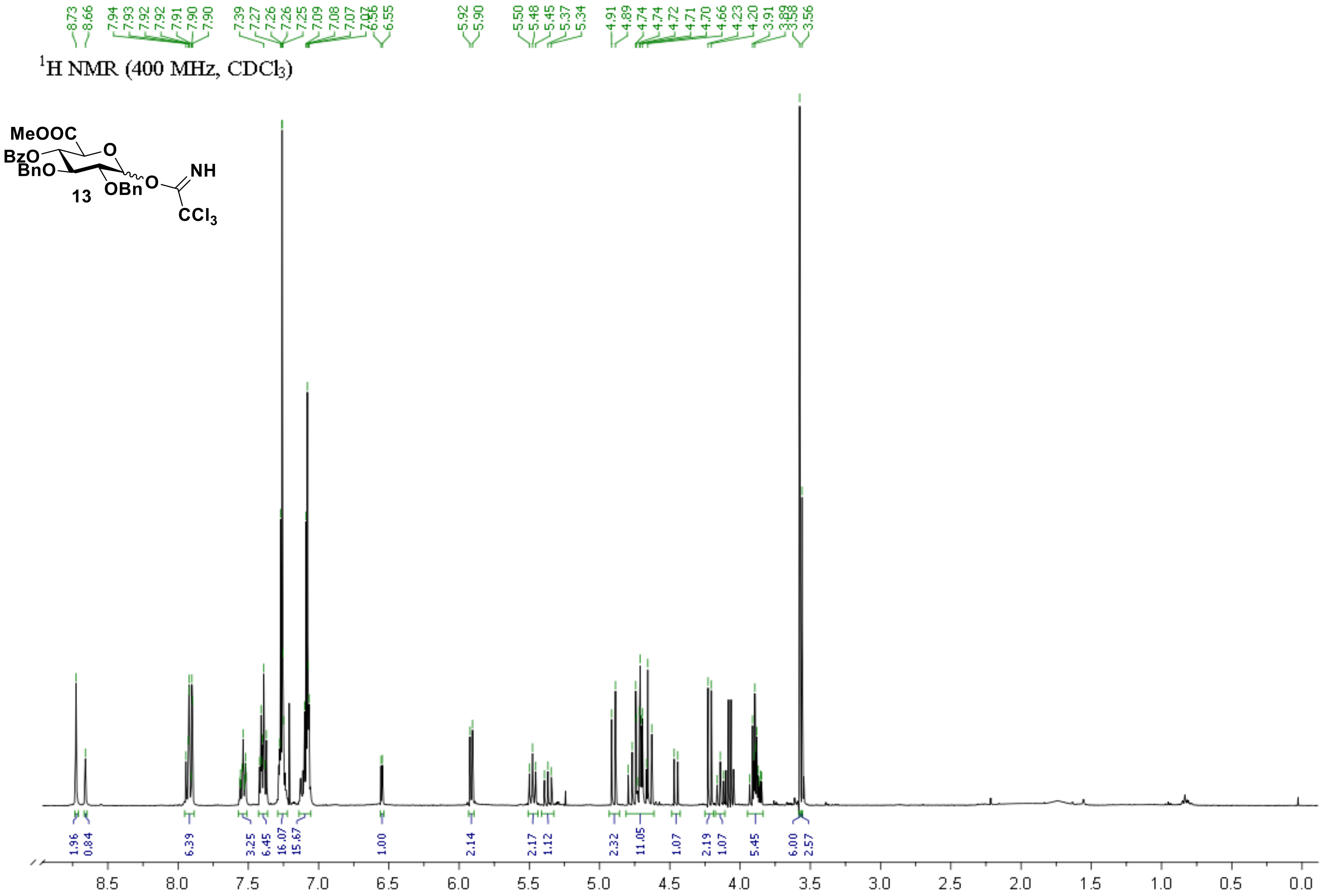

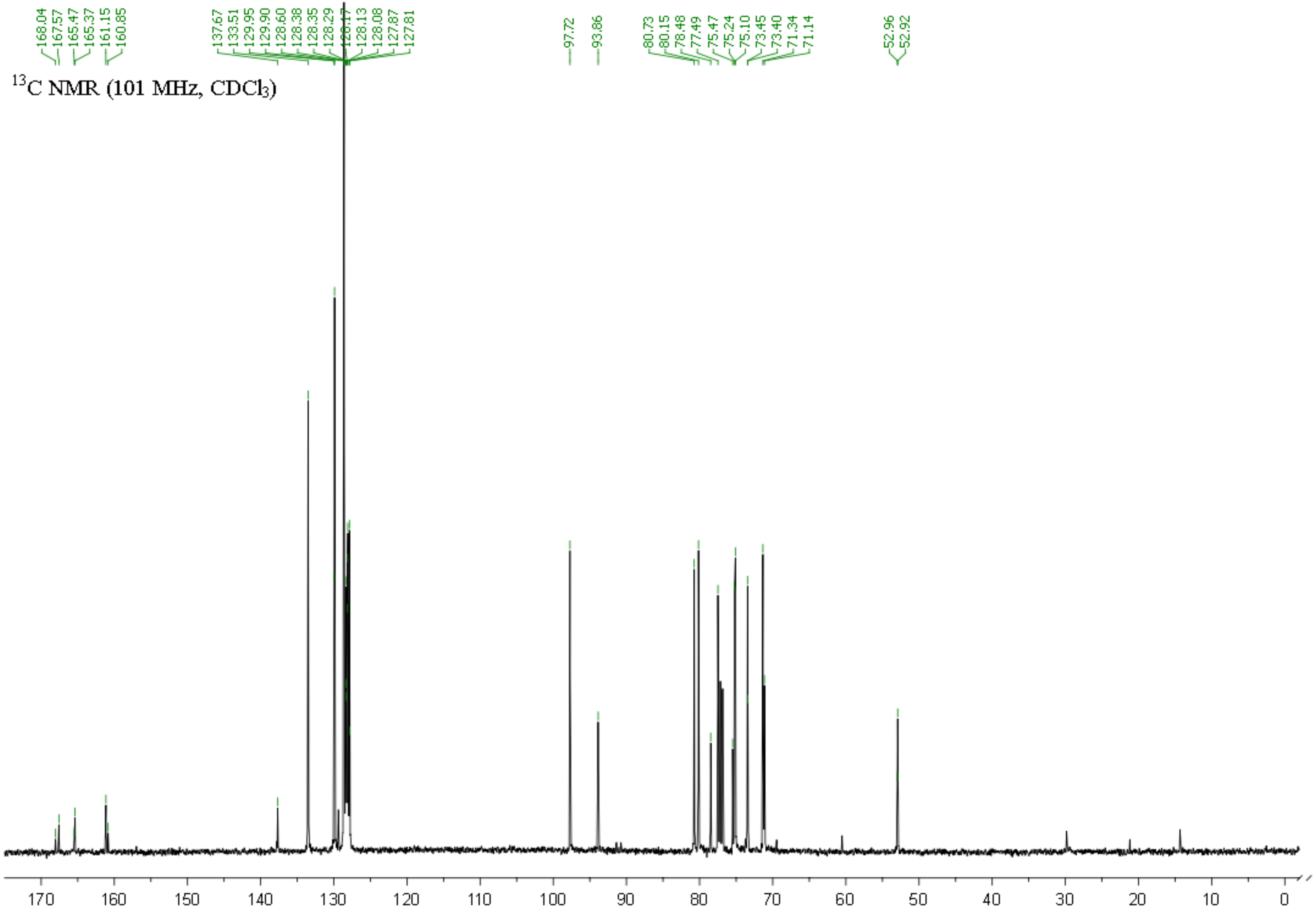
168.04
167.57
165.47
165.37
161.15
160.85
137.67
133.51
129.95
129.90
128.60
128.38
128.35
128.29
128.17
128.13
128.08
127.87
127.81
97.72
93.86
80.73
80.15
78.48
77.49
75.47
75.24
75.10
73.45
73.40
71.34
71.14
52.96
52.92
^{13}C NMR (101 MHz, CDCl$_3$)
170 160 150 140 130 120 110 100 90 80 70 60 50 40 30 20 10 0

Acquisition Parameter

Source Type	ESI	Ion Polarity	Positive	Set Nebulizer	0.5 Bar
Focus	Active			Set Dry Heater	180 °C
Scan Begin	50 m/z	Set Capillary	4500 V	Set Dry Gas	4.0 l/min
Scan End	3000 m/z	Set End Plate Offset	-500 V	Set Divert Valve	Waste

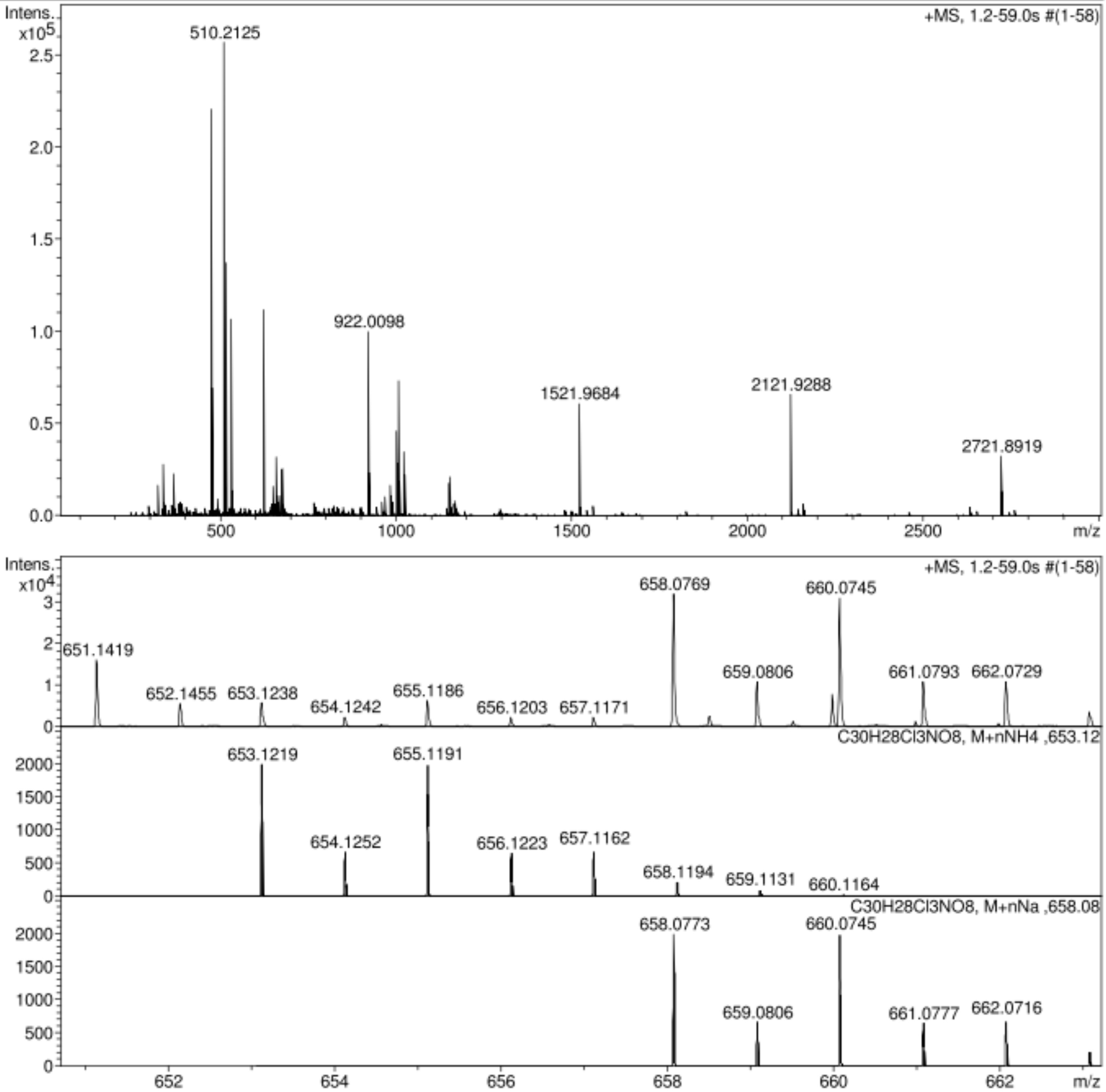

Allyl 3,4-O-isopropylidene-2-O-(methyl 2,3-di-O-benzyl-4-O-tert-butyldimethylsilyl-α,β-D-glucopyranosyluronate)-α-L-fucopyranoside (18).

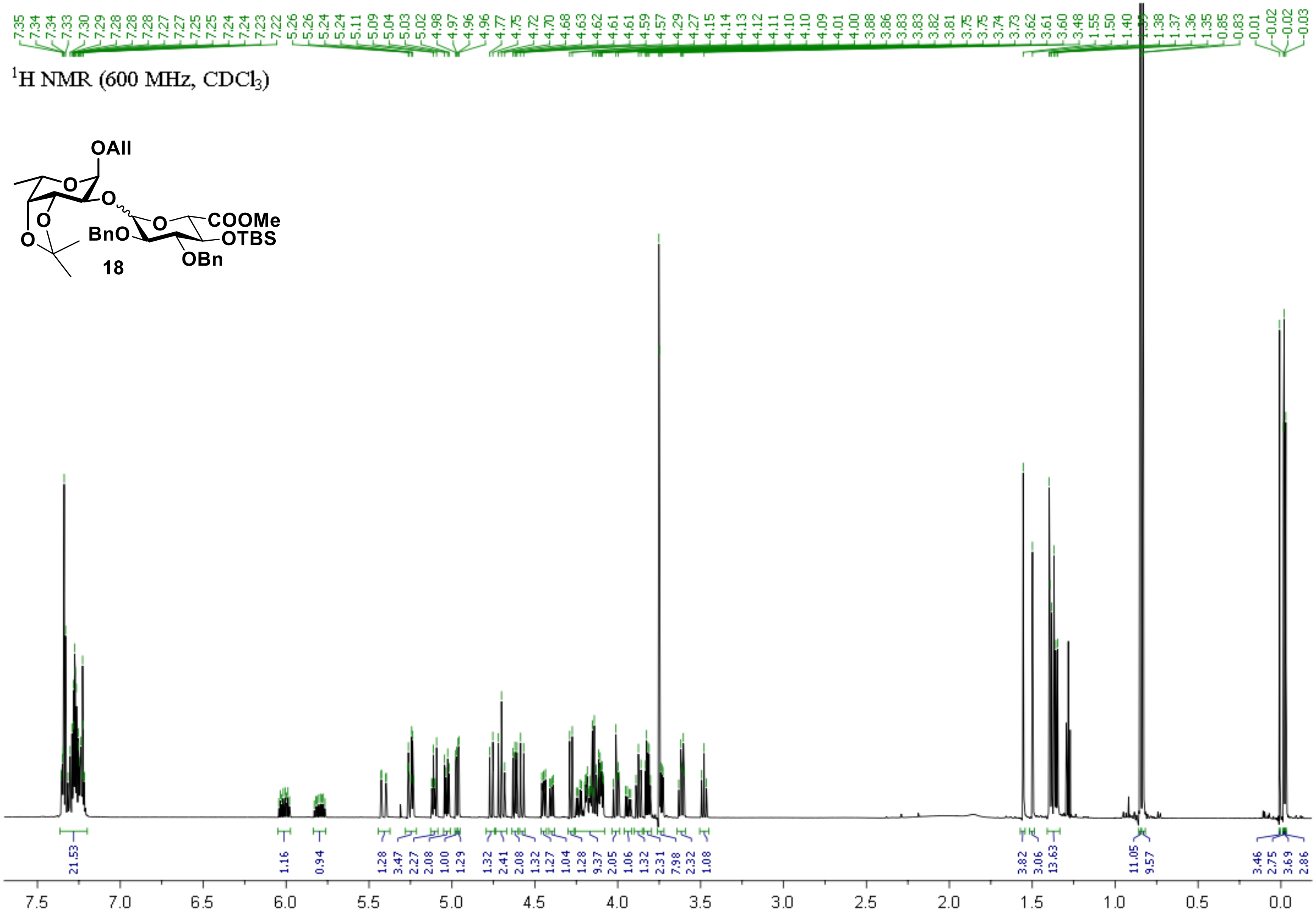

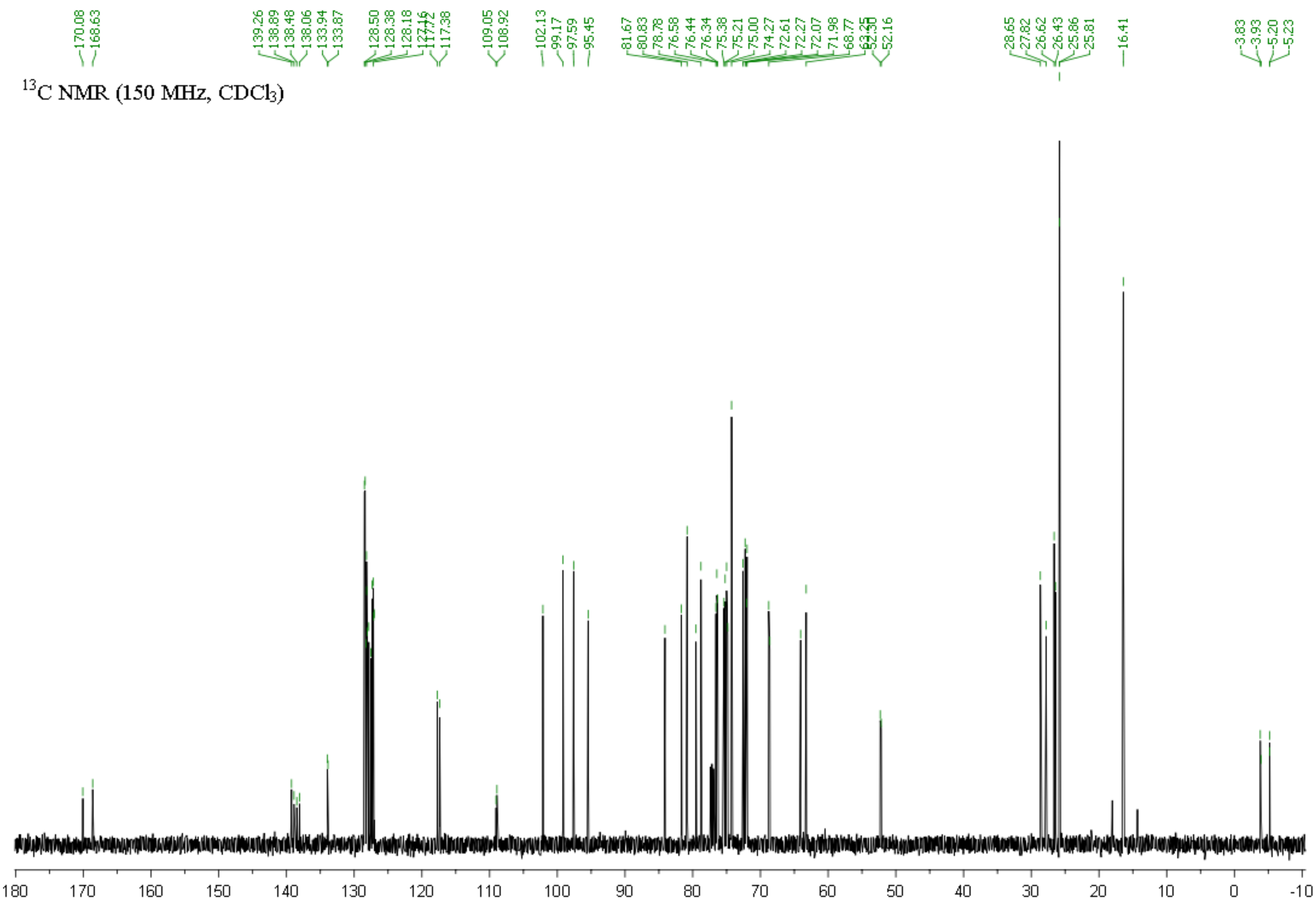
13C NMR (150 MHz, CDCl3)
170.08
168.63
139.26
138.89
138.48
138.06
133.94
133.87
128.50
128.38
128.18
109.05
108.92
102.13
99.17
97.59
95.45
81.67
80.83
78.78
76.58
76.44
76.34
75.38
75.21
75.00
74.27
72.61
72.27
72.07
71.98
68.77
52.16
28.65
27.82
26.62
26.43
25.86
25.81
16.41
-3.83
-3.93
-5.20
-5.23
180
170
160
150
140
130
120
110
100
90
80
70
60
50
40
30
20
10
0
-10

HSQC NMR ($CDCl_3$)

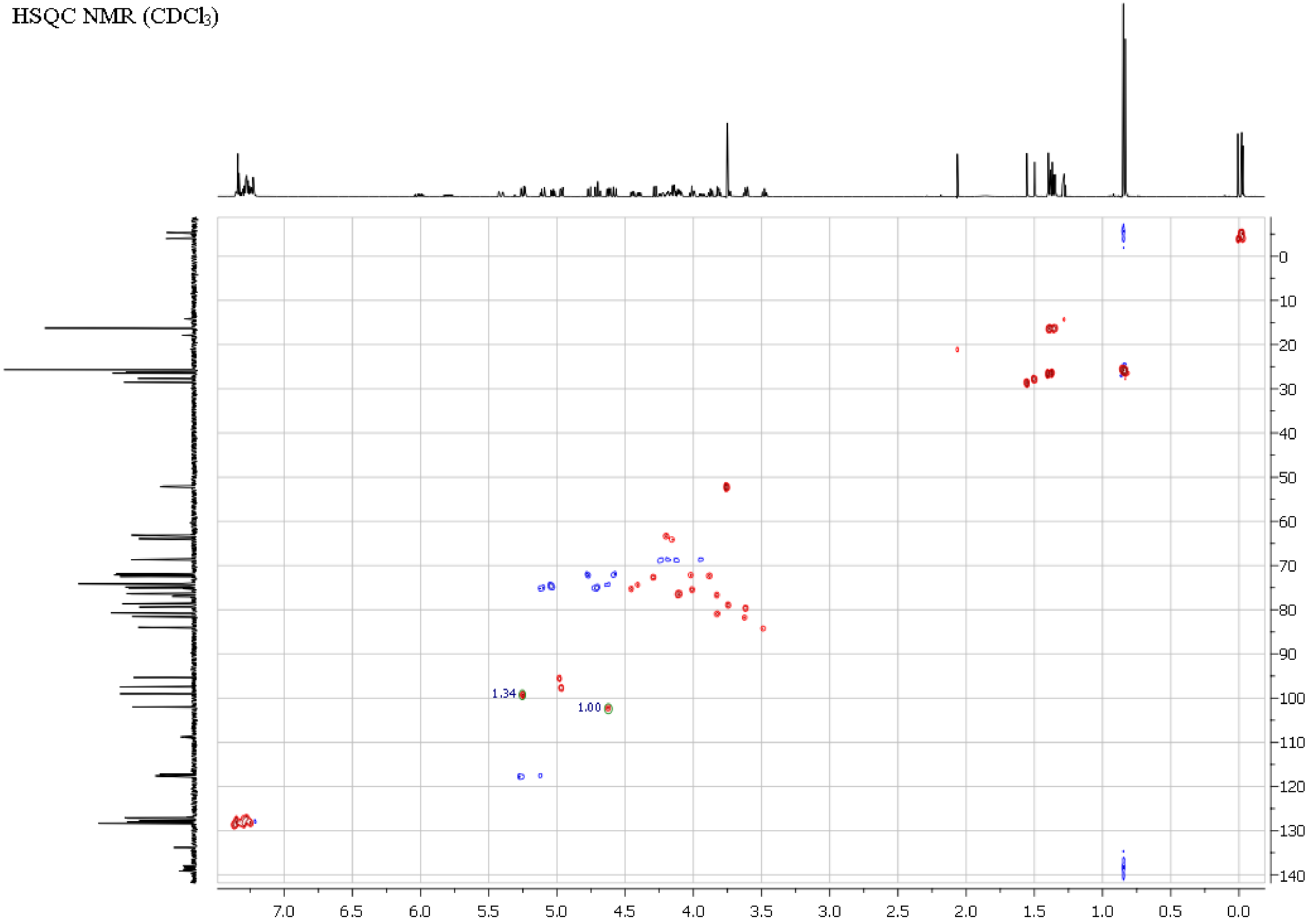

Acquisition Parameter

Source Type	ESI	Ion Polarity	Positive	Set Nebulizer	0.5 Bar
Focus	Active			Set Dry Heater	180 °C
Scan Begin	50 m/z	Set Capillary	4500 V	Set Dry Gas	4.0 l/min
Scan End	3000 m/z	Set End Plate Offset	-500 V	Set Divert Valve	Waste

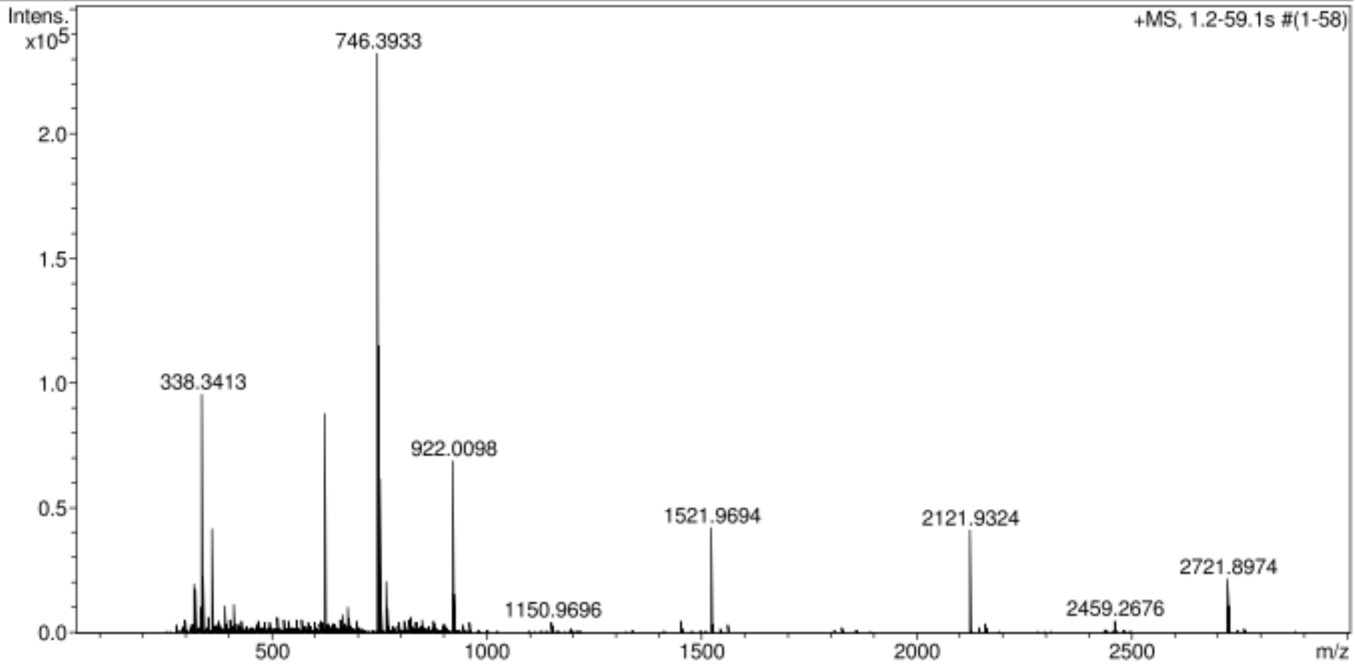

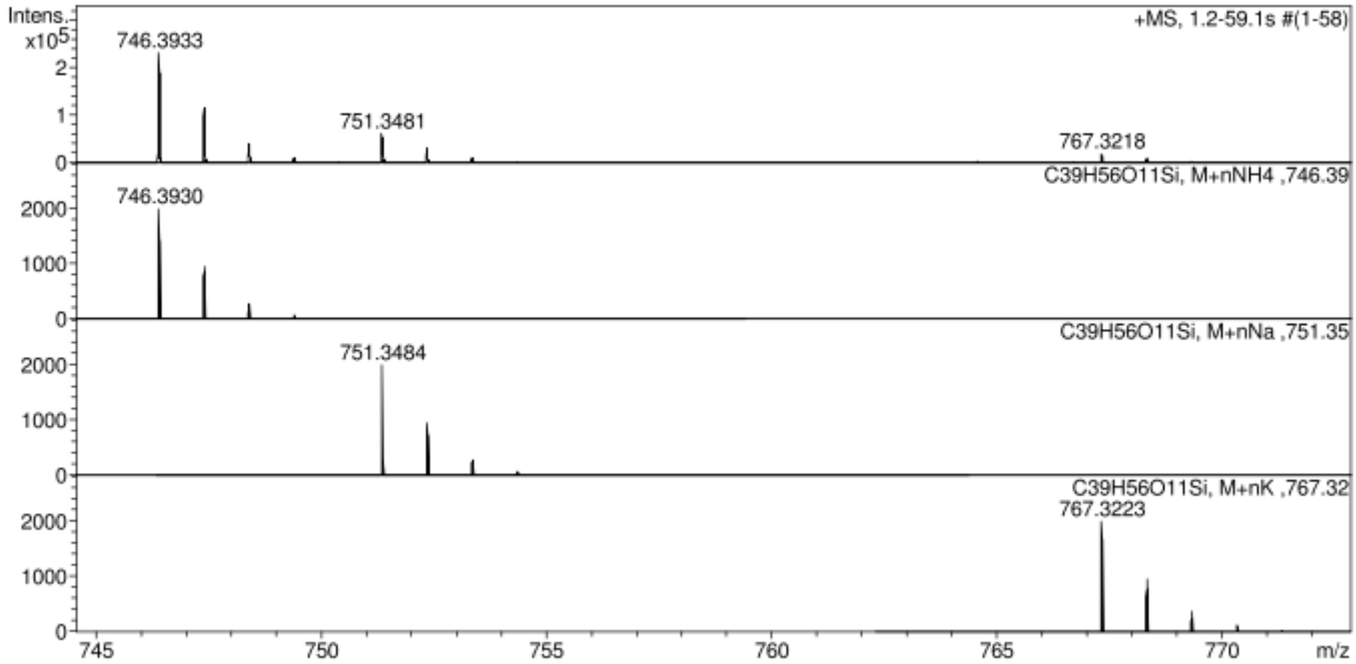

Allyl 3,4-O-isopropylidene-2-O-(methyl 2,3-di-O-benzyl-4-O-picoloyl-α,β-D-glucopyranosyluronate)-α-L-fucopyranoside (19).

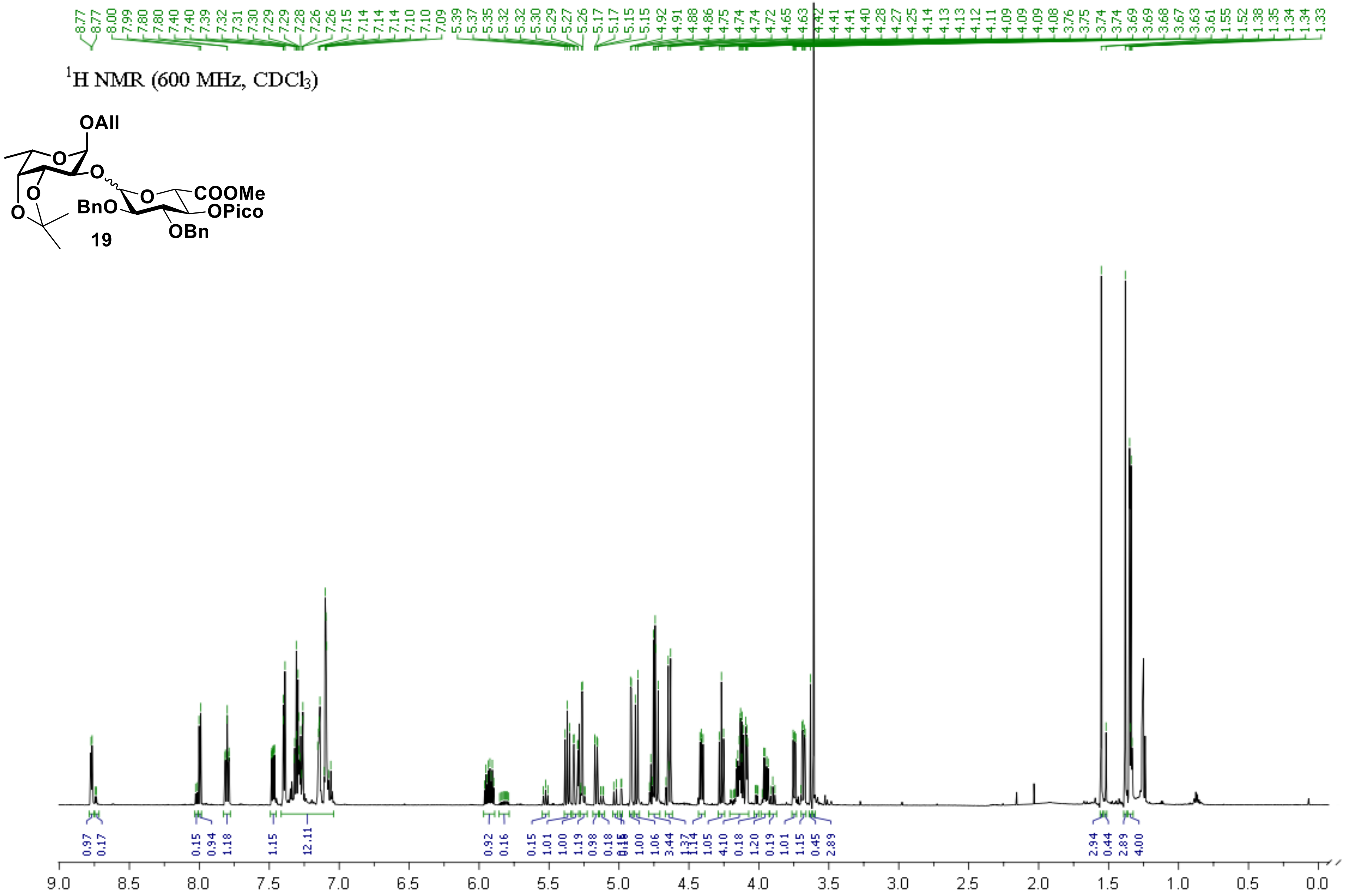

19

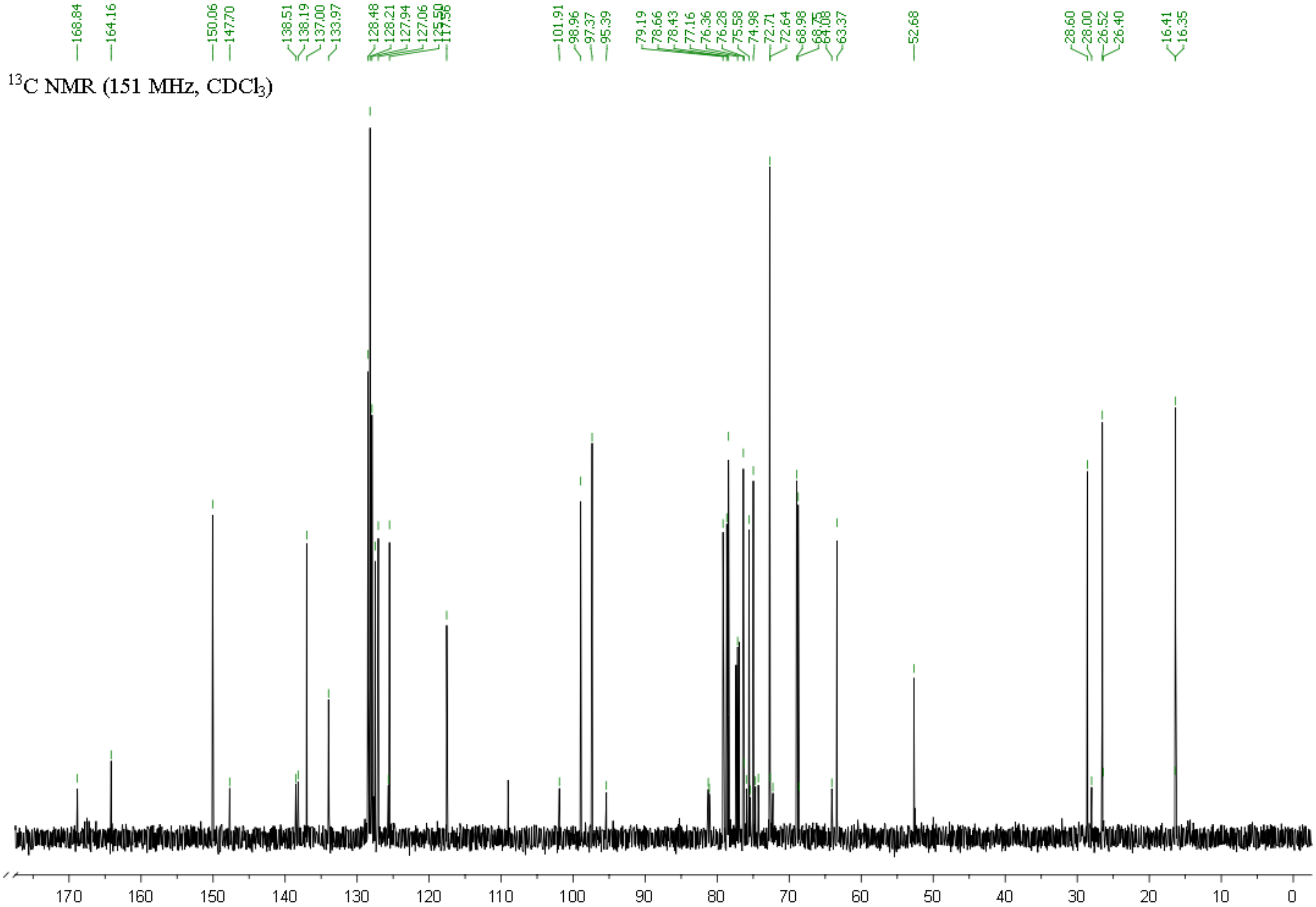
13C NMR (151 MHz, CDCl3)
168.84
164.16
150.06
147.70
138.51
138.19
137.00
133.97
128.48
128.21
127.94
127.06
125.50
117.56
101.91
98.96
97.37
95.39
79.19
78.66
78.43
77.16
76.36
76.28
75.58
74.98
72.71
72.64
68.98
68.75
64.08
63.37
52.68
28.60
28.00
26.52
26.40
16.41
16.35
170 160 150 140 130 120 110 100 90 80 70 60 50 40 30 20 10 0

HSQC NMR ($CDCl_3$)

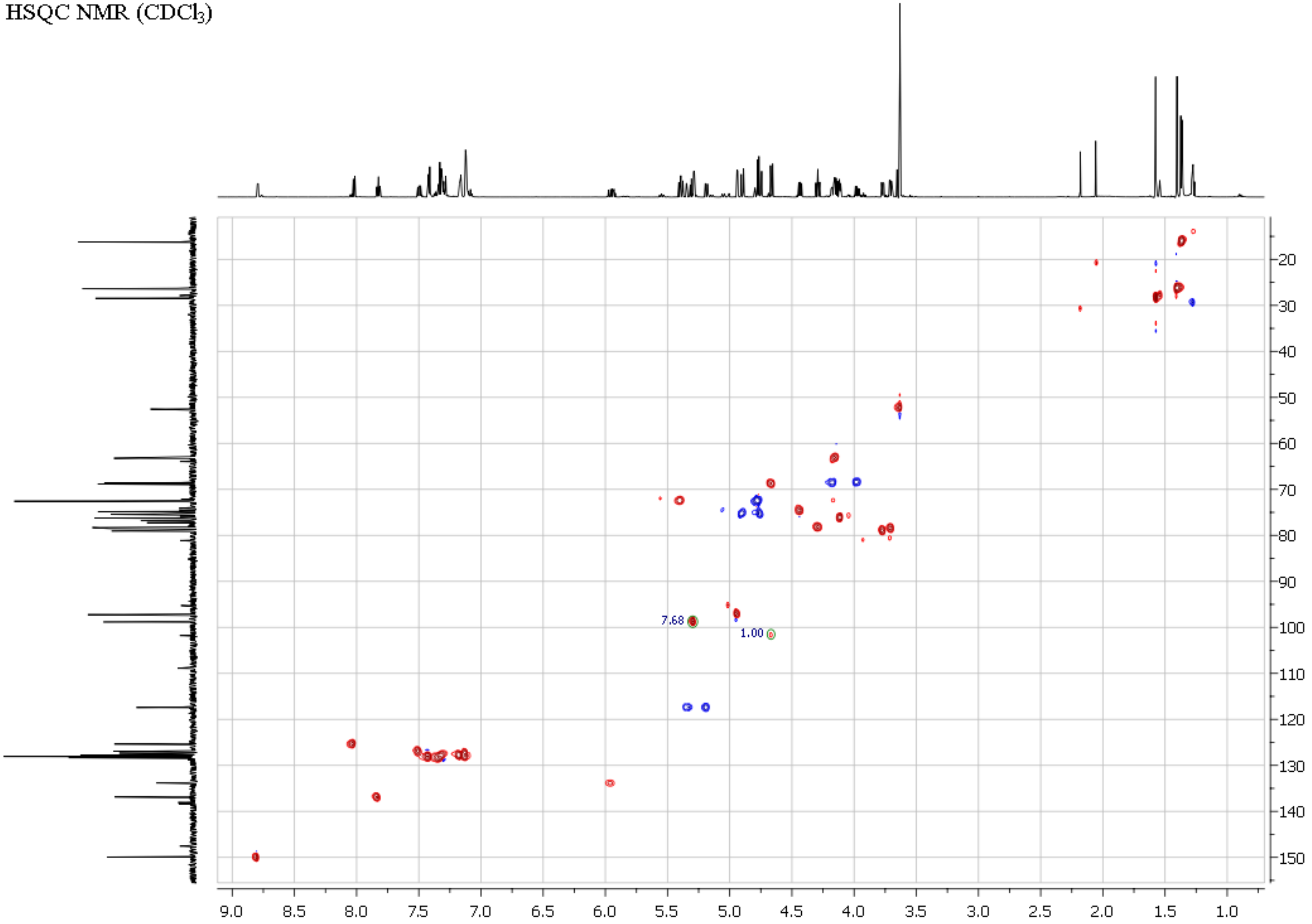

Acquisition Parameter

Source Type	ESI	Ion Polarity	Positive	Set Nebulizer	0.5 Bar
Focus	Active			Set Dry Heater	180 °C
Scan Begin	50 m/z	Set Capillary	4500 V	Set Dry Gas	4.0 l/min
Scan End	3000 m/z	Set End Plate Offset	-500 V	Set Divert Valve	Waste

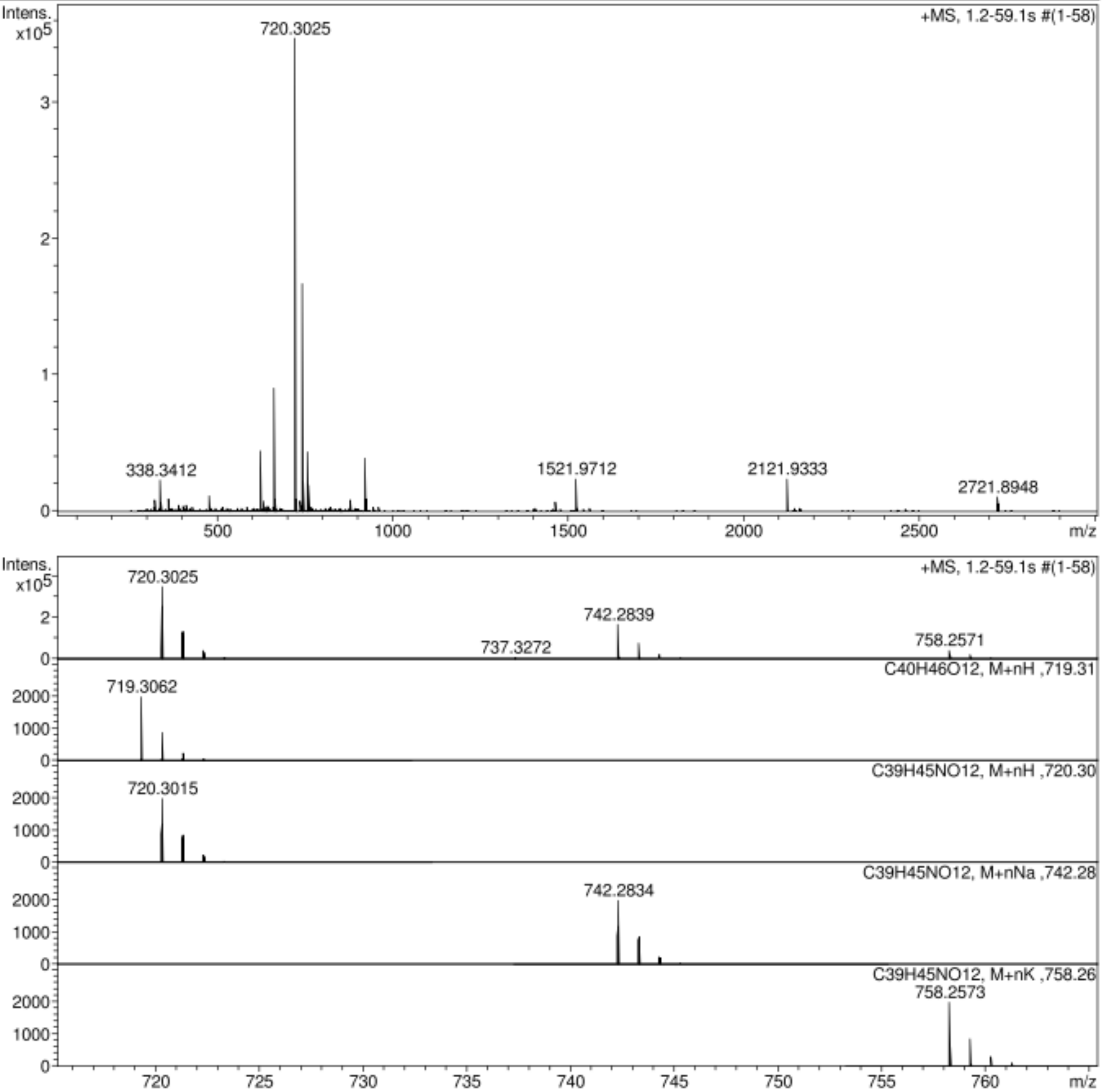

Allyl 3,4-O-isopropylidene-2-O-(methyl 2,3-di-O-benzyl-4-O-benzoyl-α,β-D-glucopyranosyluronate)-α-L-fucopyranoside (20).

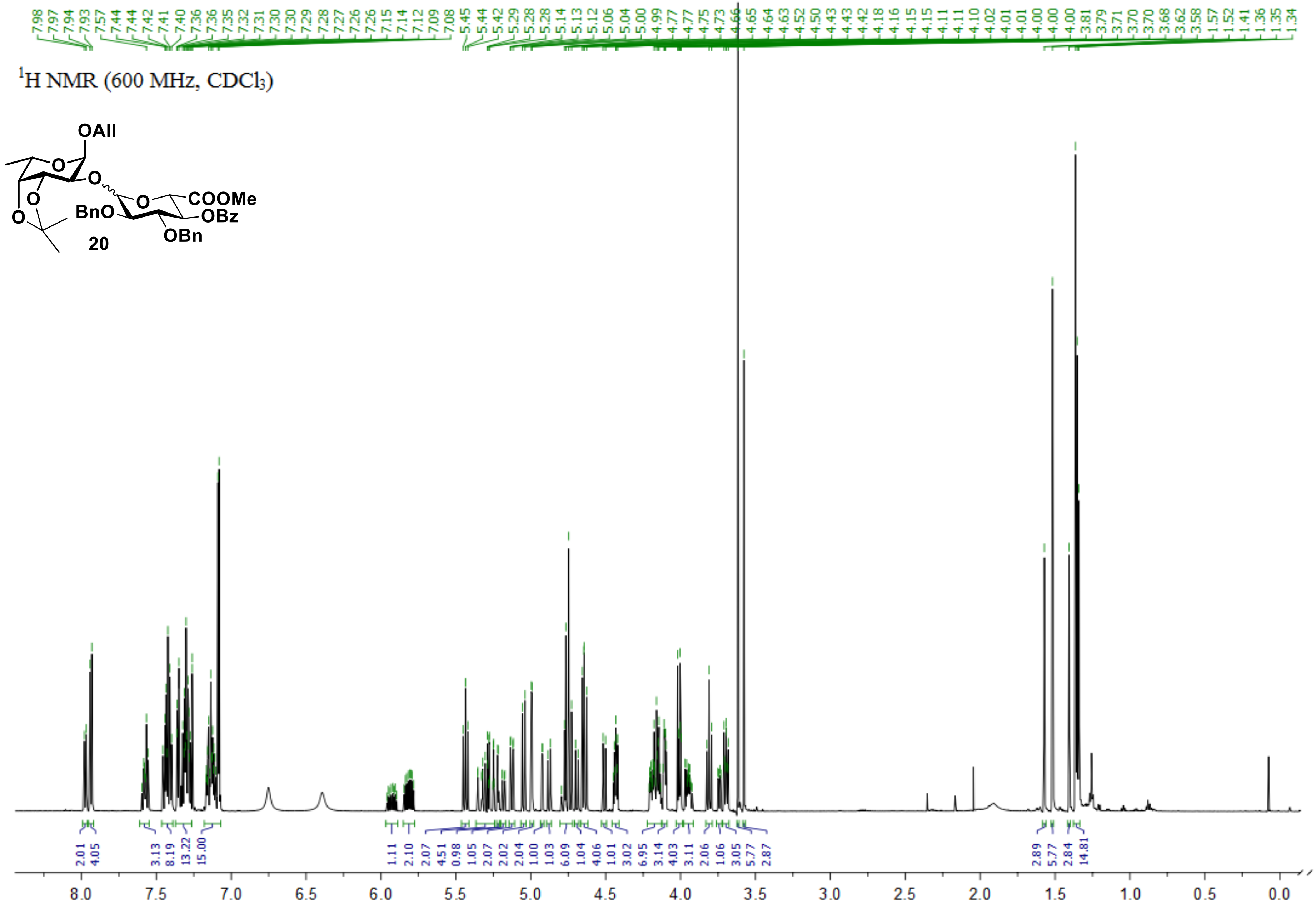

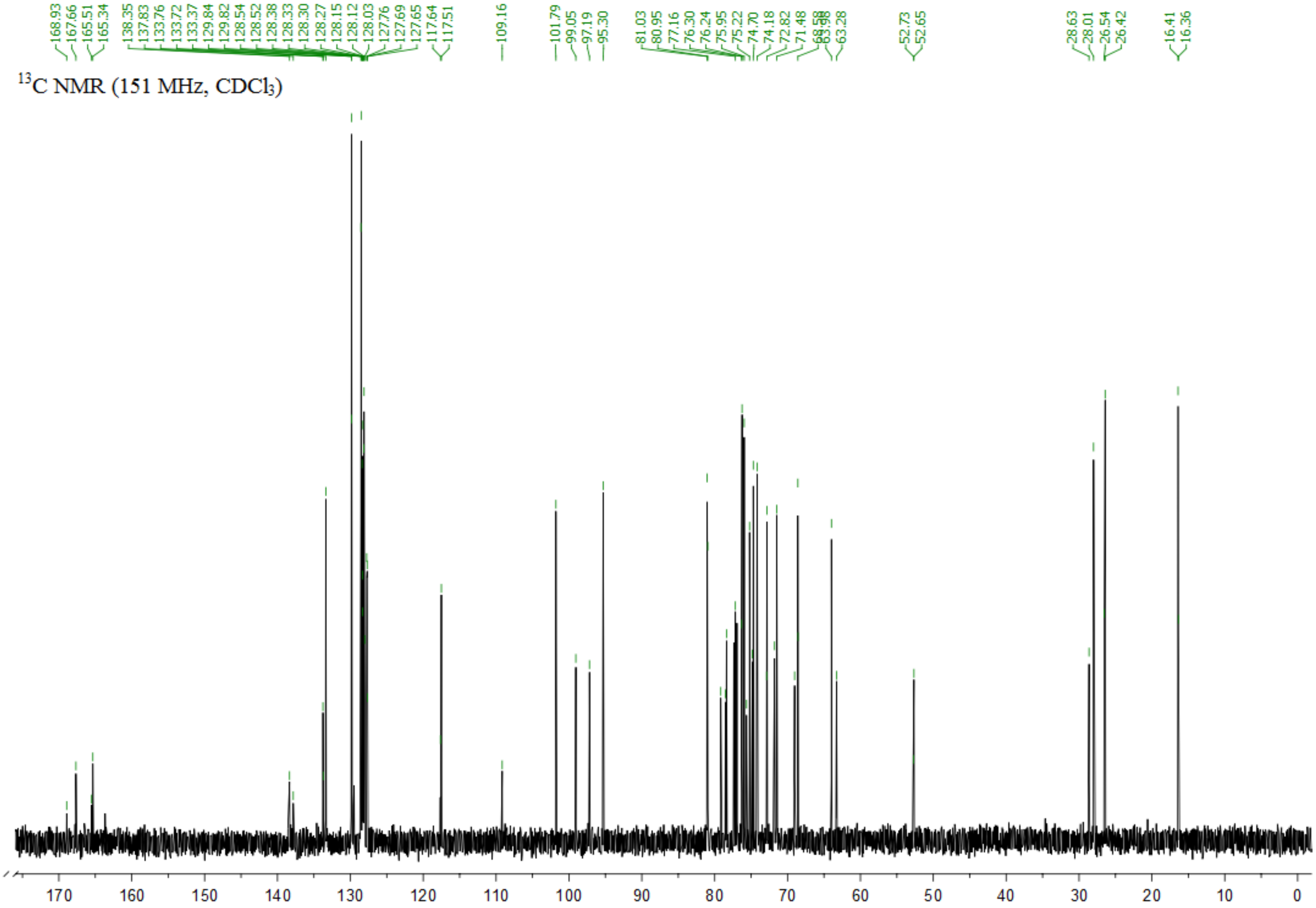

168.93
167.66
165.51
165.34
138.35
137.83
133.76
133.72
133.37
129.84
129.82
128.54
128.52
128.38
128.33
128.30
128.27
128.15
128.12
128.03
127.76
127.69
127.65
117.64
117.51
109.16
101.79
99.05
97.19
95.30
81.03
80.95
77.16
76.30
76.24
75.95
75.22
74.70
74.18
72.82
71.48
63.98
63.28
52.73
52.65
28.63
28.01
26.54
26.42
16.41
16.36
^{13}C NMR (151 MHz, $CDCl_3$)
170
160
150
140
130
120
110
100
90
80
70
60
50
40
30
20
10
0

HSQC NMR ($CDCl_3$)

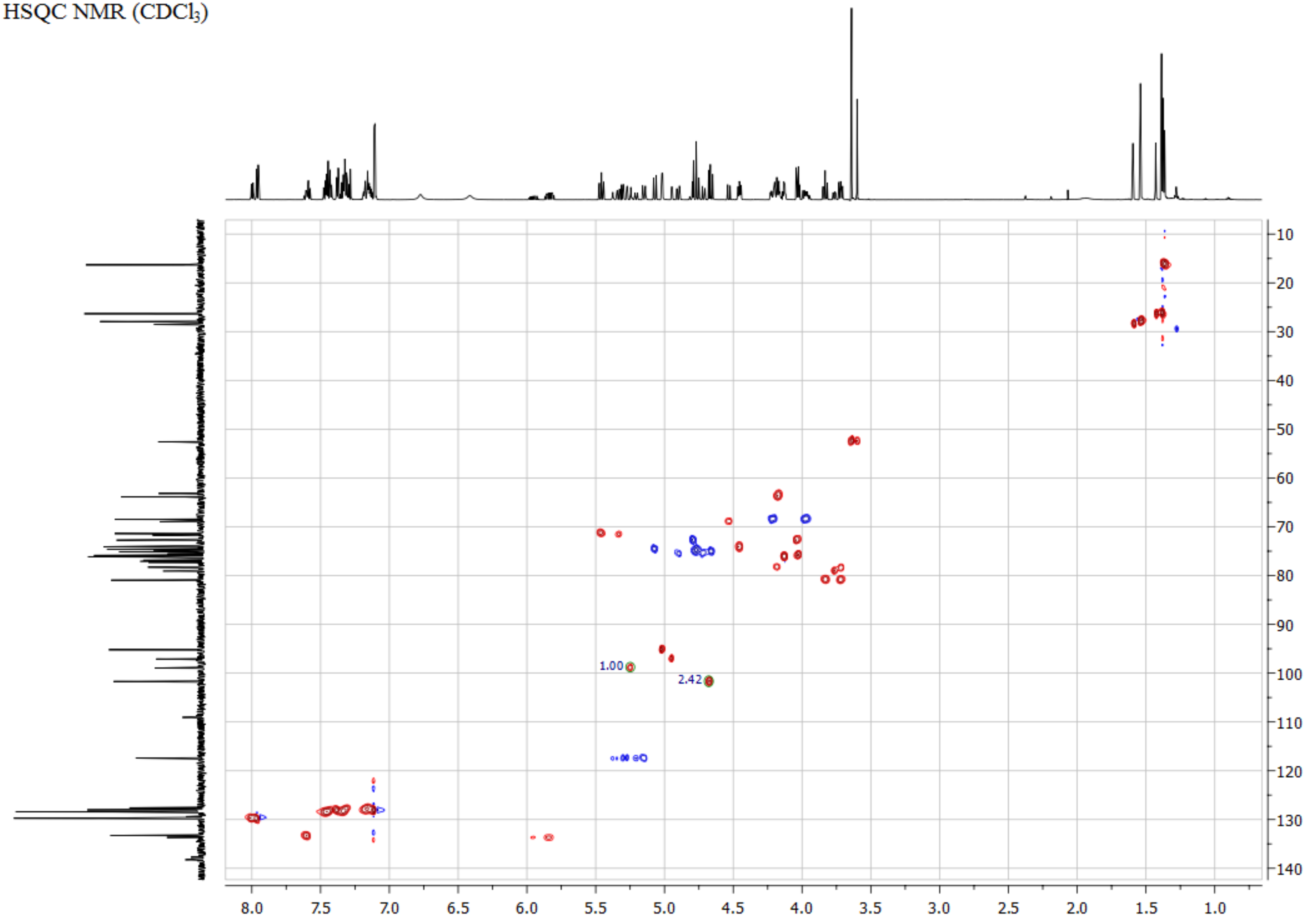

Acquisition Parameter

Source Type	ESI	Ion Polarity	Positive	Set Nebulizer	0.5 Bar
Focus	Active			Set Dry Heater	180 °C
Scan Begin	50 m/z	Set Capillary	4500 V	Set Dry Gas	4.0 l/min
Scan End	3000 m/z	Set End Plate Offset	-500 V	Set Divert Valve	Waste

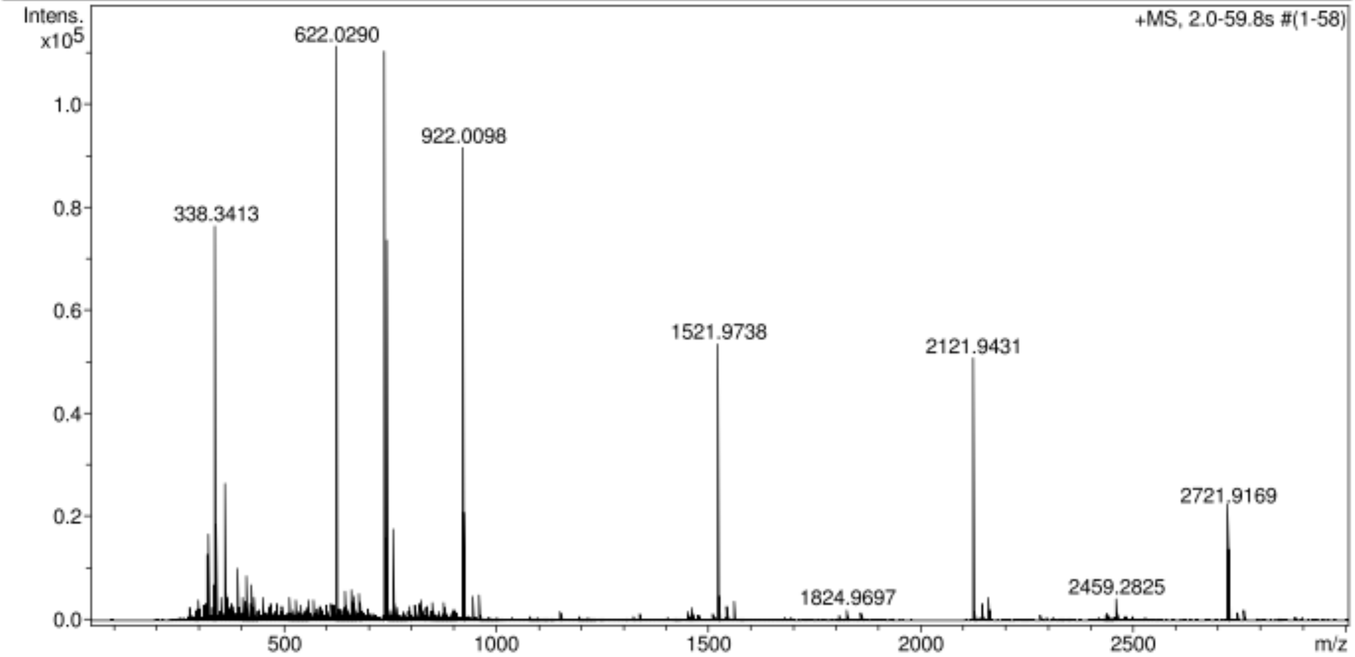

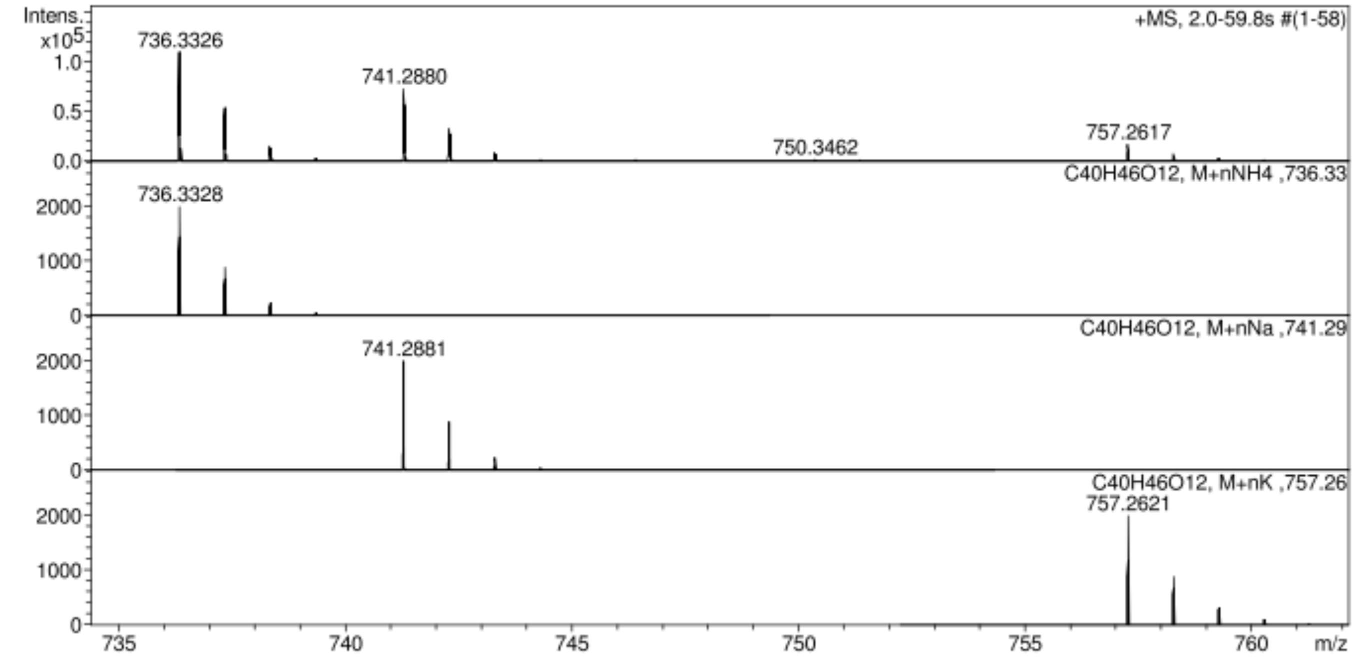

Propyl 2-O-(methyl 4-O-benzoyl-α-D-glucopyranosyluronate)-α-L-fucopyranoside (21a).

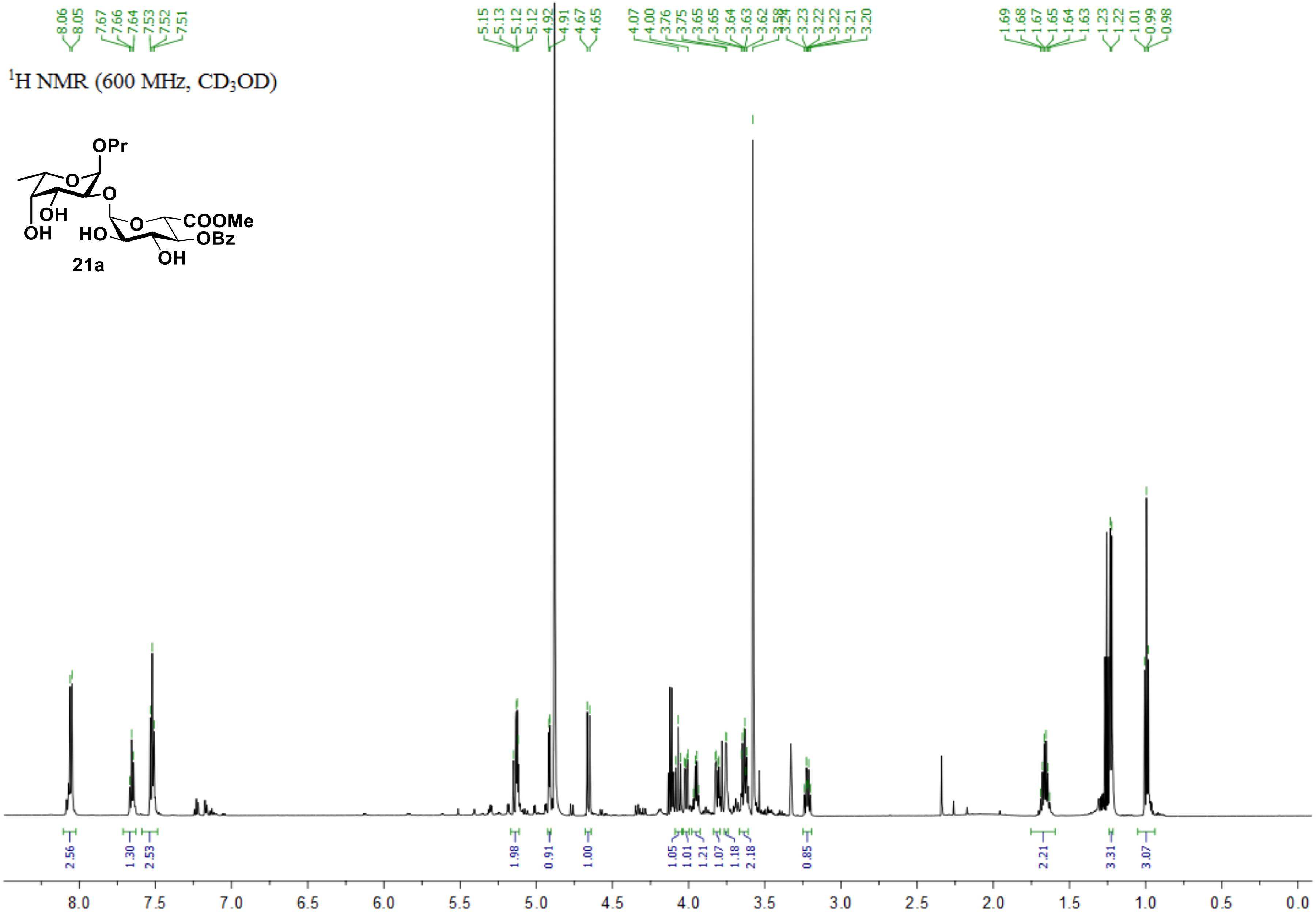

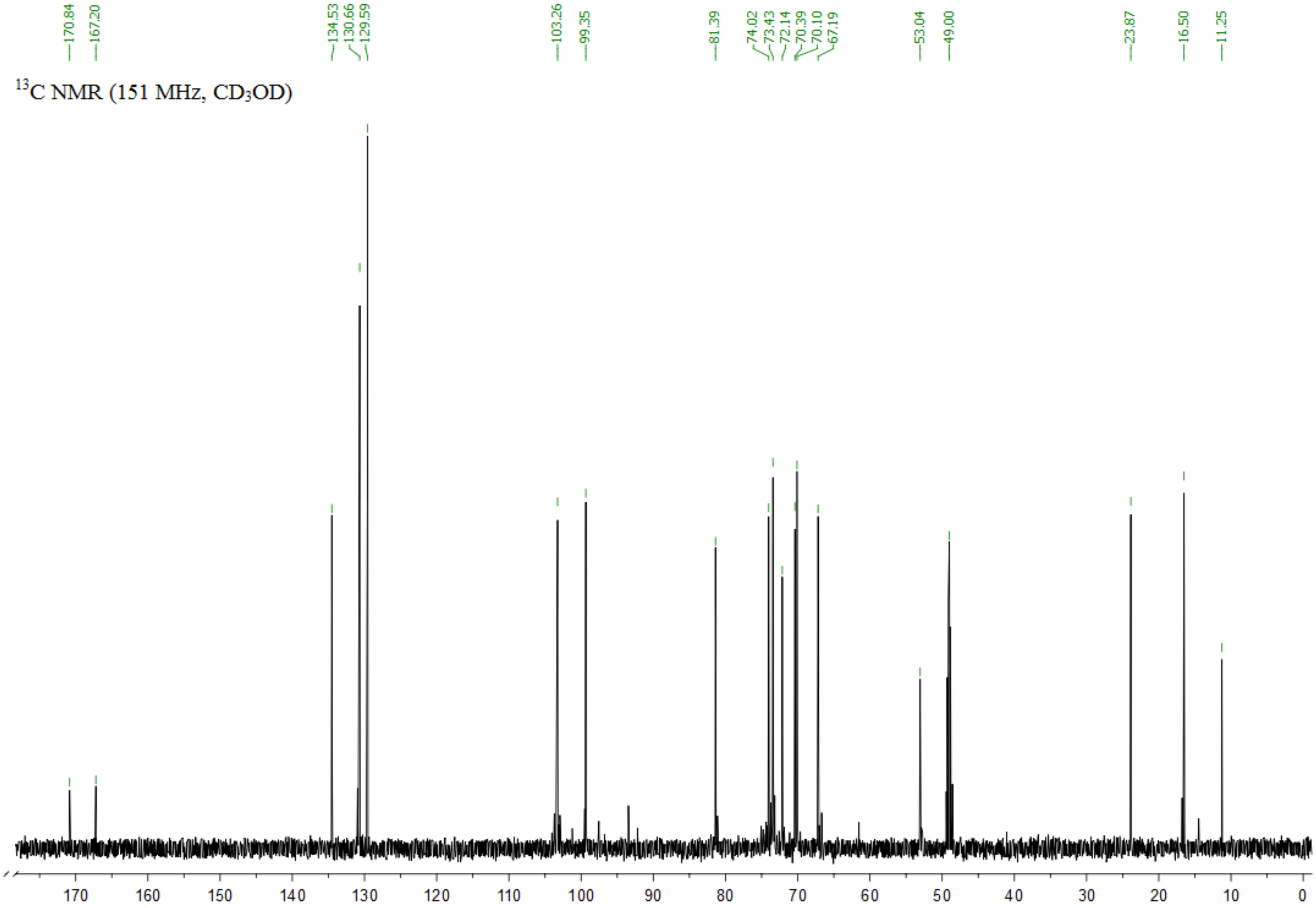

170.84
167.20
134.53
130.66
129.59
103.26
99.35
81.39
74.02
73.43
72.14
70.39
70.10
67.19
53.04
49.00
23.87
16.50
11.25
^{13}C NMR (151 MHz, CD_3OD)
170 160 150 140 130 120 110 100 90 80 70 60 50 40 30 20 10 0

Acquisition Parameter

Source Type	ESI	Ion Polarity	Positive	Set Nebulizer	0.5 Bar
Focus	Active			Set Dry Heater	180 °C
Scan Begin	50 m/z	Set Capillary	4500 V	Set Dry Gas	4.0 l/min
Scan End	3000 m/z	Set End Plate Offset	-500 V	Set Divert Valve	Waste

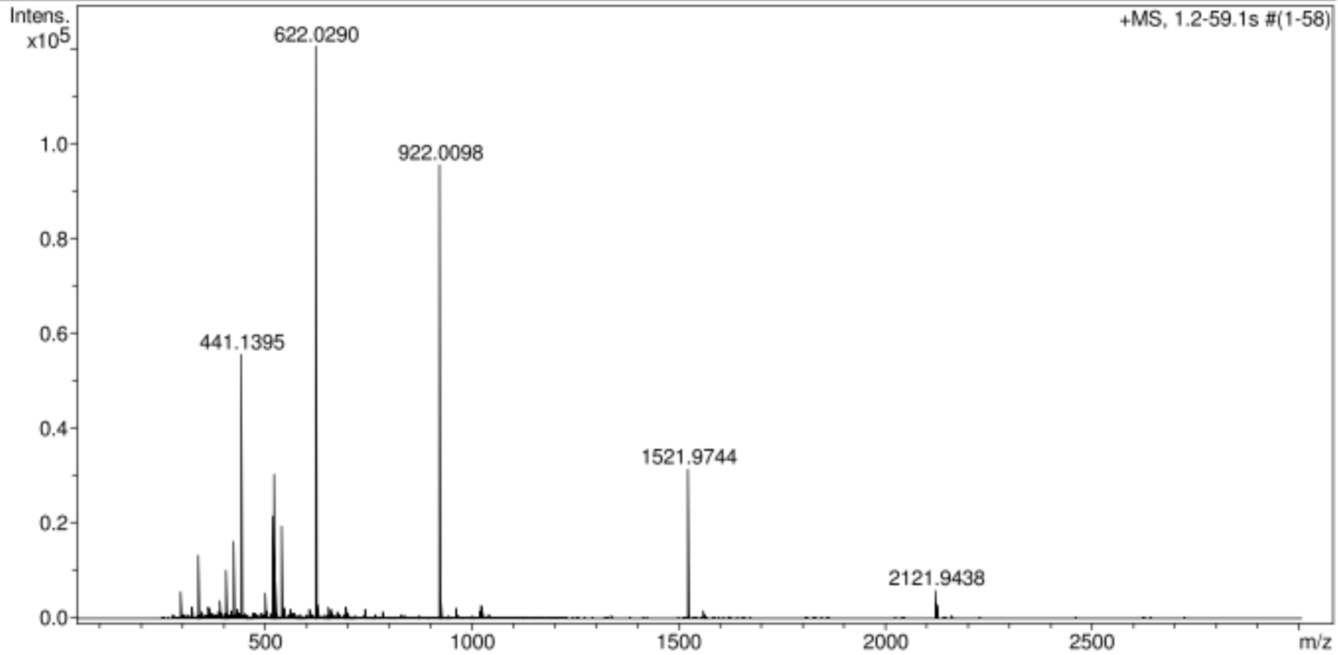

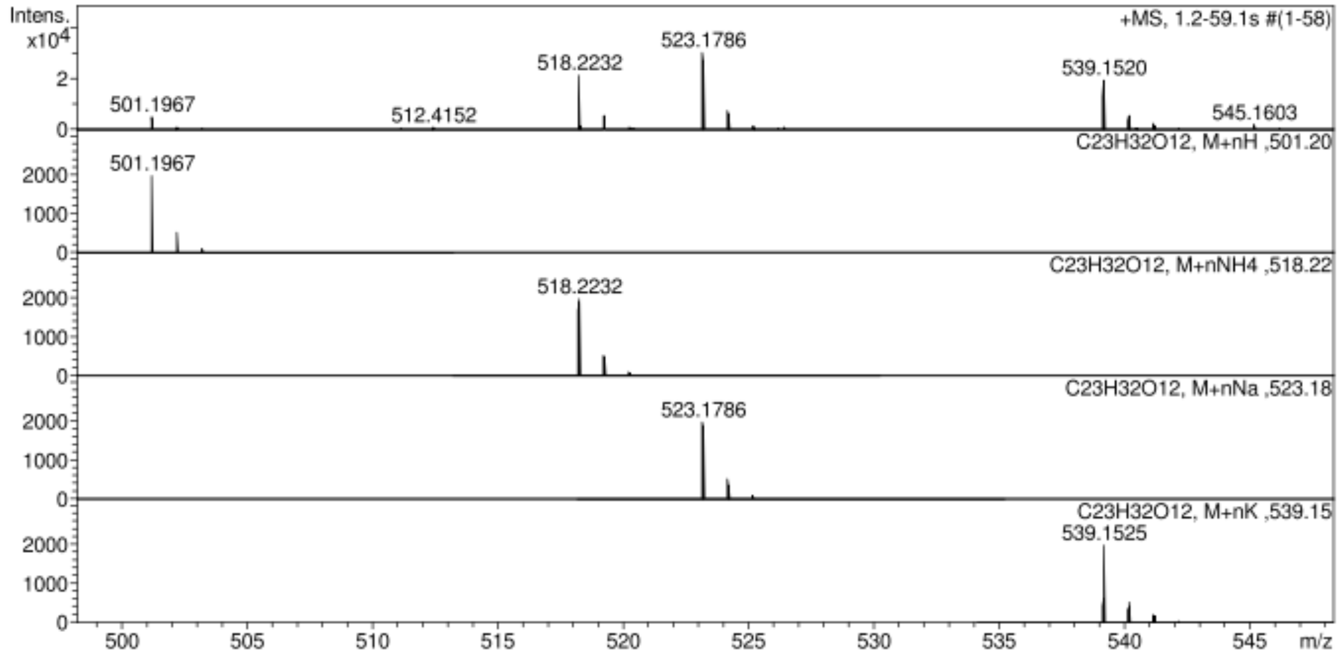

Propyl 2-O-(methyl 4-O-benzoyl-β-D-glucopyranosyluronate)-α-L-fucopyranoside (21b).

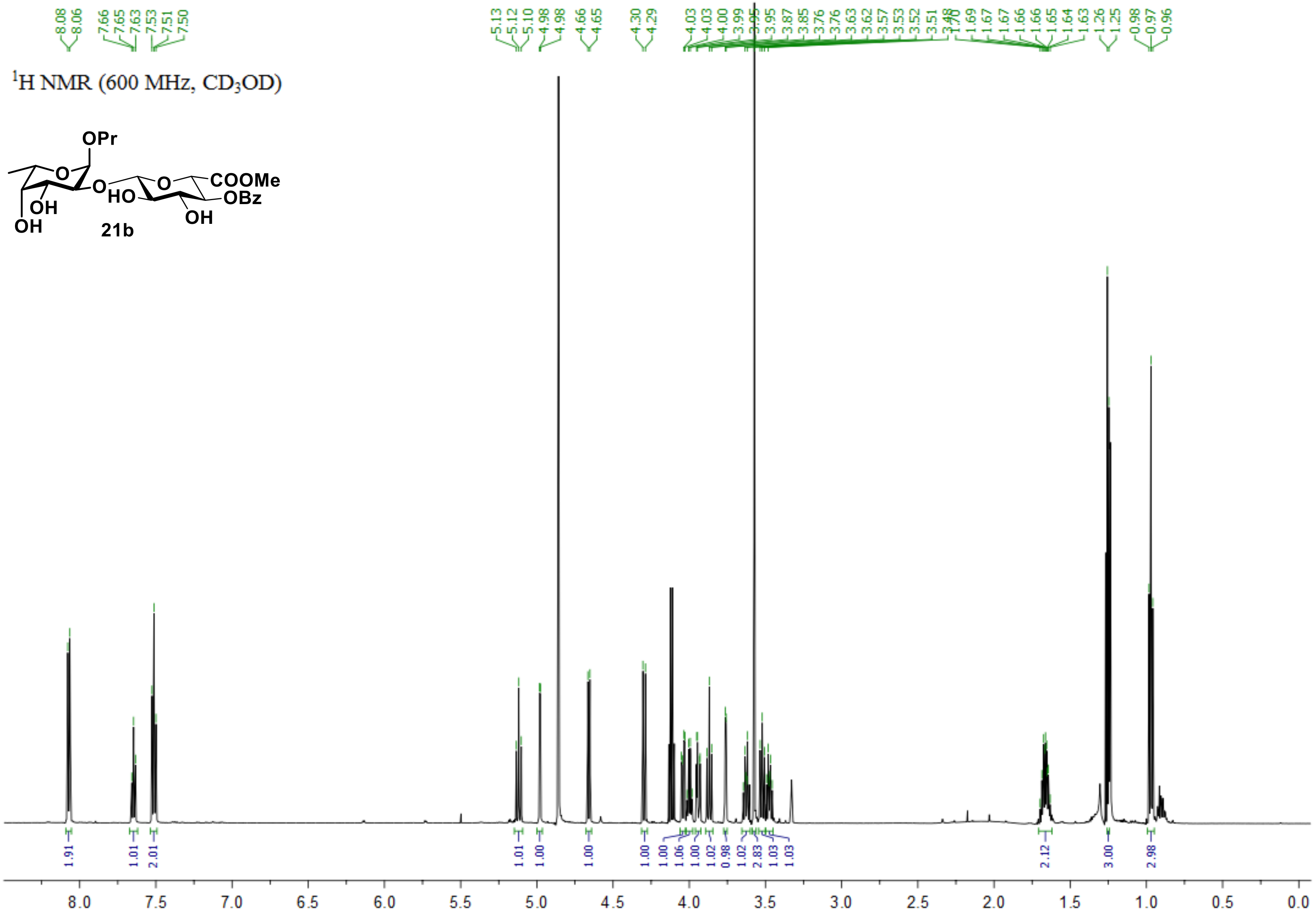

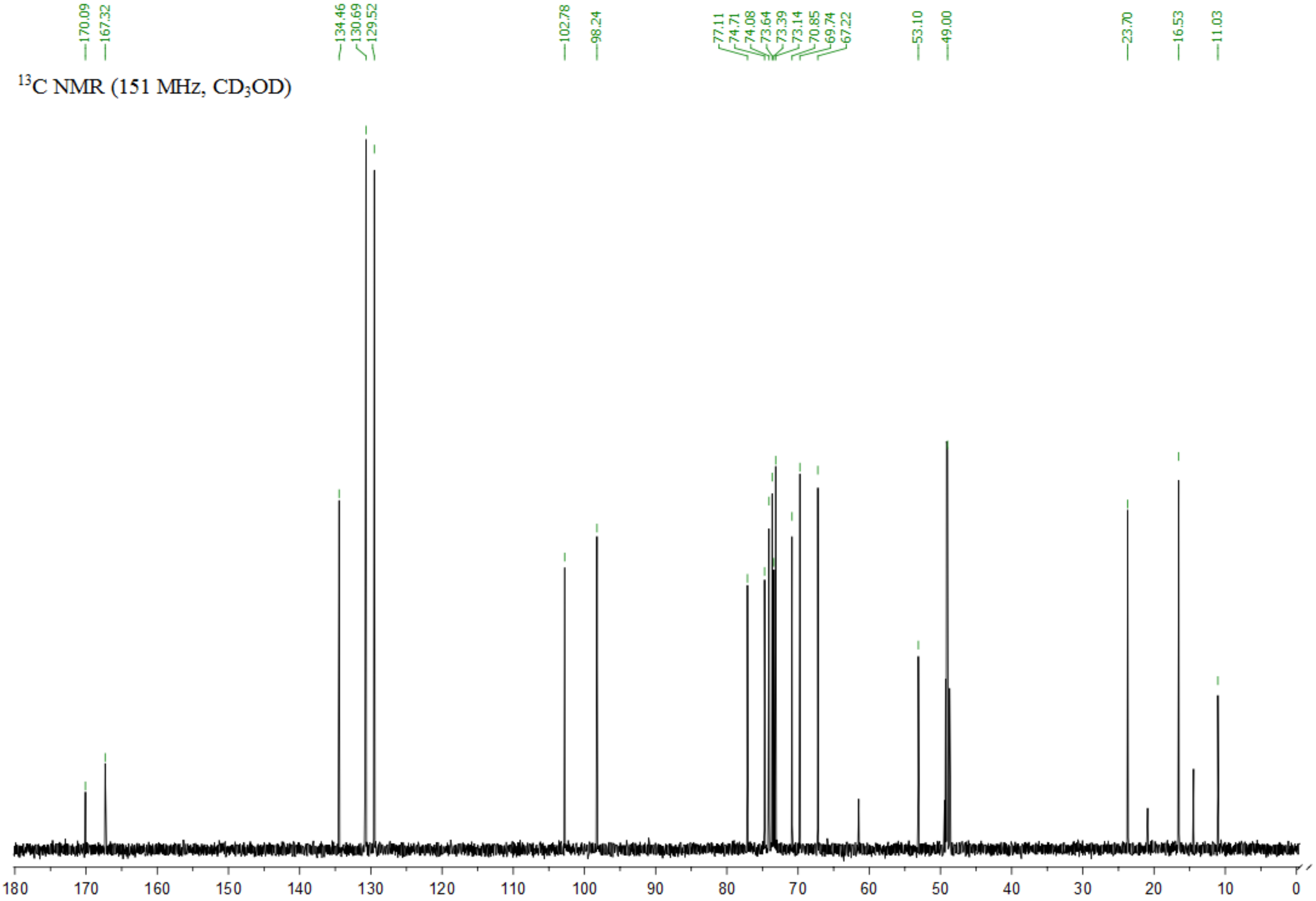
170.09
167.32
134.46
130.69
129.52
102.78
98.24
77.11
74.71
74.08
73.64
73.39
73.14
70.85
69.74
67.22
53.10
49.00
23.70
16.53
11.03
^{13}C NMR (151 MHz, CD_3OD)
180
170
160
150
140
130
120
110
100
90
80
70
60
50
40
30
20
10
0

Acquisition Parameter

Source Type	ESI	Ion Polarity	Positive	Set Nebulizer	0.5 Bar
Focus	Active			Set Dry Heater	180 °C
Scan Begin	50 m/z	Set Capillary	4500 V	Set Dry Gas	4.0 l/min
Scan End	3000 m/z	Set End Plate Offset	-500 V	Set Divert Valve	Waste

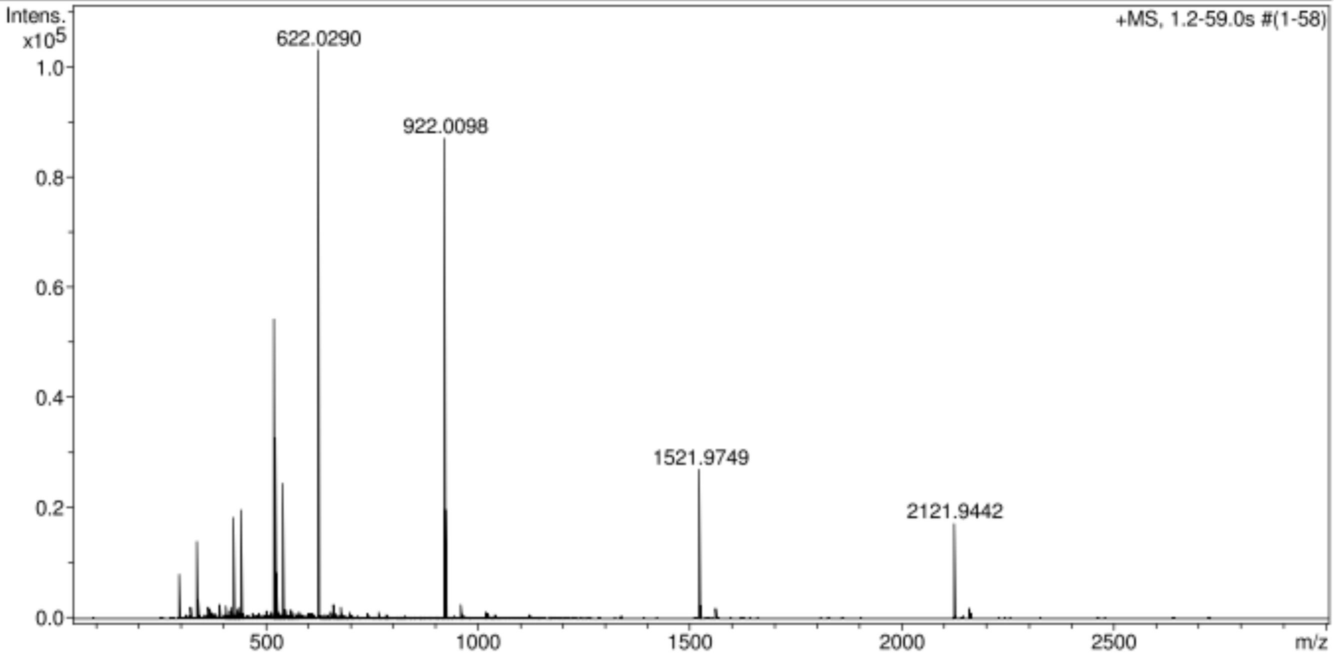

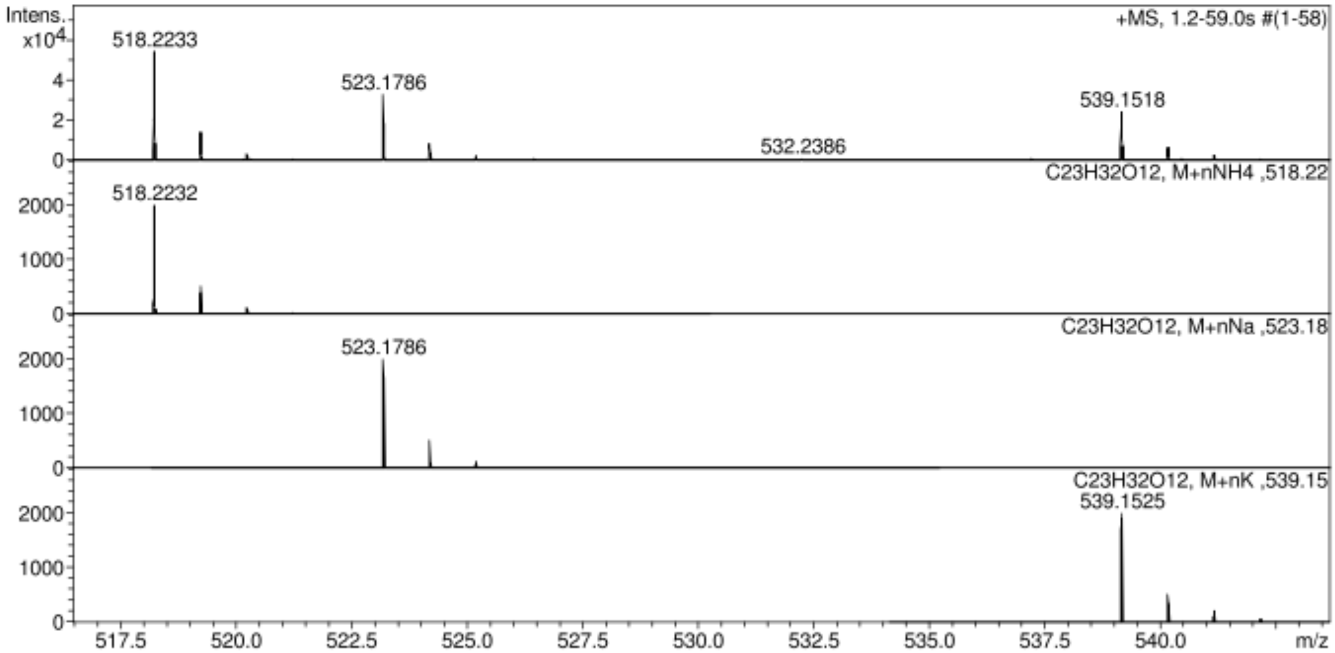

Propyl 2-O-(α-D-glucopyranosyluronic acid)-α-L-fucopyranoside sodium salt (1).

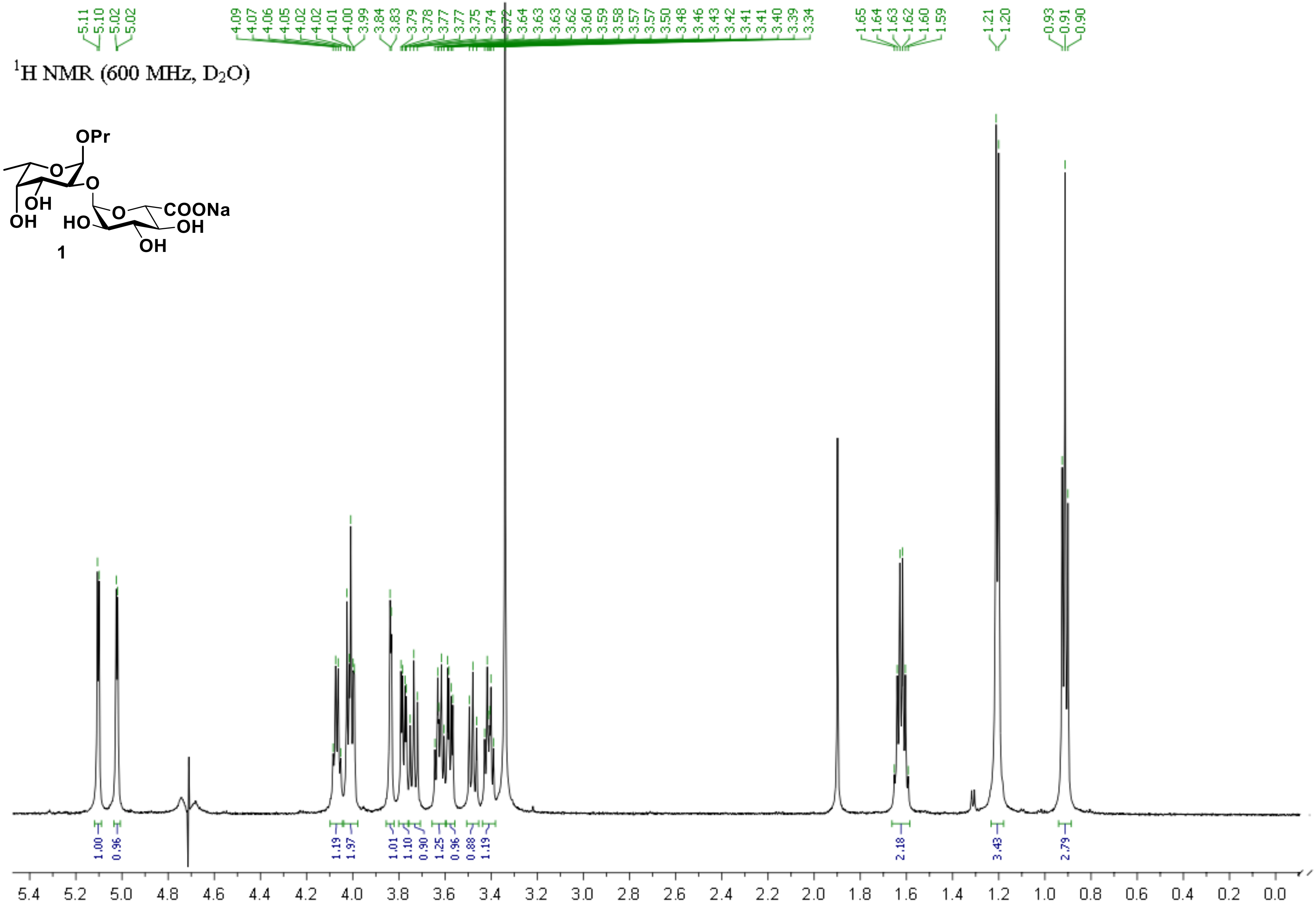

^{13}C NMR (151 MHz, D_2O)

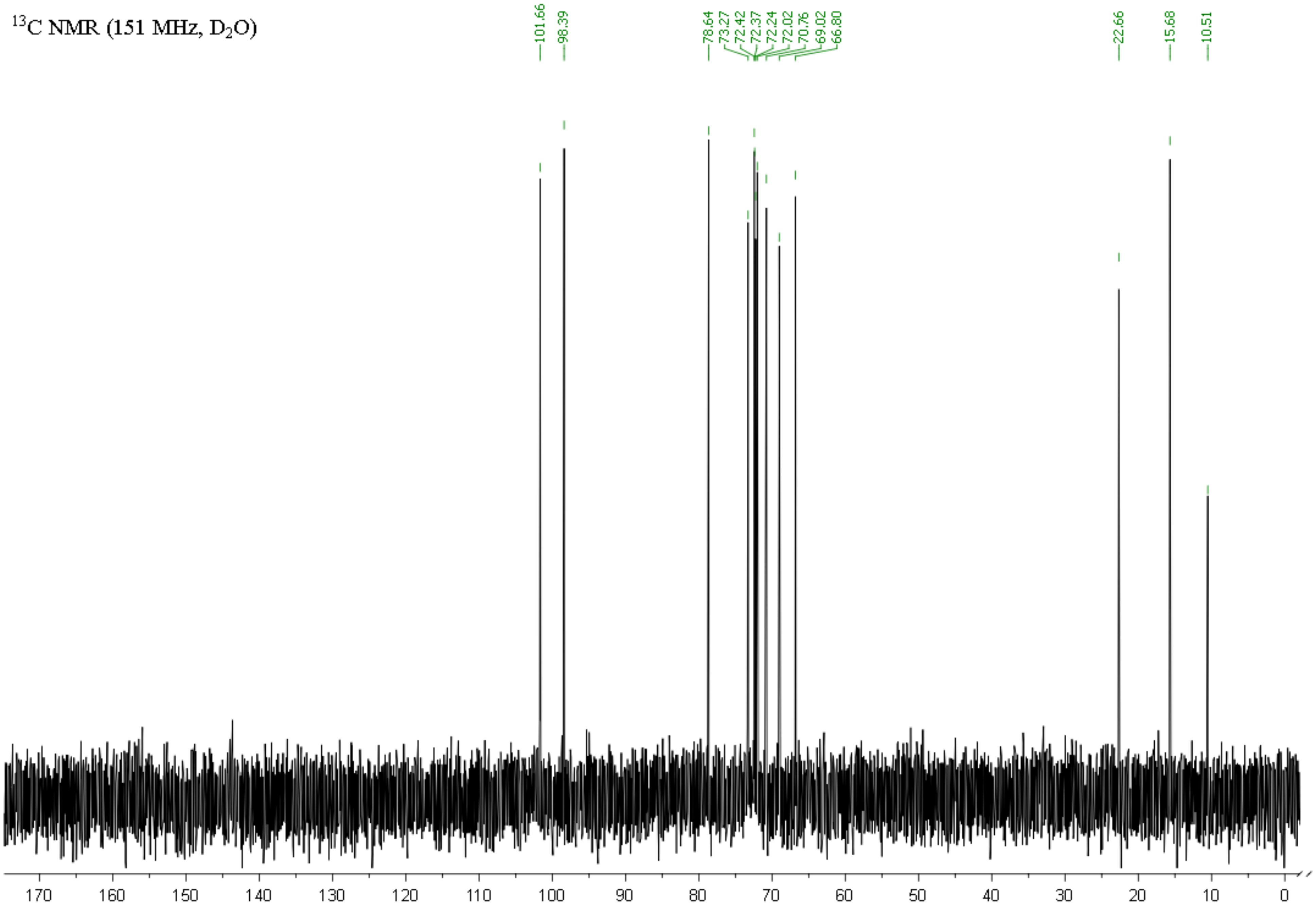

Acquisition Parameter

Source Type	ESI	Ion Polarity	Negative	Set Nebulizer	0.4 Bar
Focus	Active			Set Dry Heater	180 ℃
Scan Begin	50 m/z	Set Capillary	3000 V	Set Dry Gas	4.0 l/min
Scan End	3000 m/z	Set End Plate Offset	-500 V	Set Divert Valve	Waste

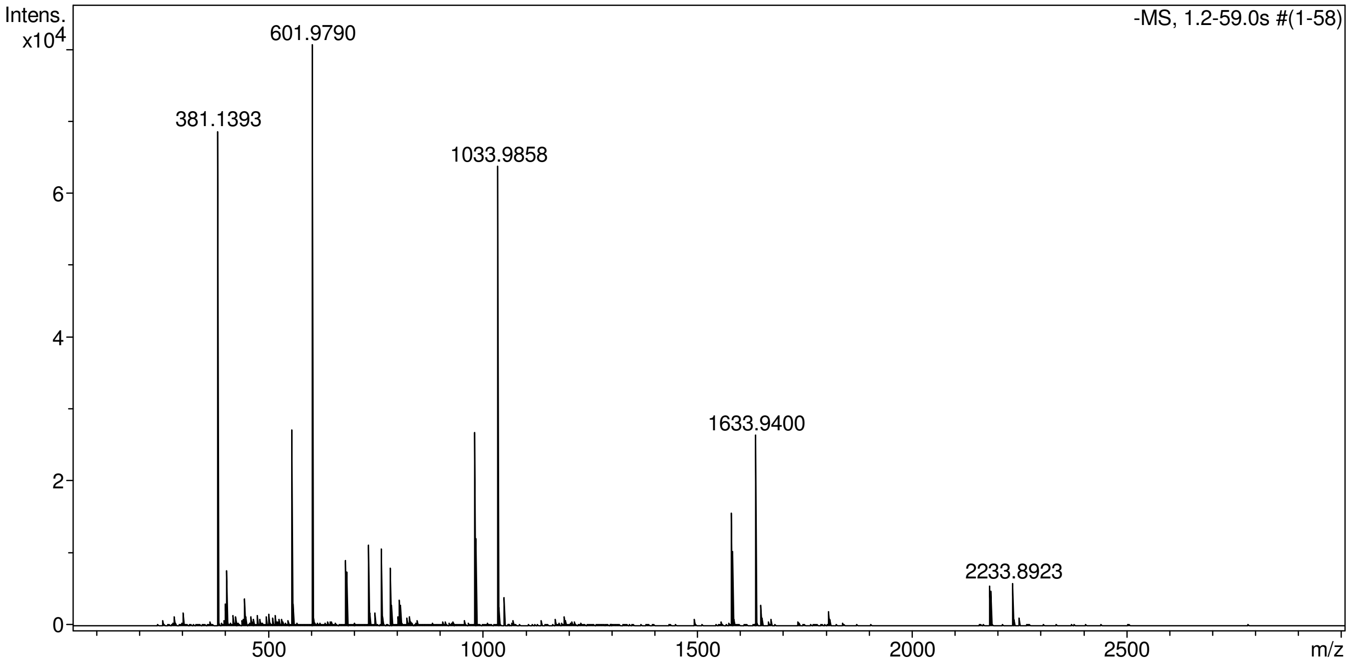

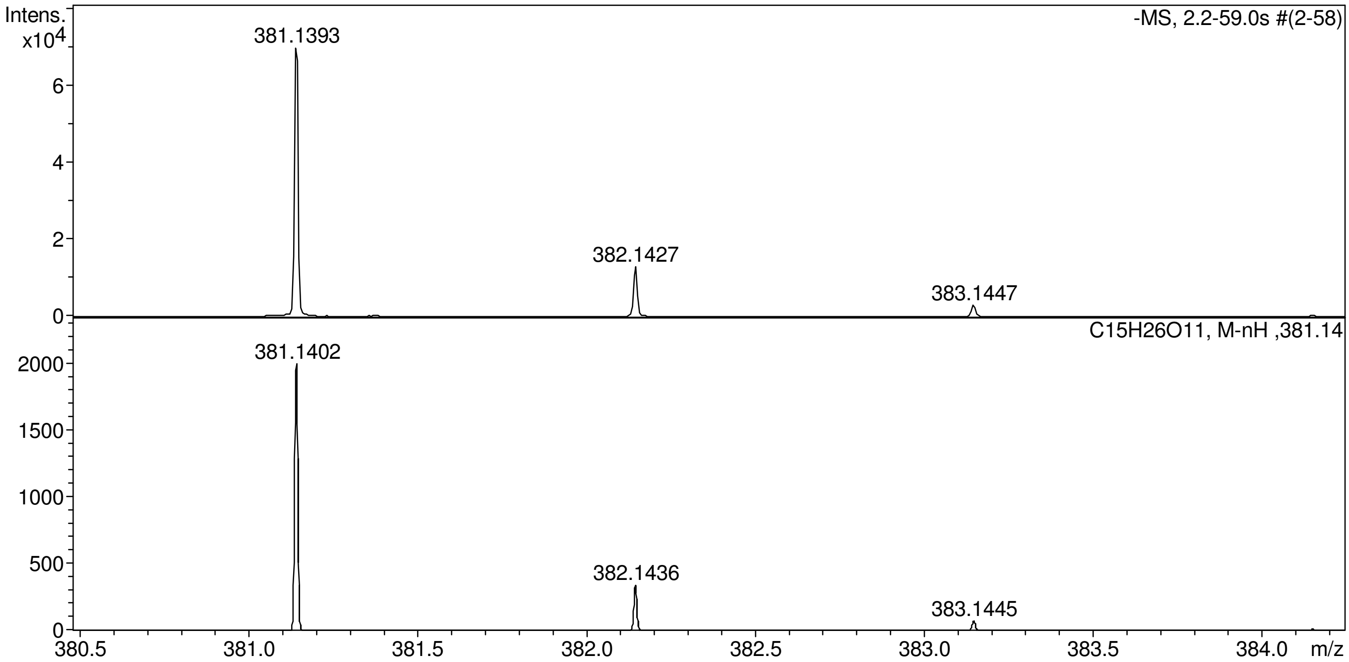

Propyl 2-O-(2,3-di-O-sulfo-α-D-glucopyranosyluronic acid)-3,4-di-O-sulfo-α-L-fucopyranoside sodium salt (2).

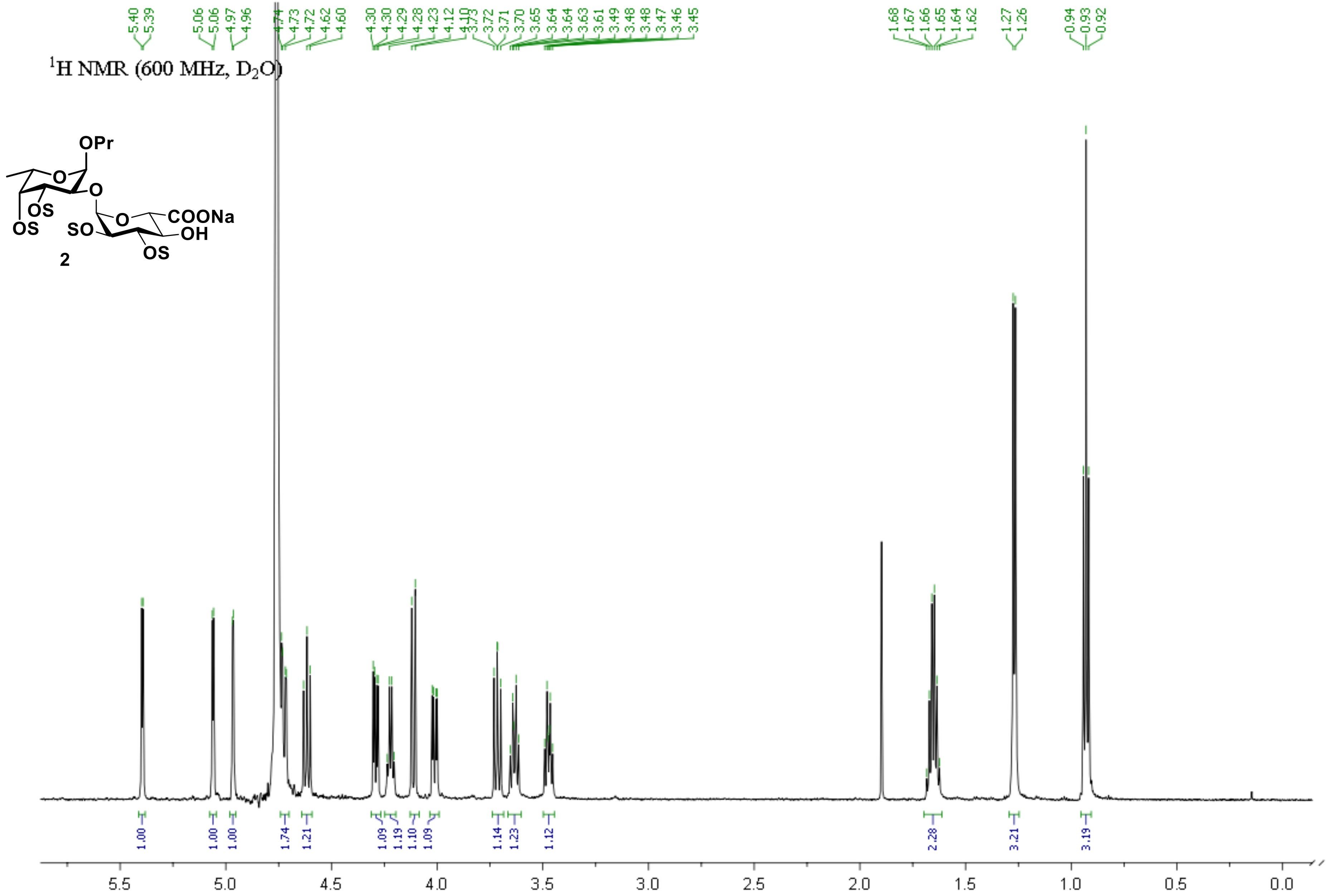

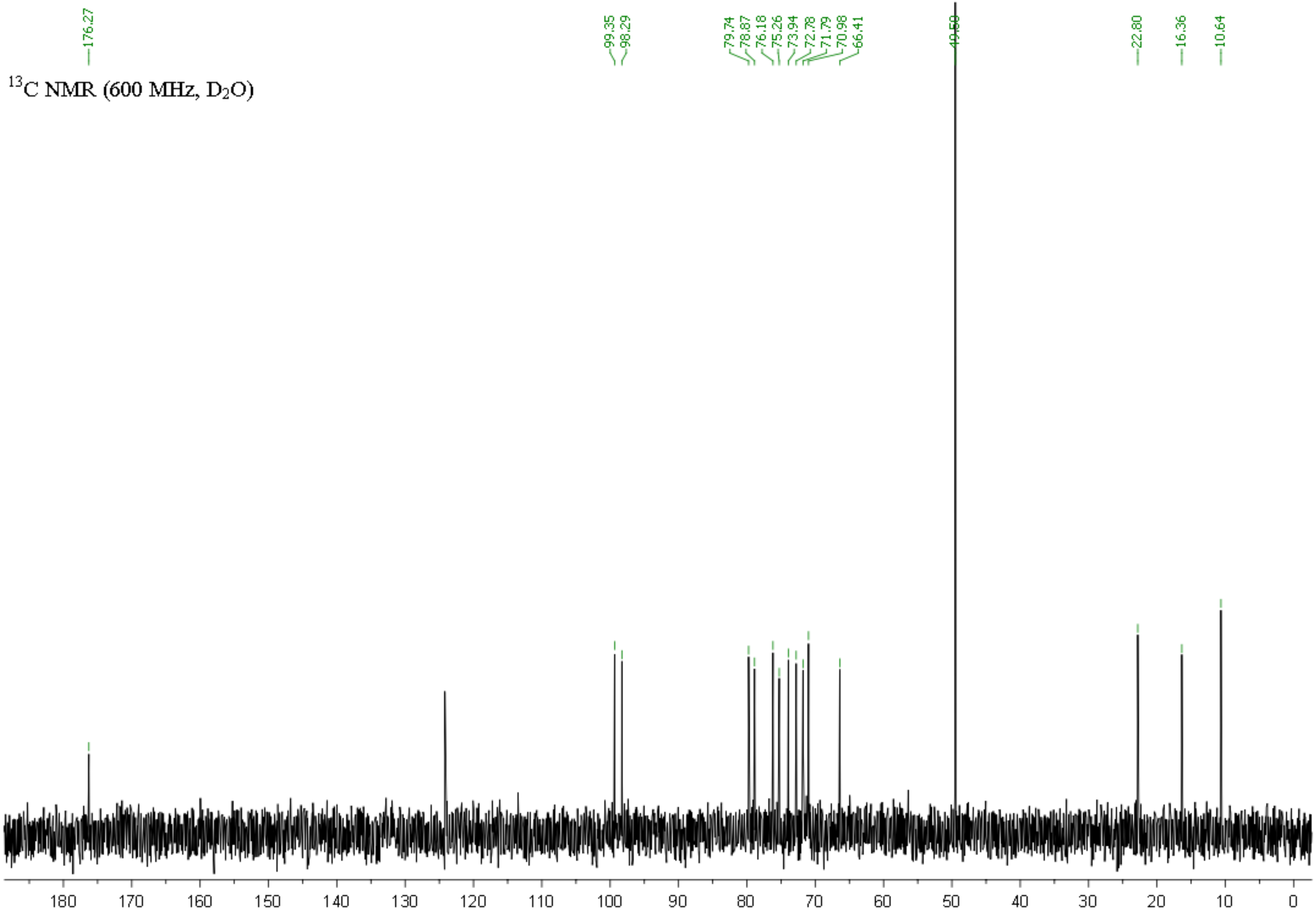

176.27
99.35
98.29
79.74
78.87
76.18
75.26
73.94
72.78
71.79
70.98
66.41
49.50
22.80
16.36
10.64
^{13}C NMR (600 MHz, D_2O)
180
170
160
150
140
130
120
110
100
90
80
70
60
50
40
30
20
10
0

Acquisition Parameter

Source Type	ESI	Ion Polarity	Negative	Set Nebulizer	0.4 Bar
Focus	Not active			Set Dry Heater	180 °C
Scan Begin	50 m/z	Set Capillary	3200 V	Set Dry Gas	4.0 l/min
Scan End	3000 m/z	Set End Plate Offset	-500 V	Set Divert Valve	Waste

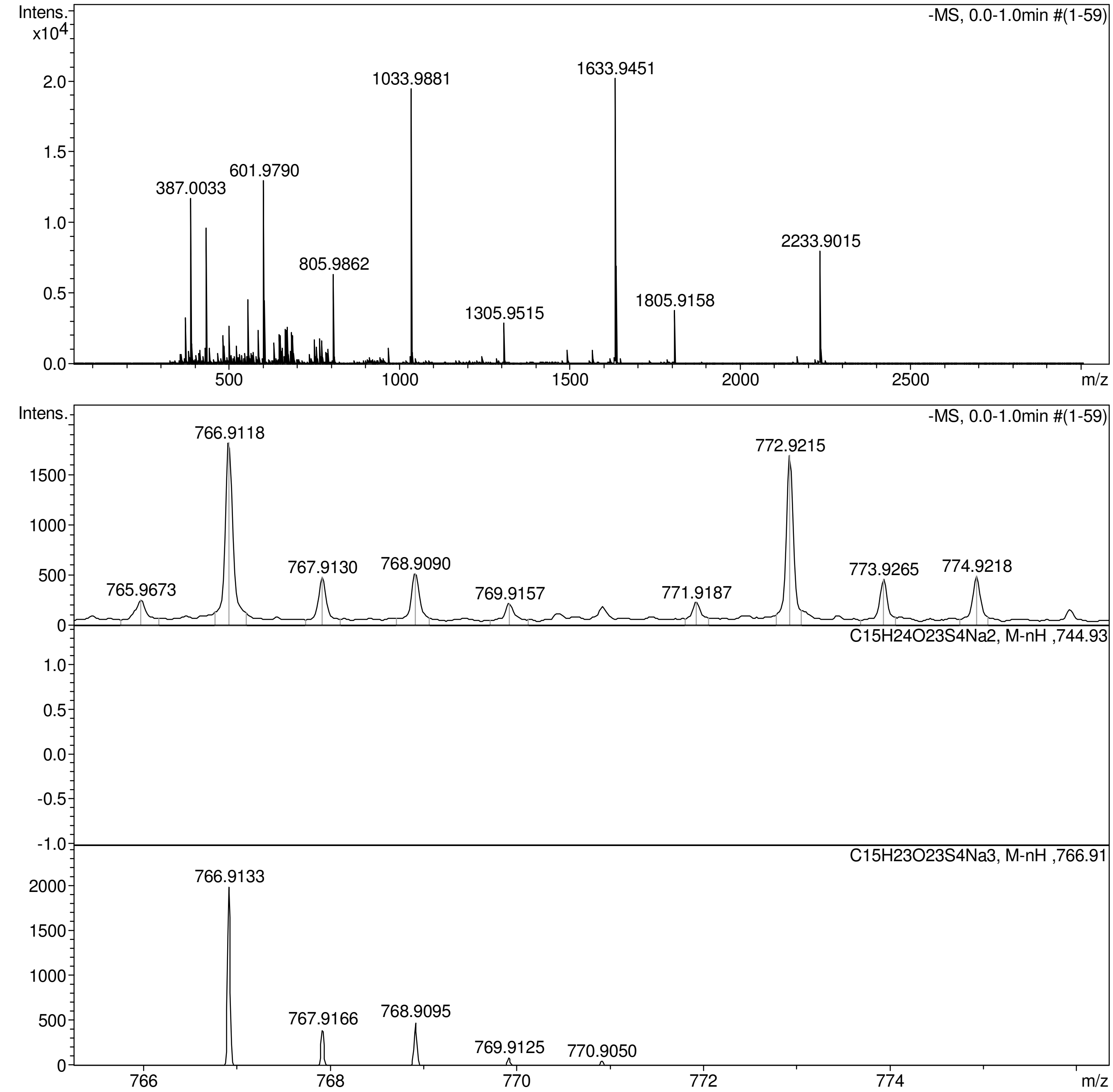

Propyl 2-O-(2,3,4-tri-O-sulfo-α-D-glucopyranosyluronic acid)-3,4-di-O-sulfo-α-L-fucopyranoside sodium salt (3).

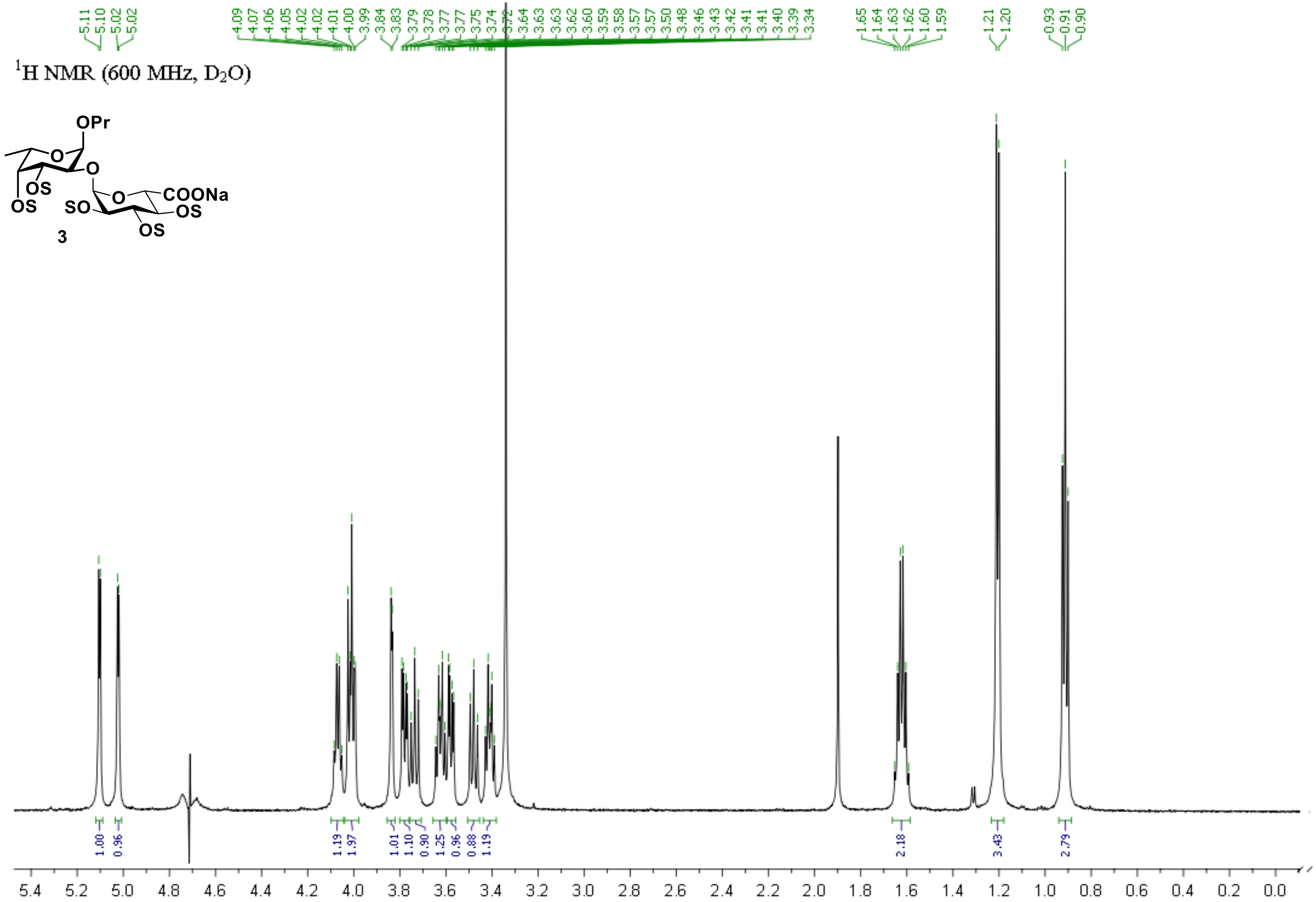

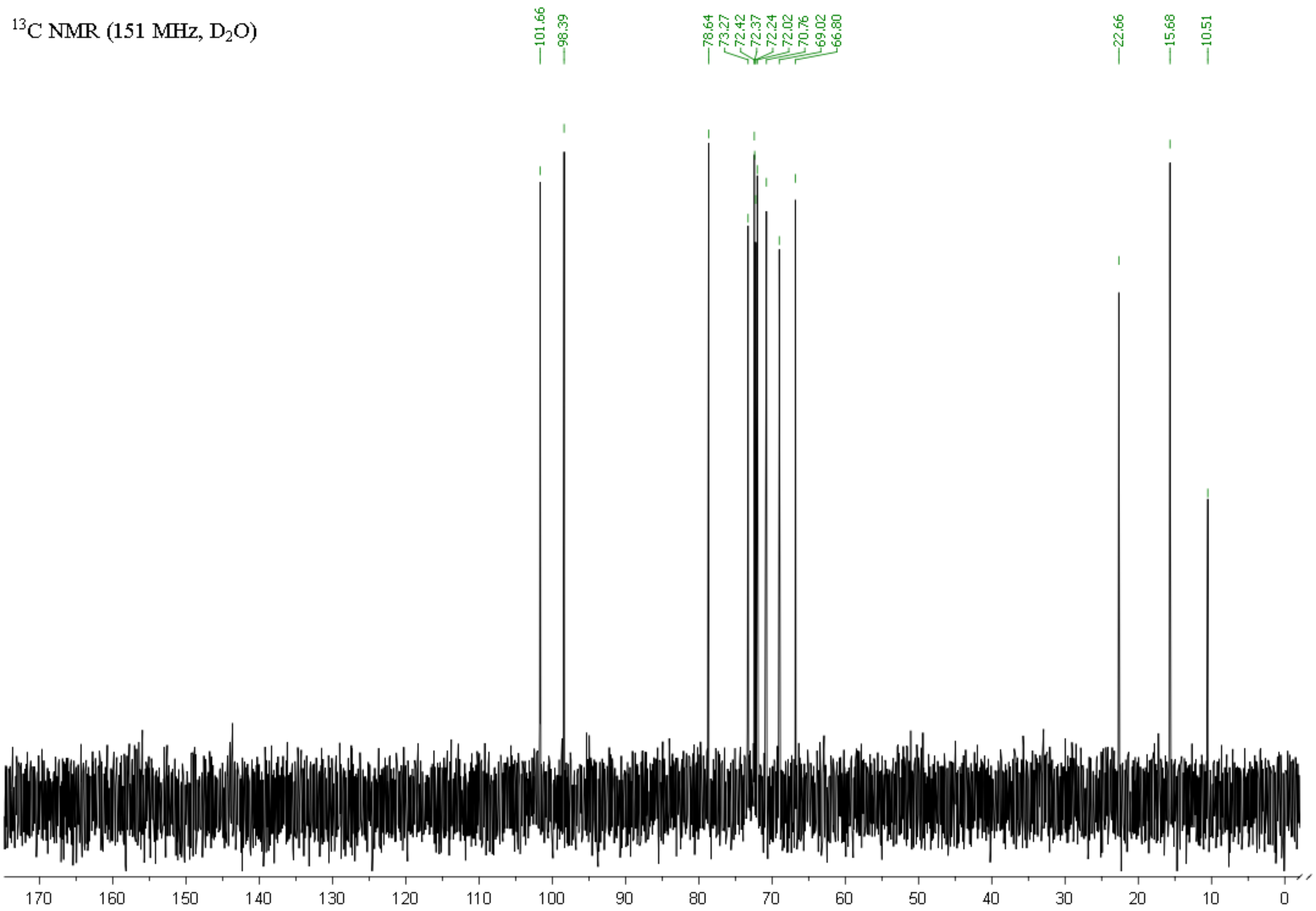

13C NMR (151 MHz, D2O)
101.66
98.39
78.64
73.27
72.42
72.37
72.24
72.02
70.76
69.02
66.80
22.66
15.68
10.51
170 160 150 140 130 120 110 100 90 80 70 60 50 40 30 20 10 0

Acquisition Parameter

Source Type	ESI	Ion Polarity	Negative	Set Nebulizer	0.4 Bar
Focus	Not active			Set Dry Heater	180 °C
Scan Begin	50 m/z	Set Capillary	3200 V	Set Dry Gas	4.0 l/min
Scan End	3000 m/z	Set End Plate Offset	-500 V	Set Divert Valve	Waste

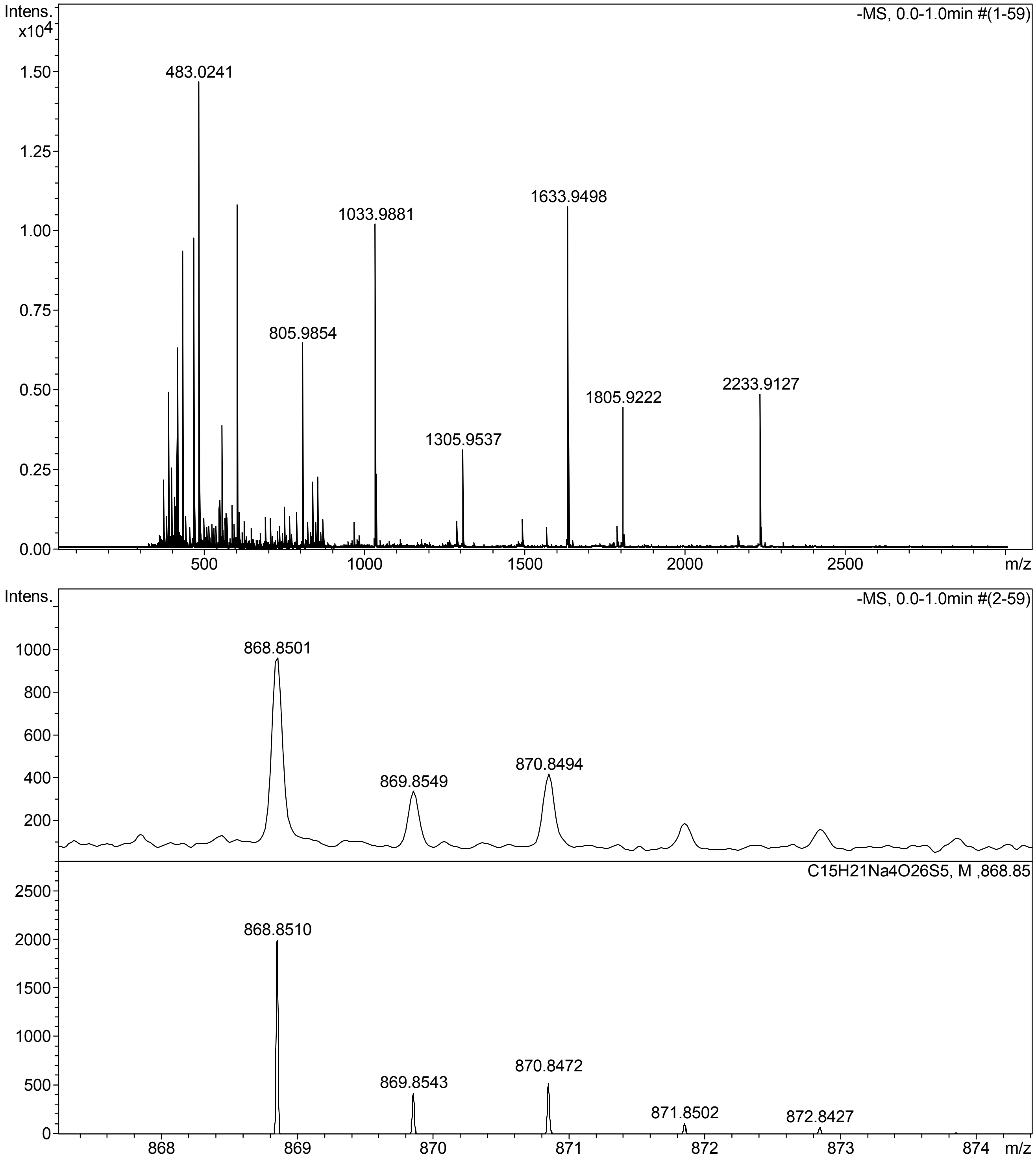

Propyl 2-O-(β-D-glucopyranosyluronic acid)-α-L-fucopyranoside sodium salt (4).

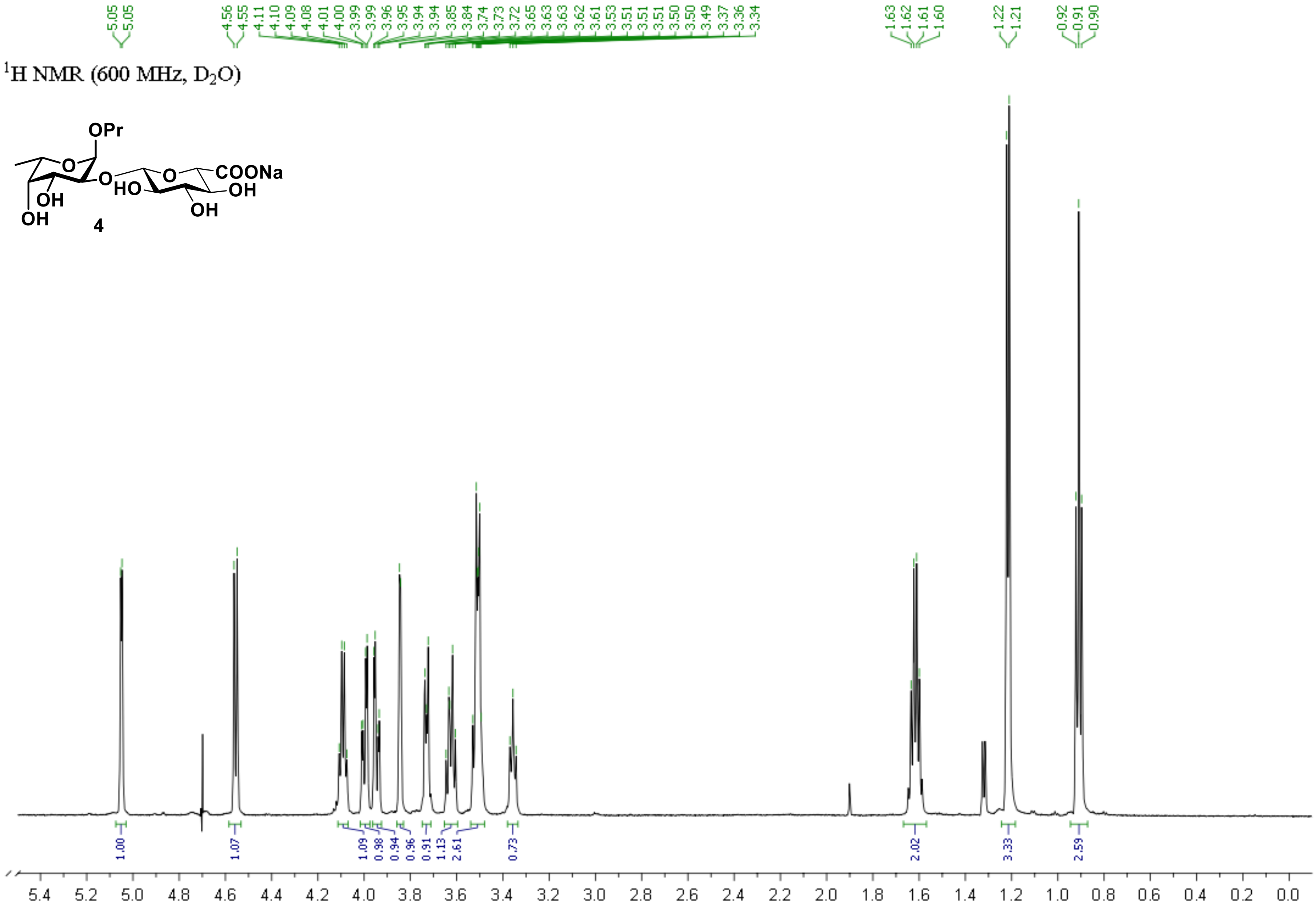

^{13}C NMR (150 MHz, D_2O)

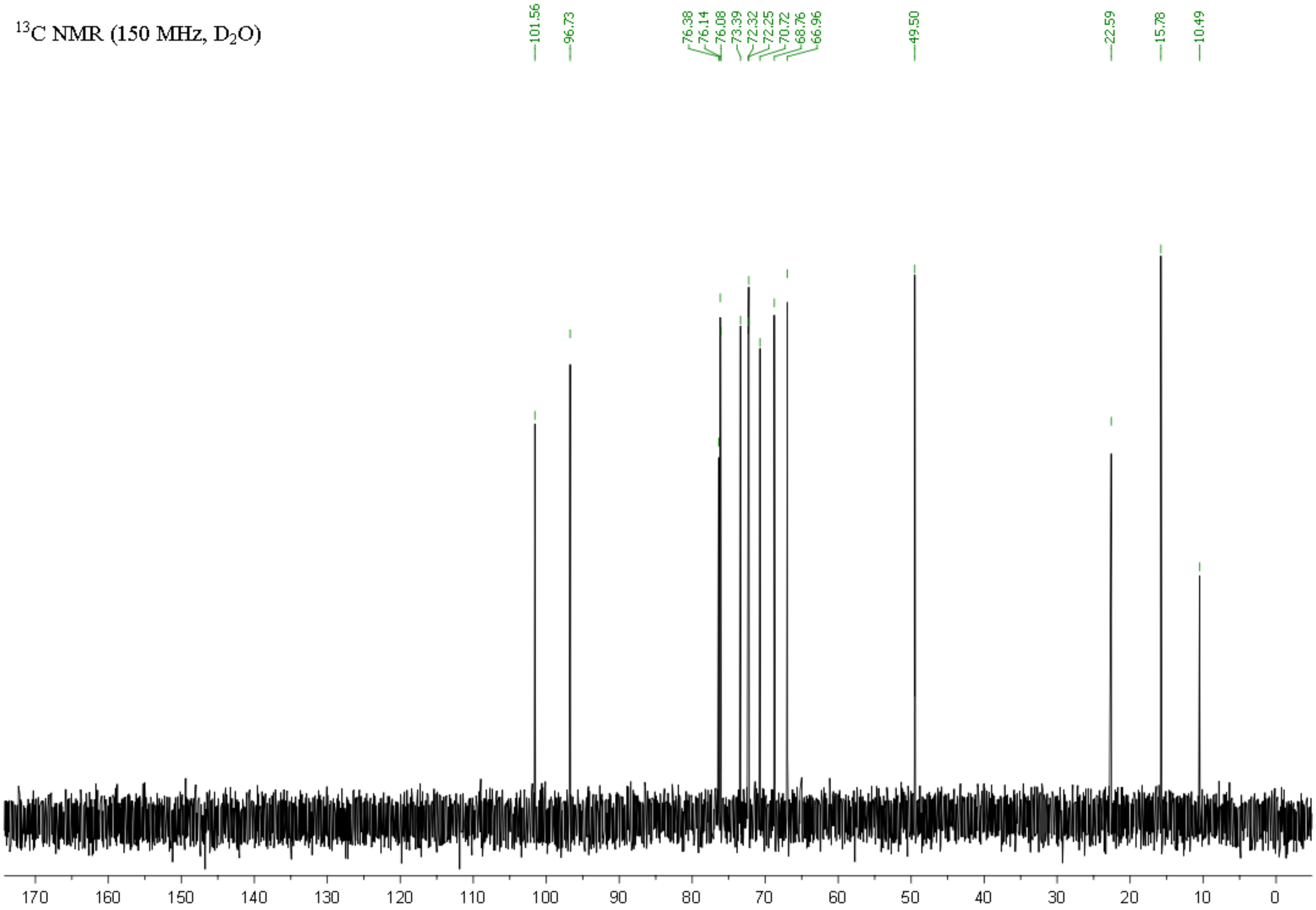

NOESY NMR (D_2O)

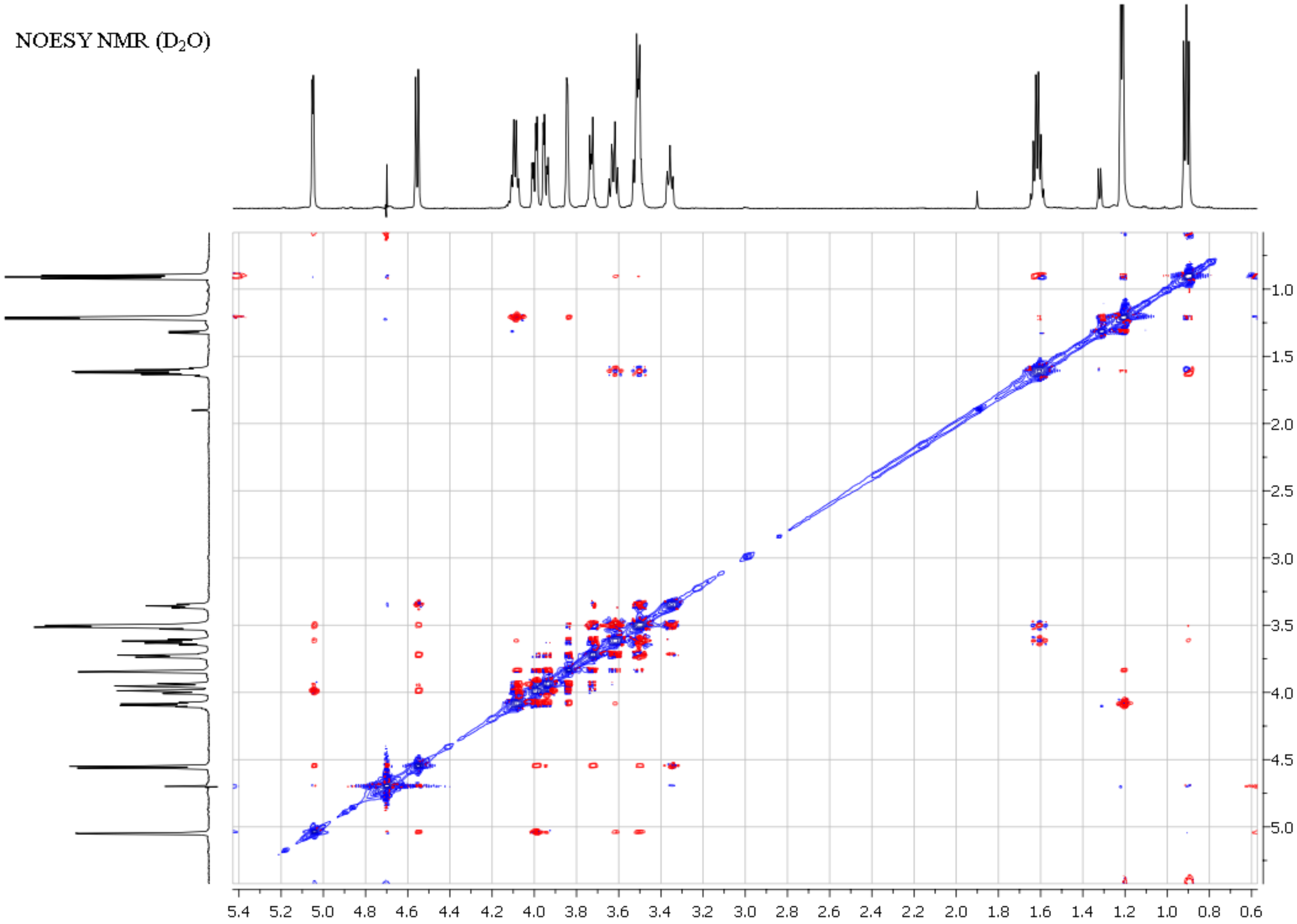

Acquisition Parameter

Source Type	ESI	Ion Polarity	Negative	Set Nebulizer	0.4 Bar
Focus	Not active			Set Dry Heater	180 °C
Scan Begin	50 m/z	Set Capillary	2000 V	Set Dry Gas	4.0 l/min
Scan End	1050 m/z	Set End Plate Offset	-500 V	Set Divert Valve	Source

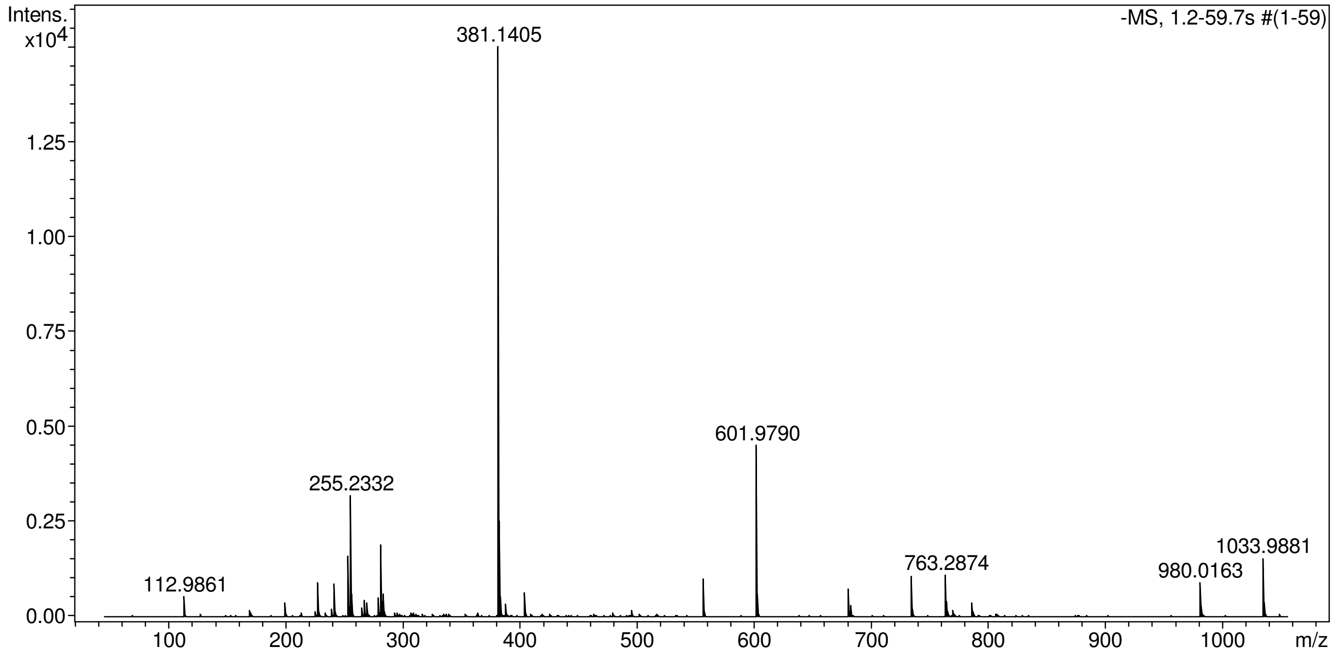

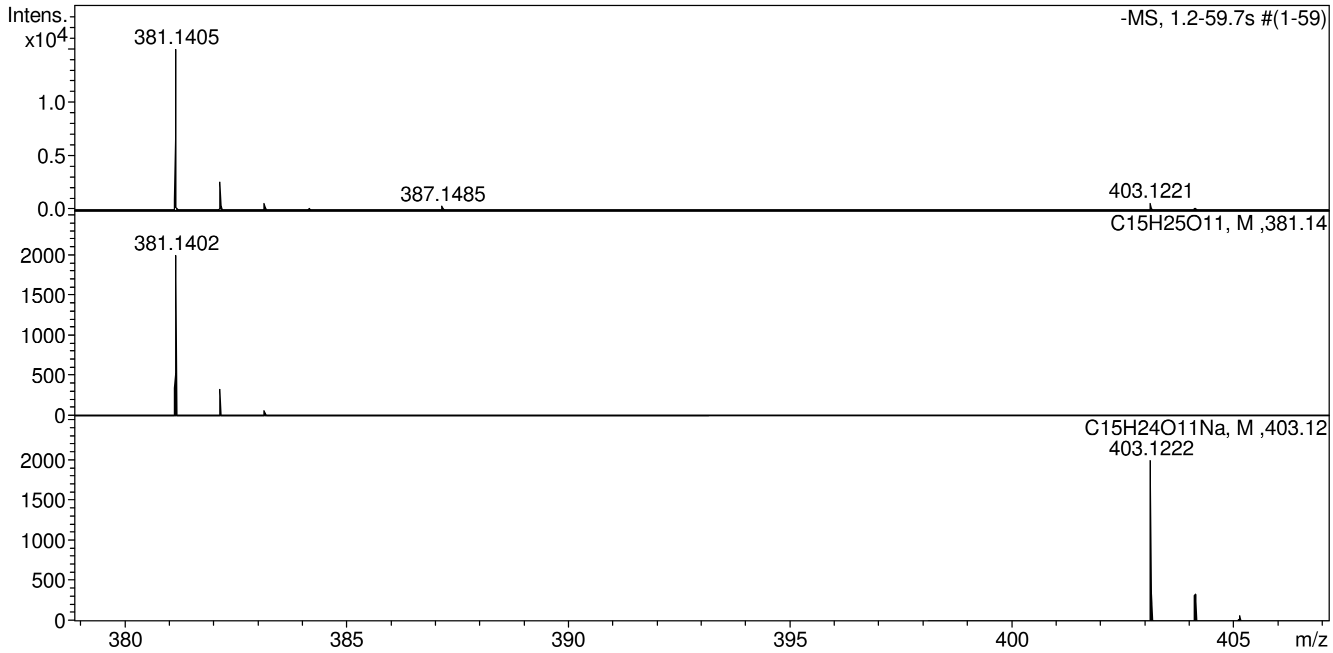

Propyl 2-O-(2,3-di-O-sulfo-β-D-glucopyranosyluronic acid)-3,4-di-O-sulfo-α-L-fucopyranoside sodium salt (5).

^{1}H NMR (600 MHz, D_2O)

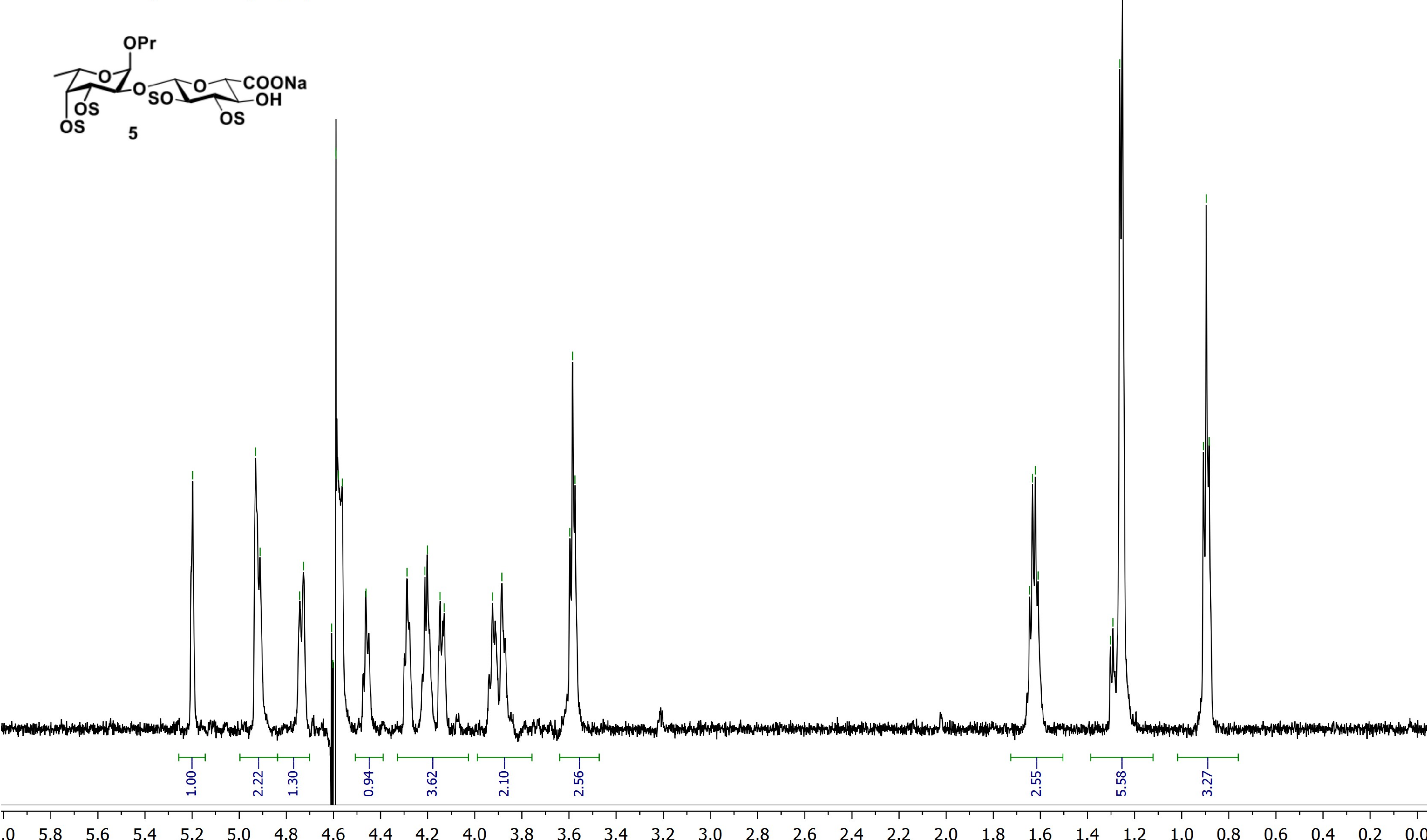

^{13}C NMR (151 MHz, D_2O)

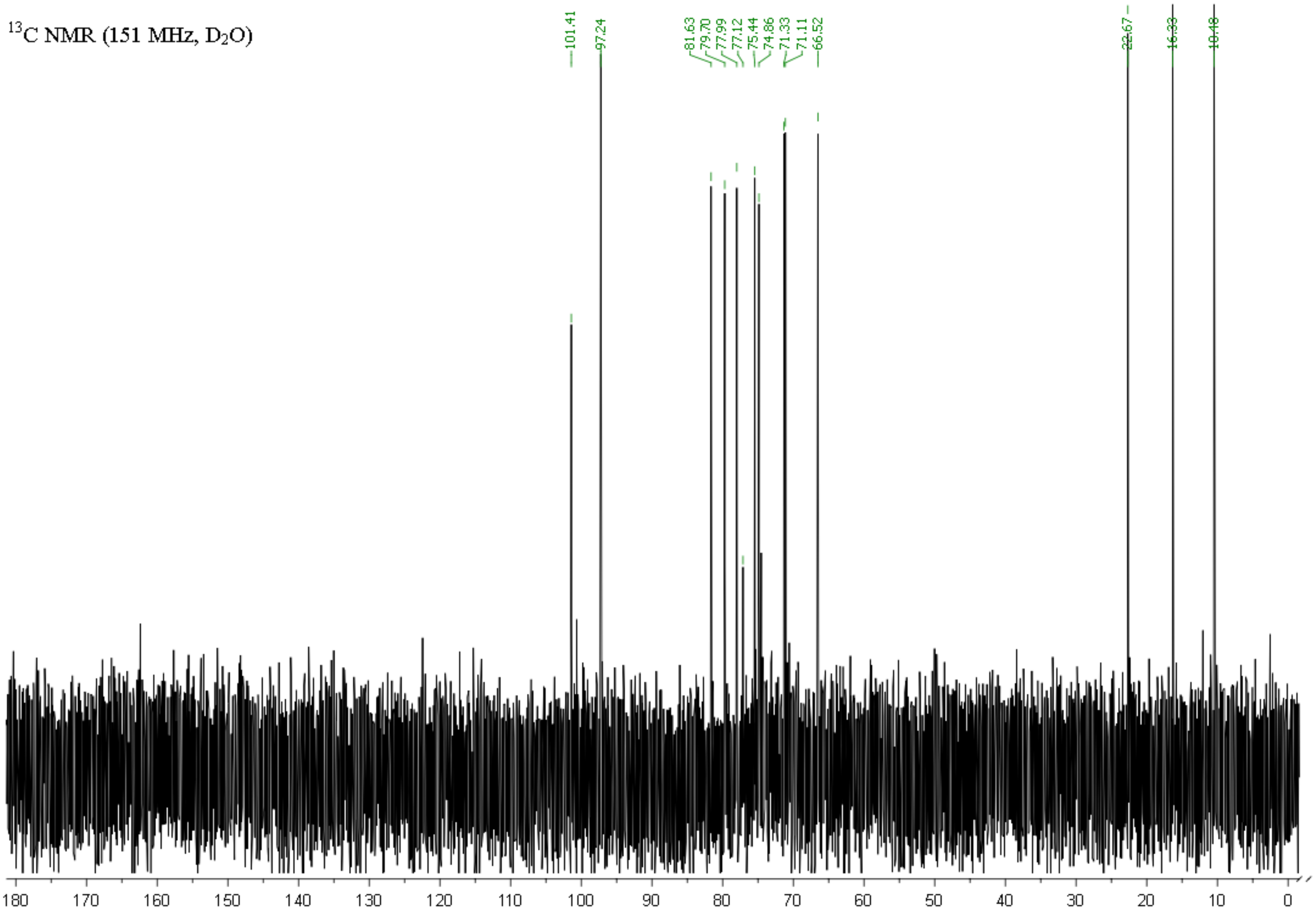

Acquisition Parameter

Source Type	ESI	Ion Polarity	Negative	Set Nebulizer	0.4 Bar
Focus	Not active			Set Dry Heater	180 °C
Scan Begin	50 m/z	Set Capillary	3200 V	Set Dry Gas	4.0 l/min
Scan End	3000 m/z	Set End Plate Offset	-500 V	Set Divert Valve	Waste

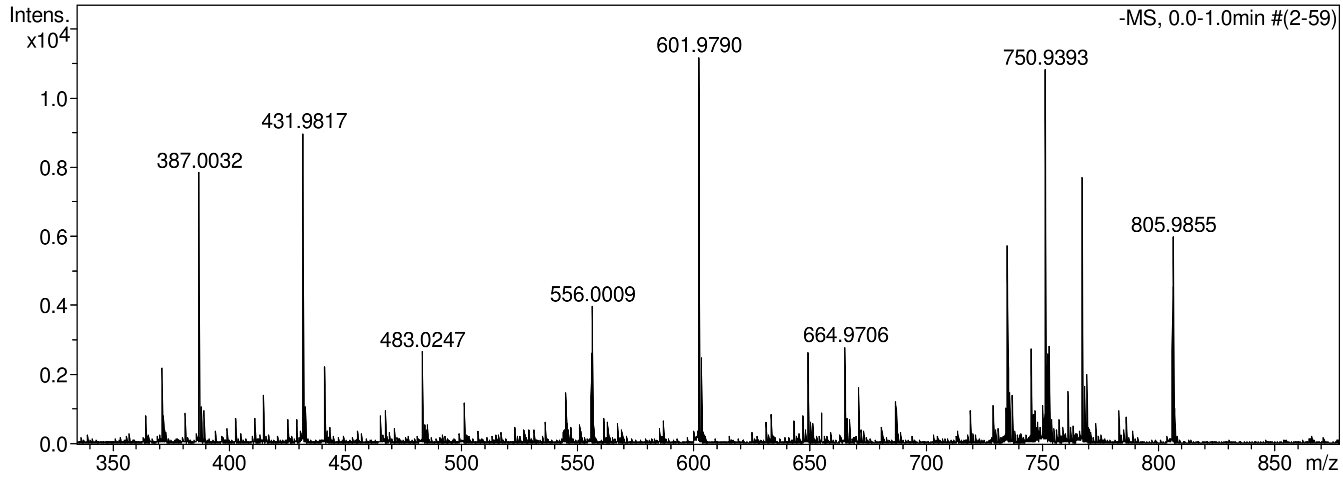

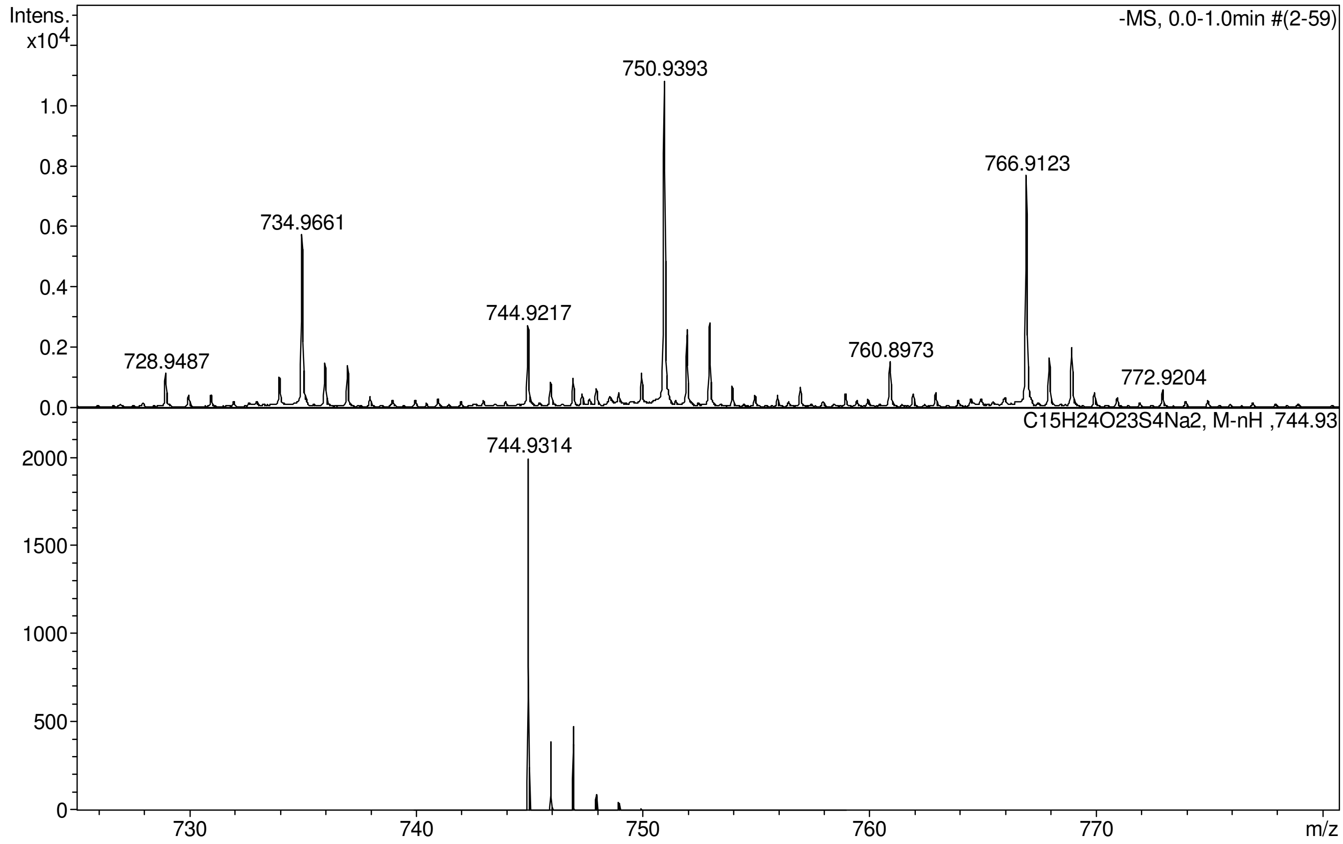

Propyl 2-O-(2,3,4-tri-O-sulfo-β-D-glucopyranosyluronic acid)-3,4-di-O-sulfo-α-L-fucopyranoside sodium salt (6).

^{1}H NMR (600 MHz, D_2O)

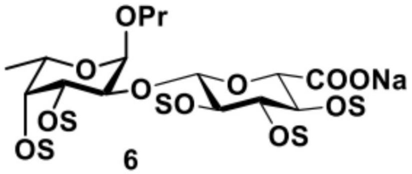

6

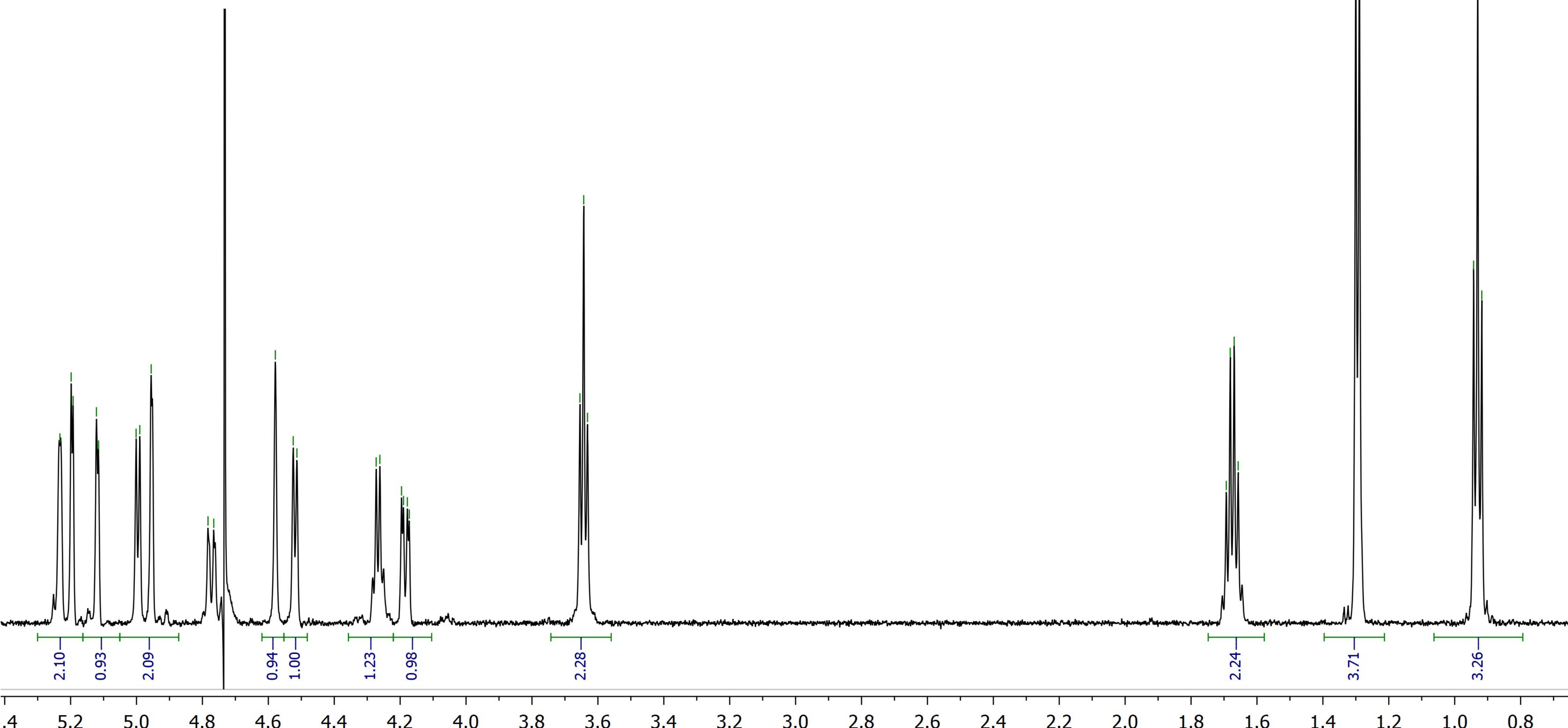

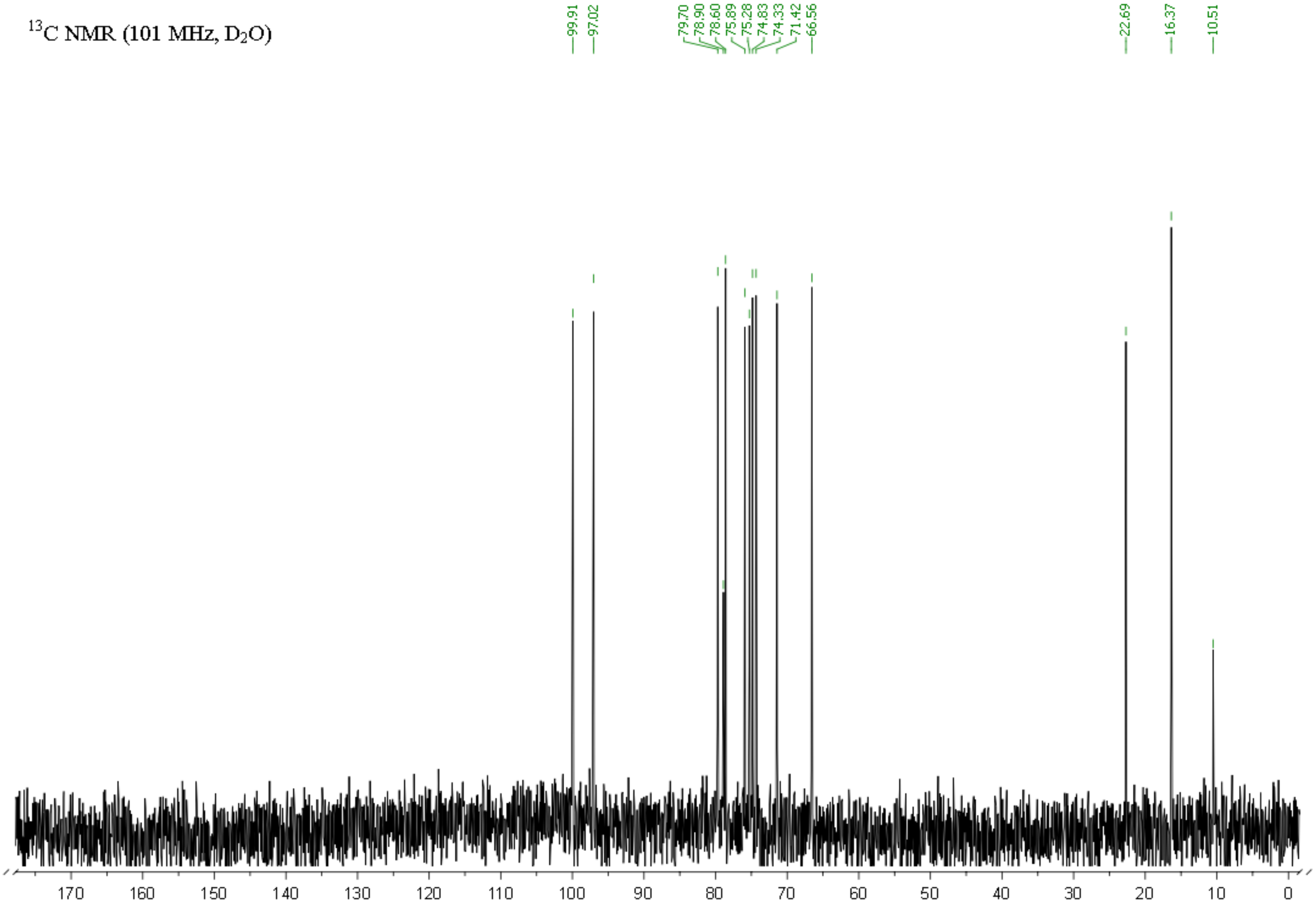

^{13}C NMR (101 MHz, D_2O)
99.91
97.02
79.70
78.90
78.60
75.89
75.28
74.83
74.33
71.42
66.56
22.69
16.37
10.51
170 160 150 140 130 120 110 100 90 80 70 60 50 40 30 20 10 0

NOESY NMR (D_2O)

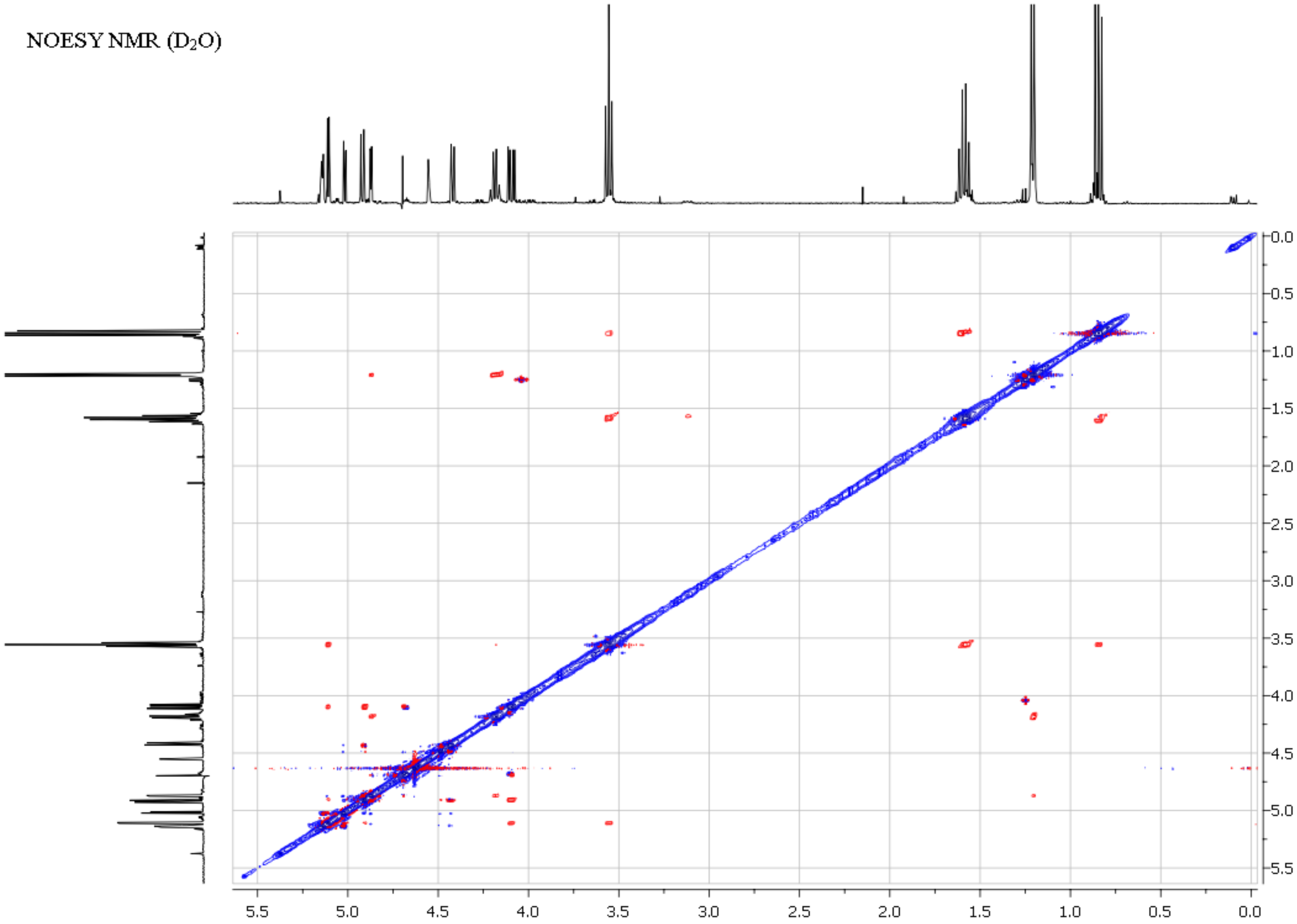

Acquisition Parameter

Source Type	ESI	Ion Polarity	Negative	Set Nebulizer	0.4 Bar
Focus	Not active			Set Dry Heater	180 °C
Scan Begin	50 m/z	Set Capillary	3200 V	Set Dry Gas	4.0 l/min
Scan End	3000 m/z	Set End Plate Offset	-500 V	Set Divert Valve	Waste

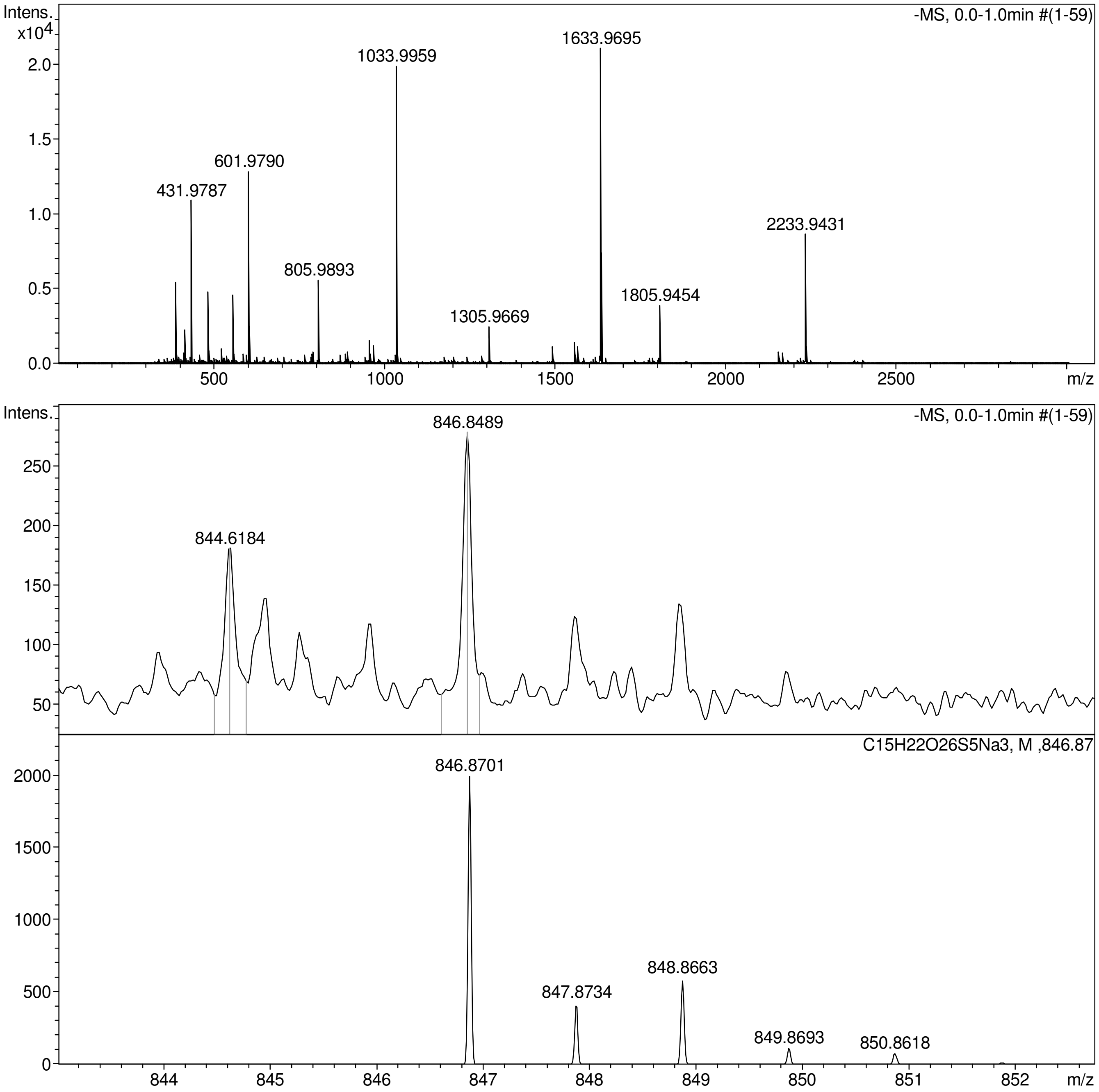

Supplement: Supplementary file 1 [file molecules-28-07571-s001.zip › molecules-2671385-supplementary.pdf]
